# Recent advances in designing high-performance and enzymatically degradable plastics

**DOI:** 10.1039/d6sc04609f

**Published:** 2026-08-18

**Authors:** Jiaxiong Liu, Xin Zhou, Hiroshi Uyama, Yoshinori Takashima

**Affiliations:** a Department of Macromolecular Science, Graduate School of Science, The University of Osaka 1-1 Machikaneyama Toyonaka Osaka 560-0043 Japan takasima@chem.sci.osaka-u.ac.jp; b Division of Applied Chemistry, Graduate School of Engineering, The University of Osaka 2-1 Yamadaoka Suita Osaka 565-0871 Japan uyama@chem.eng.osaka-u.ac.jp; c Forefront Research Center, Graduate School of Science, The University of Osaka 1-1 Machikaneyama Toyonaka Osaka 560-0043 Japan liuj22@chem.sci.osaka-u.ac.jp; d Innovative Catalysis Science Division, Institute for Open and Transdisciplinary Research Initiatives (ICS-OTRI), The University of Osaka 1-1 Yamadaoka Suita Osaka 565-0871 Japan

## Abstract

Plastics have become indispensable in modern life, yet their end-of-life waste continues to accumulate, causing severe environmental pollution and posing risks to ecosystems and human health. This urgent challenge has driven the development of sustainable plastics as alternatives to conventional persistent plastics, among which biodegradable plastics are particularly promising. Owing to their reduced environmental footprint and their ability to undergo mild biodegradation mediated by efficient enzymes, biodegradable plastics have attracted increasing attention from both academia and industry in recent years. Nevertheless, their practical implementation remains constrained by the persistent trade-off between material performance and degradability. Therefore, the rational design of biodegradable plastics that simultaneously combine high performance and enzymatic degradability is essential for improving their practicality and sustainability, thereby alleviating plastic pollution and advancing a circular plastics economy. In this review, we discuss recent advances, mainly since 2020, in achieving high-performance and enzymatically degradable plastics through chemical design, physical blending, and supramolecular interactions.

## Abstract 2

Rational design of high-performance and enzymatically degradable plastics is crucial to overcoming the performance–degradability trade-off, thereby enabling practical applications, reducing plastic waste, and advancing a circular plastics economy.

## Graphical abstract

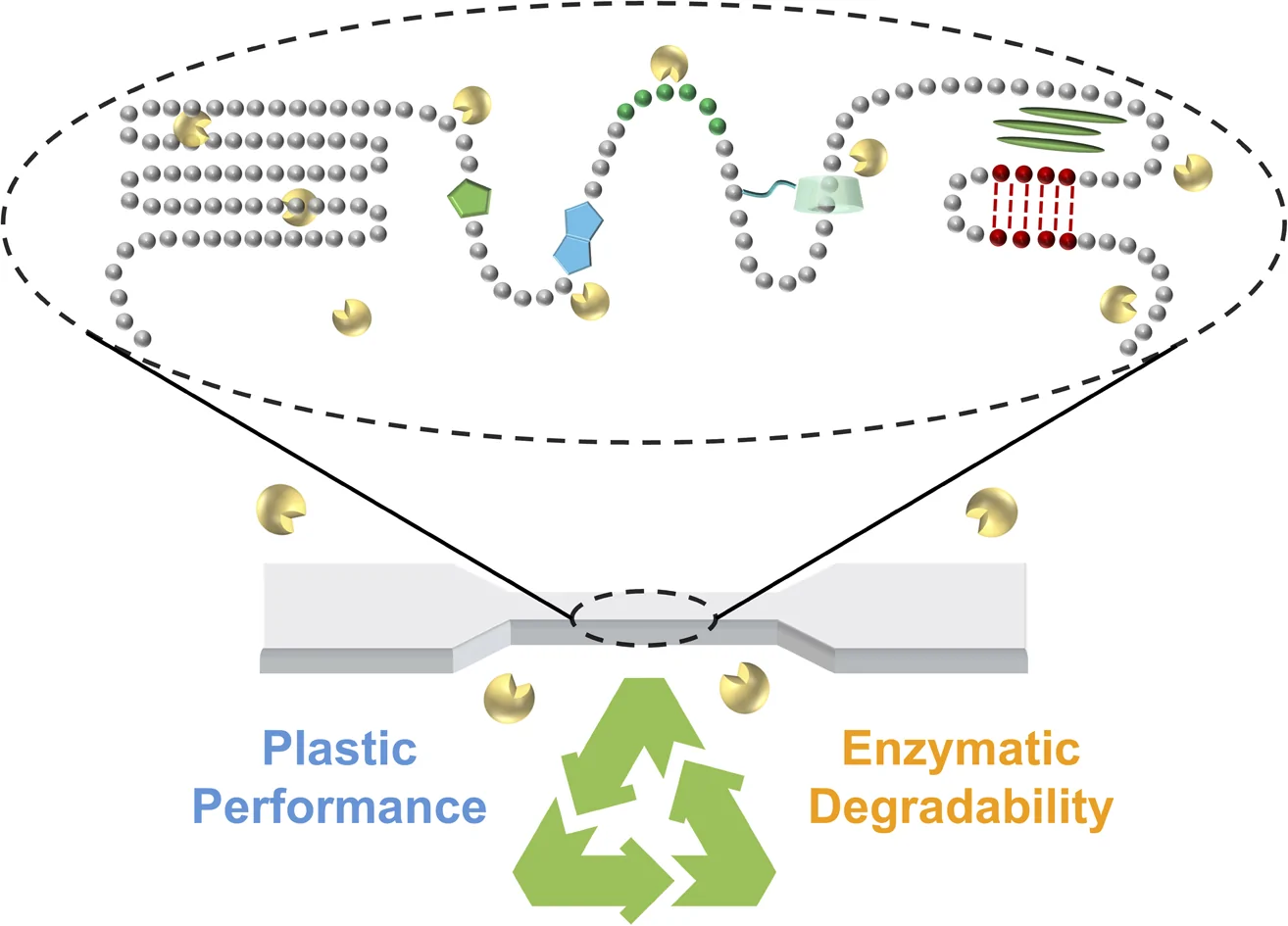

## 1. Introduction

### 1.1 Plastic pollution

Plastics have become indispensable materials in modern society, underpinning a broad range of applications in energy, construction, healthcare, housing, clothing, packaging, transportation, and communication. Driven by their light weight, durability, and versatile processability, global plastic production is expected to increase from 475 million tonnes (Mt) in 2022 to approximately 1200 Mt by 2060.^1^ However, the vast majority of commercial plastics remain derived from nonrenewable petroleum-based feedstocks, including polyethylene (PE), polypropylene (PP), poly(vinyl chloride) (PVC), polystyrene (PS), and poly(ethylene terephthalate) (PET).^2^ These materials are designed primarily for long-term performance rather than degradability or recyclability, leading to the escalating accumulation of persistent plastic waste after disposal.^1–3^ At present, less than 10% of plastic waste is recycled worldwide, whereas incineration and landfilling remain the dominant end-of-life treatment routes.^1–6^ The former generates substantial greenhouse gas emissions and toxic byproducts, thereby exacerbating climate change and environmental contamination,^7–9^ while the latter results in the long-term accumulation of plastic waste and the slow release of additives, microplastics and nanoplastics into terrestrial and aquatic environments, posing potential risks to soil and groundwater.^10–14^ Consequently, plastic pollution has emerged as one of the most pressing global environmental challenges that threaten ecosystems and human health, highlighting the urgent need to develop next-generation sustainable plastics with reduced environmental footprints.^15–26^ Among the various alternatives, biodegradable plastics are particularly attractive because they offer a promising route toward mitigating persistent plastic pollution while enabling more sustainable end-of-life management.^21–25^

### 1.2 Biodegradable plastics

**Fig. 1:**
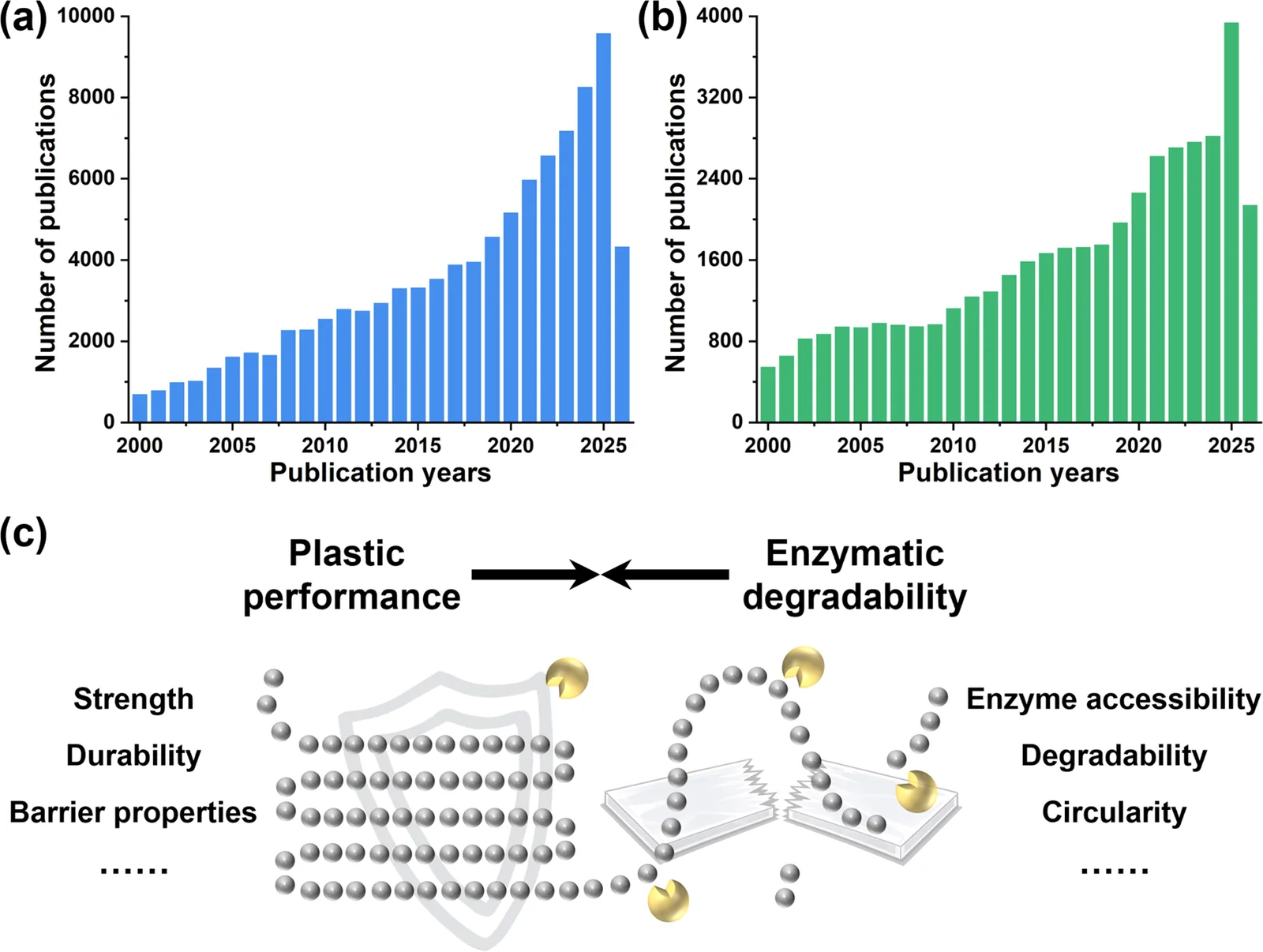
Annual numbers of publications from Scopus as of May 2026, calculated as the sum of publications containing the keywords of (a) “biodegradable plastics”, “biodegradable polymers”, and “biodegradable materials”, and of (b) “enzymatic degradation”, “enzymatic recycling”, and “enzymatic upcycling”. (c) Scheme illustrating the trade-off between plastic performance and enzymatic degradability.

Biodegradable plastics are generally defined as materials that can be decomposed by naturally occurring microorganisms, such as bacteria and fungi, or by enzymes under relatively mild environmental conditions.^21–23^ Compared with conventional persistent plastics, biodegradable plastics are considered more environmentally benign because they can reduce the long-term accumulation of plastic waste and facilitate reintegration into natural and material cycles, making them promising candidates for sustainable alternatives.^26–29^ Accordingly, both academic research and industrial production in this field have been expanding steadily in recent years, as evidenced by the sharp increase in related publications around 2020 (Fig. 1a).^22–29^ In parallel, the global biodegradable plastics market is projected to grow from approximately USD 1.0 billion in 2022 to USD 2.2 billion by 2028,^25^ driven by increasing environmental awareness, tightening regulations on single-use plastics, and growing demand for sustainable materials across packaging, agriculture, consumer goods, textiles, and biomedical applications.

Based on their origin and synthesis routes, biodegradable plastics can generally be classified into natural and synthetic biodegradable plastics.^24^ Starch^30^ and cellulose^31,32^ are currently the main commercial natural biodegradable plastics, whereas major synthetic biodegradable plastics include poly(lactic acid) (PLA),^33,34^ poly(butylene adipate-*co*-terephthalate) (PBAT),^35,36^ polyhydroxyalkanoates (PHAs),^37^ poly(butylene succinate) (PBS),^38^ poly(*ε*-caprolactone) (PCL),^39^ poly(propylene carbonate) (PPC),^40^ and poly(glycolic acid) (PGA).^41^ Most synthetic biodegradable plastics contain aliphatic ester bonds, which are susceptible to hydrolysis and enzymatic cleavage under appropriate environmental conditions. It should be noted that although some commercial plastics, such as PET and PE, have been reported to be enzymatically degradable, they are still generally considered non-biodegradable or persistent plastics because their degradation is usually slow, incomplete, dependent on specially treated substrates, and inefficient under mild conditions.^2,23,42–44^ Despite rapid progress, current commercial biodegradable plastics still face several limitations, including insufficient degradation rates and relatively poor performance and limited functionality compared with conventional commercial plastics.^18,23,45–48^ These drawbacks have hindered their practical implementation and broader market adoption, resulting in their currently low market share.^43^ Consequently, increasing research efforts have focused on the molecular and structural design of next-generation biodegradable materials that can simultaneously achieve high performance, functional versatility, and efficient biodegradability.^19,20^

### 1.3 Enzymes for plastic degradation

The enzyme-dominated nature of biodegradation has been a major driver of the growing interest in biodegradable plastics, because enzymatic processes are generally nontoxic, mild, and environmentally benign, in contrast to many metal- or organocatalyzed degradation processes that may involve toxic catalysts or harsh, energy-intensive conditions.^42–44,49–52^ Since the 1970s, extensive efforts have been devoted to identifying enzymes capable of polymer degradation, and an increasing number of such enzymes have been discovered, isolated, and purified from microorganisms for the effective degradation of various plastics.^43,49–53^ Environmental biodegradation in soil, marine environments, and composting also involves microbial and enzymatic processes, but generally occurs in complex and heterogeneous systems, often resulting in gradual and sometimes incomplete material breakdown, with the potential for prolonged environmental persistence and microplastic formation.^42,45,54,55^ By contrast, the use of specific enzymes under defined conditions can enable more controlled depolymerization with higher degradation rates and, in suitable polymer-enzyme systems, may facilitate the identification and recovery of degradation products for recycling or upcycling.^52,56–60^ Recent advances in protein engineering and machine learning have further accelerated the optimization and engineering of enzymes capable of attacking polymer backbones that were previously considered poorly biodegradable or nonbiodegradable, thereby greatly expanding the scope and development of enzymatic plastic degradation (Fig. 1b).^43,49,52,61–64^

**Fig. 2:**
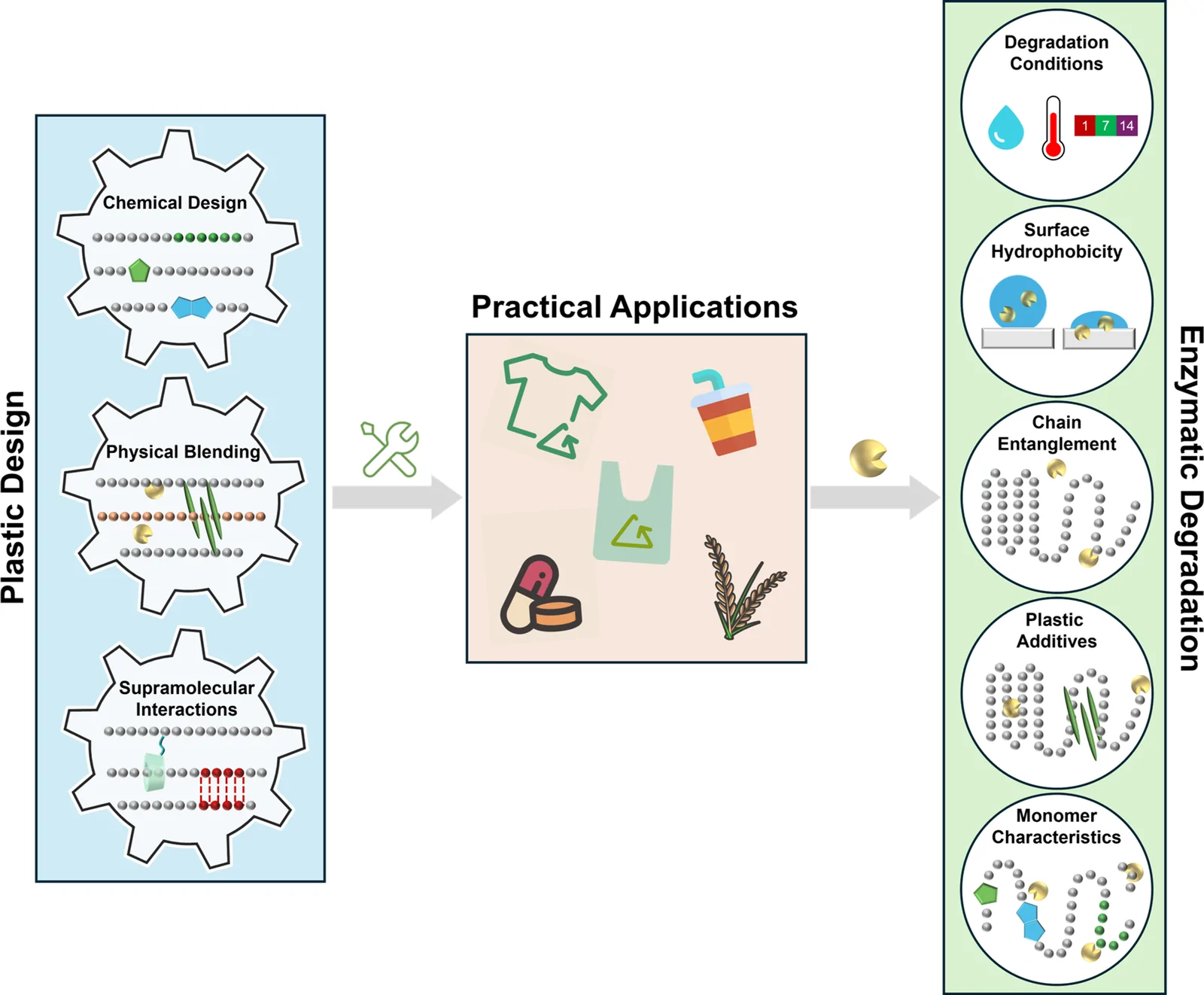
Conceptual scheme illustrating the design, applications, and enzymatic degradation of biodegradable plastics.

The efficiency of enzymes in plastic degradation is influenced, on the one hand, by the intrinsic activity and stability of the enzymes under degradation conditions (*e.g.*, solvent, temperature, and pH) and, on the other hand, to a considerable extent by the structural features of the plastic substrate (Fig. 2).^43,44,65–76^ In general, enzymatic degradation proceeds in aqueous solution and typically initiates as surface erosion of the plastic. Highly hydrophobic surfaces can hinder water and enzyme penetration, confining degradation to the exterior and slowing the process, whereas improved hydrophilicity facilitates deeper penetration and can promote bulk erosion.^43,65,66^ Chain entanglement and crystallinity further affect degradation, as enzymes preferentially attack amorphous and less entangled regions, making low-crystallinity and flexible plastics more susceptible to degradation.^43,65–67^ For polymer blend systems, additives and the microphase morphology of the blend can also significantly influence enzymatic degradation behavior.^64,68–70^ From a microscopic perspective, enzymatic continuity and degradation efficiency can be affected by the compatibility between polymer chains and enzyme active sites,^70,71^ the hydrolytic susceptibility and rigidity of the components,^56,72,73^ and the characteristics of the degraded products.^74^ Therefore, plastics containing potentially enzyme-cleavable bonds, such as polyesters and biopolymer-based plastics, are not necessarily enzymatically degradable,^43,75,76^ and claims of enzymatic degradability should be supported by experimental evidence. This highlights the practical importance of designing plastics that combine sufficient mechanical performance with experimentally demonstrated enzymatic degradability.

### 1.4 Challenges and scope

Designing plastics with an emphasis on enhanced biodegradability often comes at the expense of their essential performance attributes, thereby limiting their practical utility (Fig. 1c).^77–79^ For instance, increasing surface hydrophilicity can facilitate water and enzyme penetration, but it may also promote unintended water uptake and microbial invasion under ambient service conditions, severely weakening barrier performance and durability. Furthermore, reducing molecular weight and crystallinity can decrease chain entanglement and improve enzyme accessibility, but often compromises mechanical strength and stiffness of plastics, thereby rendering the materials unsuitable for many practical applications. This inherent conflict epitomizes the critical trade-off confronting biodegradable plastics, underscoring the central challenge of rationally designing their chemical and physical architecture to balance plastic performance with enzymatic degradability. Achieving this balance is essential for advancing plastics that combine high performance with sufficient enzymatic degradability, thereby enabling their broader practical use as next-generation sustainable plastics.

Many excellent reviews have discussed enzymes for plastic degradation, with most focusing on enzyme discovery and classification, degradation mechanisms, and enzyme optimization or engineering to enhance plastic degradation.^42–44,49–52^ Although there are reviews focused on plastic design for biodegradation, they often emphasize degradability while giving less attention to how such designs affect material performance and functionality,^17,18,22^ which are not practical for real applications. For both biodegradable plastics and enzymatic degradation, addressing the plastics performance-enzymatic degradability trade-off is crucial for their practical application and development. Given the rapidly growing research interest in biodegradable plastics and enzymatic degradation in recent years (Fig. 1a and b), this review primarily covers advances since 2020 in the rational design of plastics that maintain or enhance material performance while enabling or improving enzymatic degradation through chemical design, physical blending, and supramolecular interactions (Fig. 2).

## 2. Chemical design

The intrinsic chemical structure of plastics fundamentally governs their physical properties, functionality, and degradability.^16–25^ In this section, we summarize and discuss how polymer structures can be rationally tailored through chemical design, including the development and modification of bioplastics, the use of bio-based feedstocks, and backbone sequence control, to simultaneously tune plastic performance and enzymatic degradability.

### 2.1 Development and modification of bioplastics

Naturally derived biomacromolecules, such as polysaccharides, proteins, and DNA, often combine favorable mechanical properties, excellent biocompatibility, and intrinsic biodegradability, making them attractive sustainable and renewable alternatives to petroleum-based plastics. Considerable progress has been made in their preparation, applications, and degradation, as summarized in several excellent reviews.^19,21,30–32,78–81^ Here, we focus on representative cellulose-, silk fibroin-, and DNA-based bioplastics for which enzymatic degradation has been systematically investigated.

#### 2.1.1 Cellulose

**Fig. 3:**
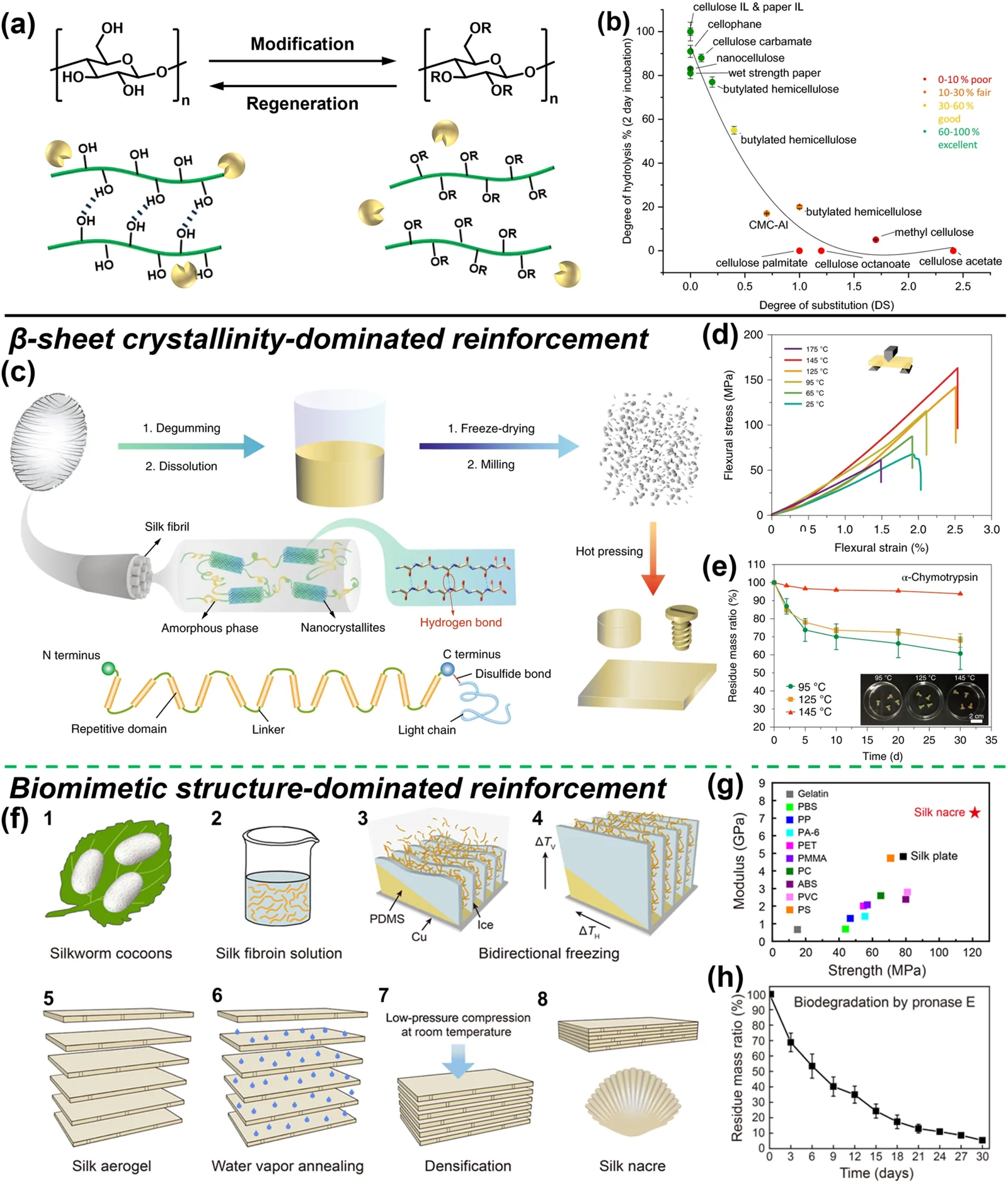
(a) The modification and regeneration of cellulose and their influences on enzymatic degradation. (b) The relationship between enzymatic degradation and the DS of cellulose. (Adapted from ref. 75 under a CC BY 4.0 license). (c) Preparation, (d) mechanical properties, and (e) enzymatic degradation of RSF bioplastics with β*-*sheet crystallinity-dominated reinforcement. (Reproduced with permission from ref. 94. Copyright 2020, Springer Nature). (f) Preparation, (g) mechanical properties, and (h) enzymatic degradation of RSF bioplastics with biomimetic structure-dominated reinforcement. (Reproduced from ref. 98 under a CC BY-NC 4.0 license).

As the most abundant plant-derived polymer, cellulose has been extensively explored as a key platform for bioplastics owing to its intrinsic renewability, biocompatibility, and biodegradability.^31,32,79,80^ Native cellulose contains a rigid backbone and dense intermolecular hydrogen-bonding network, which imparts excellent mechanical strength but severely limit dissolution and processing.^31,32^ To overcome these limitations, derivatization and surface modification of the abundant hydroxyl groups are widely employed to improve processability and expand functionality.^31,32^ However, such modifications inevitably alter hydrogen bonding, crystallinity, and enzyme accessibility, thereby affecting both mechanical properties and biodegradability. Precise control over substituent structure and degree of substitution (DS, defined as the average number of substituted hydroxyl groups per a glucose unit) is therefore essential for balancing performance and enzymatic degradability (Fig. 3a).^75,82^

To clarify how chemical modification influences cellulose properties and biodegradation, Orelma and co-workers systematically investigated 14 cellulose-based films, including pure and chemically modified celluloses.^75^ Chemical substitution weakened cellulose chain packing, generally lowering tensile strength and Young's modulus while increasing ductility, with the magnitude depending on DS and side-chain structure. Using an enzyme mixture containing cellulase, mannanase, xylanase, and β-glucosidase in sodium acetate buffer (pH 5.0) at 40 °C, the degree of hydrolysis after 48 h, as quantified by the release of reducing sugars relative to the initial cellulose content, decreased exponentially with increasing DS and was also strongly affected by substituent type and length, because substituents sterically hindered cellulase binding and access to the cellulose backbone (Fig. 3b). Pilot-scale composting confirmed this trend, as readily hydrolyzed or low-substituted films disintegrated rapidly, whereas cellulose acetate, palmitate, and octanoate showed negligible degradation over 12 weeks. Furthermore, Harlin and co-workers acetylated cellulose paper surfaces by immersion in acetic anhydride/pyridine mixtures and found that increasing DS enhanced hydrophobicity and induced a continuous transition from fibril surface 6-acetylation to the formation of cellulose triacetate (CTA) domains.^83^ These CTA domains acted as a semi-continuous elastic phase within the fibril bundles, increasing the wet-state tensile strength from 5.2 to 23.7 MPa and fracture strain from 7.3 to 10.7%. However, acetylation, particularly the formation of CTA domains, markedly reduced mass loss from 78% to 0–1.6% during enzymatic treatment by limiting swelling and hindering enzyme penetration. Iwata and co-workers modified cellulose with varying acyl DS and PLA side-chain lengths.^84^ For CePr-g-PLA films, increasing DS strengthened intermolecular interactions, thereby enhancing thermal stability and Young's modulus. Increasing the acyl-chain length from acetyl to hexanoyl enhanced internal plasticization, making the films softer and more flexible with improved extensibility. However, these side chains strongly impeded enzymatic degradation, and appreciable cellulose degradation occurred only after PLA side-chain removal by proteinase K. Subsequent treatment with a cellulase mixture in sodium acetate buffer (pH 5.0) at 50 °C revealed that lower DS and shorter acyl chains exhibited significantly greater total mass loss. Related strategies were also applied to paramylon to regulate its properties and enzymatic degradability.^85^

The above studies collectively demonstrate that chemical cellulose modification and DS can effectively tune its mechanical properties, but often at the cost of reduced enzymatic accessibility and biodegradation (Fig. 3a). In this regard, Zhang and co-workers partially or completely deacetylated commercial cellulose acetate (CA, initial DS ≈ 2.5) by saponification.^86^ Reducing the DS from 2.5 to 0 increased the enzymatic hydrolysis rate from <20% to ∼100%, which was attributed to reduced steric hindrance from acetyl groups and increased hydrophilicity, thereby facilitating enzyme access to the cellulose backbone. The treated cigarette-filter fibers retained their fibrous morphology with surface pores and could tightly integrate with waste kraft pulp, forming a reinforced network that increased the tensile strength of recycled paper to approximately 1.6 times that of kraft paper. A similar deacetylation concept was also applicable to chitin whiskers, where it accelerated degradation in phosphate buffer (pH 7.4) at 37 °C with lysozyme, lipase, or their mixture, with the mixed-enzyme system showing the strongest synergistic effect.^87^

Recently, water-assisted processing has emerged as an effective strategy to improve the processability of cellulose by using water as a reversible plasticizer to temporarily disrupt the intermolecular hydrogen-bonding network and enhance chain mobility, while retaining its inherent mechanical robustness and biodegradability.^88–91^ He and co-workers deacetylated CA to obtain a highly amorphous regenerated cellulose hydroplastic with tunable wet–dry mechanical properties.^89^ At ∼45 wt% water content, reduced cellulose hydrogen bonding and an interconnected water network rendered the material flexible and ductile, with a fracture strain of 121% and a tensile strength of 21.3 MPa. Drying to ∼10 wt% water restored high stiffness, affording a fracture stress of 64.8 MPa and a Young's modulus of 6.8 GPa that surpassed many commodity plastics and green thermoplastics. The material could be repeatedly reshaped through wet-dry cycling over 20 cycles without significant mechanical loss. Despite its relatively low surface area, the hydroplastic sheet exhibited degradation comparable to commercial α-cellulose powder with cellulase in HAc/NaAc buffer (pH 4.8) at 50 °C, likely owing to its low crystallinity and abundant enzyme-accessible amorphous regions. However, the degradation assessment was solely based on sugar release during enzymatic hydrolysis, which may not fully capture the actual degradation extent of the two cellulose materials. Meanwhile, most studies reported only reducing sugar release or mass loss without determining the yields and molecular identities of the soluble products or distinguishing monosaccharides from oligosaccharides, thereby limiting their potential recovery and utilization.

#### 2.1.2 Silk fibroin

Proteins, including gelatin, silk fibroin, soy protein, casein, whey protein, keratin, elastin, and sericin, have been widely explored as sustainable materials owing to their natural abundance, intrinsic biocompatibility, and well-defined structures.^78–80^ Among them, silk fibroin (SF) is particularly attractive because it combines high stiffness and toughness, making it a promising candidate for sustainable structural plastics.^78,92,93^ These properties arise from a hierarchical structure of β-sheet nanocrystals dispersed in an amorphous matrix, where the β-sheet domains impart strength and stiffness and the amorphous regions afford stretchability and toughness. Consequently, considerable efforts have focused on regulating the β-sheet content and hierarchical organization to tune both the mechanical properties and biodegradability of SF-based bioplastics.

Kaplan and co-workers developed a thermal molding strategy to convert regenerated silk fibroin (RSF) into robust structural bioplastics.^94^ RSF was first processed into amorphous silk nanomaterials (ASNs), in which bound water lowered the water-associated *T*_g_ and increased segmental mobility, thereby acting as an intrinsic plasticizer for high-pressure thermal molding (Fig. 3c). This process promoted β-sheet crystallization and allowed mechanical properties to be tuned by processing temperature (Fig. 3d). The best samples molded at 145 °C exhibited a specific strength of ∼109 MPa g^−1^ cm^3^, surpassing many natural structural materials and approaching some synthetic polymers. Notably, samples molded at lower temperatures exhibited greater mass loss with protease XIV or α-chymotrypsin in phosphate buffer at 37 °C, likely because their lower crystallinity facilitated water and enzyme penetration (Fig. 3e). Owing to their good biocompatibility, RSF bioplastics are also promising for implantable devices and for the incorporation of functional agents such as enzymes and antibiotics. Recently, they further developed a plasticizer-assisted thermal molding of RSF at mild temperature (∼60 °C) to produce mechanically robust living plastics with viable embedded microorganisms.^95^ Plasticizer (water or glycerol) increased chain mobility and drove random-coil to β-sheet transitions, tuning RSF from compliant hydrated materials with high extensibility (∼20%) and toughness (∼1.62 MJ m^−3^) to dehydrated β-sheet-rich specimens with greatly increased Young's modulus from 0.35 to 1.08 GPa. The mild processing conditions further allowed the stable encapsulation of living microorganisms into biocompatible RSF as engineering living bioplastics.

Maniglio and co-workers developed a chemical crosslinking method to preserve the mechanical integrity of solid SF monoliths under physiological conditions, using bio-derived genipin to form covalent bridges that reduced water-induced plasticization and partially restored wet-state performance.^96^ The crosslinked network restricted chain mobility and strengthened the structure, giving a 318% increase in compressive modulus compared with genipin-free materials with similar crystallinity. However, genipin crosslinking markedly reduced mass loss and structural disintegration with protease XIV in phosphate buffer (pH 7.4) at 37 °C, likely by limiting swelling and maintaining interlayer cohesion. To better exploit crosslinking strategies to enhance SF performance, Chen and co-workers constructed a dual-crosslinking RSF fiber system combining light-induced covalent crosslinks and metal-coordination crosslinks.^97^ Together with post-stretching, this dual-crosslinking architecture promoted chain alignment, increased β-sheet content, and generated a dense interconnected network, affording high tensile strength of ∼891 MPa, Young's modulus of ∼15 GPa, and toughness of ∼114 MJ m^−3^. Importantly, the dual-crosslinking RSF sutures remained susceptible to elastase treatment in phosphate buffer (pH 7.4) at 37 °C, with the mass loss profile influenced by both suture diameter and crosslinking state. Their biocompatibility and controllable degradation make them promising for biomedical applications such as sutures, artificial vessels, and hernia patches. Despite improved mechanical performance, covalent crosslinking may generate peptide fragments containing residual cross-linker structures, leaving their effects on the subsequent degradation, recovery, and recyclability of these degradation products unclear.

In contrast to crystallinity-dominated reinforcement, Bai and co-workers reported a single-component RSF bioplastic with a biomimetic nacre-like hierarchical architecture.^98^ This architected “brick-and-mortar” morphology, controlled by bidirectional freezing, water-vapor annealing, and densification conditions (Fig. 3f), played a dominant role in mechanical reinforcement, enabling silk nacre to achieve a bending strength of 125 MPa and a modulus of 6.8 GPa, outperforming homogeneous solvent-cast silk plates and many synthetic polymers (Fig. 3g). Notably, these improvements were achieved despite a lower β-sheet fraction than that of the silk plate, indicating that macroscopic architecture can outweigh local crystallinity in governing mechanical performance. Densified interfaces enhanced breaking strength, while the lamellar structure and elongated silk laminae effectively retarded the fracture process. The material also exhibited controllable steam-induced plasticity, while its residual mass decreased to nearly zero with pronase E in phosphate buffer at 37 °C for 30 days (Fig. 3h).

To expand the functional applications of SF-based materials, Kumar and co-workers developed a mild chemical strategy to graft perfluoroalkyl chains onto SF.^99^ The resulting films showed water contact angles of up to ∼125°, exceeding those of PTFE, together with markedly reduced water uptake and preserved mechanical integrity after long-term incubation in aqueous media. They further developed hydrophobic silk films as platelet-compatible antifouling coatings for biomedical applications.^100^ The fluorinated SF largely retained its secondary structure and mechanical properties under aqueous conditions, yet remained biodegradable, as evidenced by a reduction in molecular weight from approximately 60 to 30 kDa within 24 h with protease XIV in phosphate buffer at 37 °C. The highly hydrophobic SF coatings reduced bacterial adhesion and biofilm formation and minimized platelet adhesion and activation, thereby helping preserve platelet function during storage. However, the multistep chemical modification, the persistence of approximately 30 kDa fluorinated macromolecules after enzymatic treatment, and the unresolved identity and fate of released fluorinated fragments raise concerns about the sustainability and true biodegradability of this strategy.

The above studies have demonstrated that SF can be developed into bioplastics with both excellent mechanical performance and enzymatic degradability, yet the fate and utilization of the degradation products remain largely unexplored. Proteases can cleave SF at enzyme-dependent sites to generate shorter peptides, which could be further hydrolyzed by aminopeptidases into free amino acids as valuable feedstocks for potential recycling or upcycling.^101–103^ Notably, Stellacci and co-workers demonstrated that protein mixtures containing SF could be degraded into free amino acids through sequential treatment with thermolysin and leucine aminopeptidase, enabling their reuse for the cell-free synthesis of new functional proteins.^102^ They subsequently extended this strategy by converting the recovered amino acids into an entirely different protein-based hydrogel, which could be enzymatically degraded again to recover amino acids, thus demonstrating the circularity of protein-based materials.^103^ This proof-of-concept highlights the potential of coupling enzymatic degradation with recycling or upcycling strategies for SF-based bioplastics.

#### 2.1.3 DNA

DNA is an abundant biopolymer obtainable from plants, animals, and microorganisms, and has increasingly been explored as a biocompatible and biodegradable material for diverse applications.^81,104–106^ However, DNA-based materials have thus far been explored predominantly as hydrogels for biomedical applications,^105^ and have only recently emerged as promising degradable bioplastics.^81,106^

**Fig. 4:**
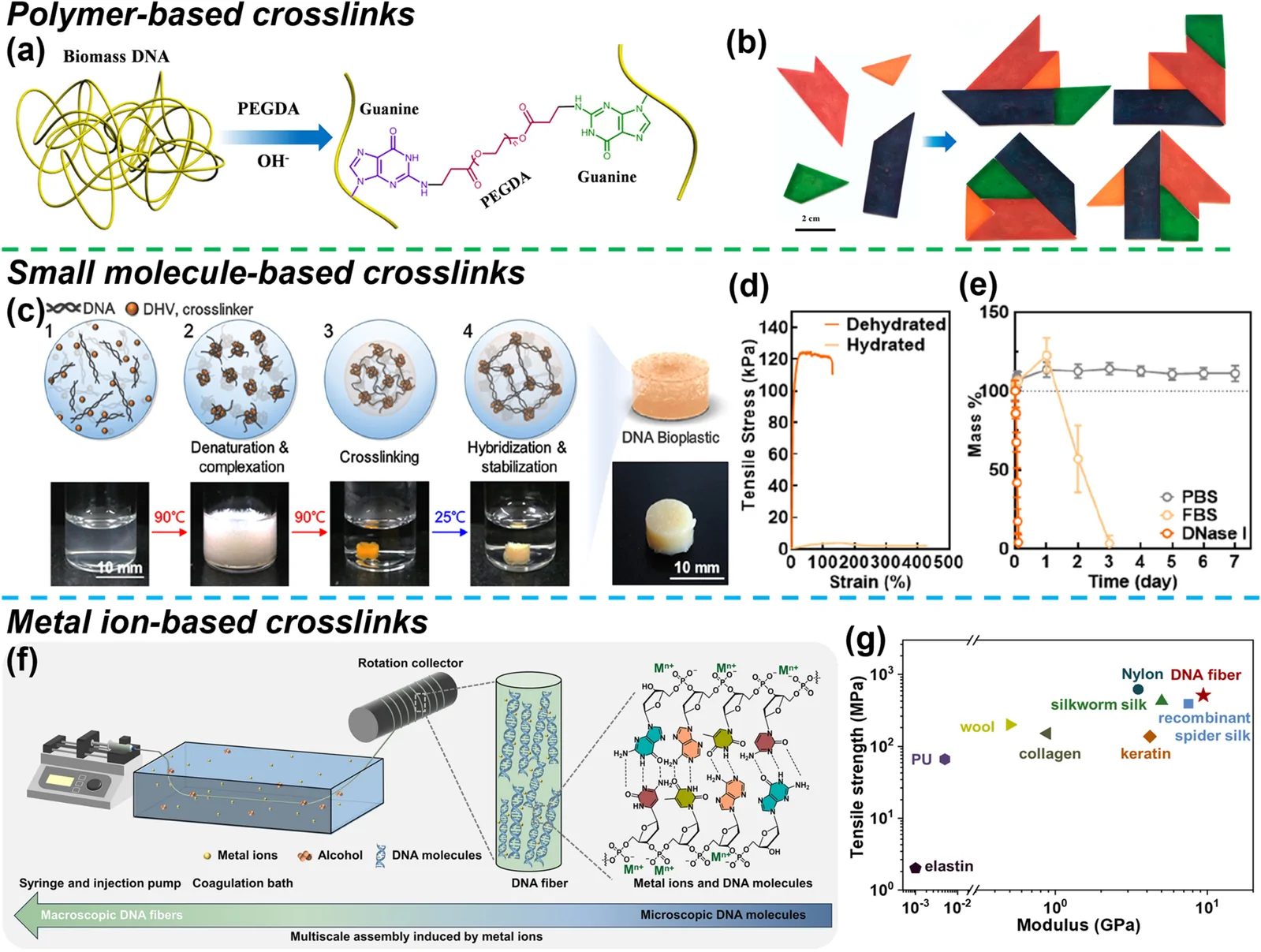
(a) Preparation and (b) different shapes of DNA bioplastics with polymer-based crosslinks. (Reproduced with permission from ref. 107. Copyright 2020, American Chemical Society). (c) Preparation, (d) mechanical properties, and (e) enzymatic degradation of DNA bioplastics with small molecule-based crosslinks. (Reproduced with permission from ref. 111. Copyright 2024, American Chemical Society). (f) Preparation and (g) mechanical properties of DNA bioplastics with metal ion-based crosslinks. (Reproduced with permission from ref. 112. Copyright 2024, Cell Press).

Luo and co-workers developed a universal one-step strategy to directly employ DNA as polymer backbone to construct three-dimensional networks *via* the chemical crosslinks between poly(ethylene glycol) diacrylate (PEGDA) and DNA (Fig. 4a), enabling the fabrication of hydrogels, organogels, composite membranes, and plastics.^107^ After dehydration of the DNA hydrogel, the resulting bioplastics could be processed into daily-use objects with various shapes (Fig. 4b). These DNA-based materials exhibited good biocompatibility and can be completely degraded into oligonucleotides by DNase I in digestion solution at 37 °C. Yang and co-workers subsequently fabricated DNA gels through a physical crosslinking strategy based on electrostatic and hydrophobic interactions between DNA and biomass-derived elastomeric ionomer.^108^ Three-dimensional porous DNA plastics were then obtained by freeze-drying, and their mechanical properties could be tuned from flexible to rigid by adjusting the DNA content, with a broad Young's modulus range of 2.0–21.2 MPa. These bioplastics also exhibited excellent recyclability and water-triggered self-healing and welding, while the remaining volume decreased to nearly zero within approximately 4 h in DNase I solution at 37 °C. To develop a more sustainable DNA-based bioplastic, Hu and co-workers constructed renewable polysaccharide/DNA crosslinking hydrogel networks based on reversible imine covalent bonds, which served as precursors for bioplastics.^109^ The resulting materials showed excellent organic-solvent resistance while retaining aqueous recyclability and self-healing capability. Their mechanical properties could be tuned by varying polysaccharide composition and processing conditions; notably, dense bioplastics prepared by room-temperature drying reached a Young's modulus of ∼1155 MPa. Importantly, the bioplastics gradually decreased in size and visually disappeared within 120 min in DNase I solution and within 29 days when buried in soil. Life-cycle assessment further indicated that producing 1 m^3^ of bioplastic with 10 recycling cycles generated a carbon footprint of 861 kg CO_2_-eq. m^−3^, 58.7–78.3% lower than many commercial plastics without recycling.

In addition to polymers, small molecule crosslinkers can also be used to construct DNA bioplastics. Lee and co-workers employed 1,1′-diheptyl-4,4′-bipyridinium (DHV) to bind the DNA backbone through minor-groove binding and charge interactions, thereby sequentially preparing DNA gels and DNA bioplastics (Fig. 4c).^110,111^ The high-density ssDNA-DHV crosslinking points allowed the bioplastics to retain shape and mechanical integrity in various solvents, while their properties could be tuned from robust to stretchable by varying the crosslink density and hydration state (Fig. 4d). These materials exhibited reversible thermal responsiveness, self-healing, and thermoplastic processability, and their cytocompatibility supported potential biomedical applications, including artificial vessels, surgical staples, tissue-defect restoration, and on-demand degradable implants. Despite the highly crosslinked structure of the bioplastics, their mass decreased to nearly zero within ∼3 h in DNase I solution at 37 °C or within ∼3 days in 50% serum (Fig. 4e). Although the intact bioplastics showed limited cytotoxicity, the fate and toxicity of DHV and other degradation products remain unclear, particularly because DHV exceeded DNA by mass and has a paraquat-like bipyridinium structure.

Metal ion-mediated multiscale crosslinks provide another route to toughen DNA bioplastics, as demonstrated by Liu and co-workers (Fig. 4f).^112^ Metal ions acted as counterions to shield DNA phosphate charges, stabilize DNA conformation, and promote intermolecular crosslinks, thereby driving the assembly of DNA molecules into nanobundles and macroscale fibers. Post-stretching further enhanced molecular orientation and reduced intermolecular spacing, leading to synergistically improved mechanical properties. Among the tested metal ions, Mg-DNA produced the best performance, with a tensile strength of ∼513 MPa and a Young's modulus of ∼9.4 GPa, surpassing some commercial plastics and natural materials (Fig. 4g). Mg-DNA fibers were rapidly degraded by DNase at 37 °C despite their densely assembled structure, while the successful fabrication of fibers up to 1000 m in length further demonstrates their potential as tough and degradable structural materials.

Through diverse crosslinking strategies, renewable DNA can serve as polymer backbones for constructing bioplastics with tunable mechanical properties, functionality, and enzymatic degradability. DNase I randomly hydrolyzes phosphodiester bonds in the DNA backbone, generating oligonucleotide fragments with 5′-phosphate and 3′-hydroxyl termini, which could potentially be reused and re-ligated to prepare new DNA-based materials.^113–115^ However, these oligonucleotides were generally not isolated or quantitatively characterized, and their size distribution, yield, purity, and recyclability remain unclear. Moreover, in crosslinked systems, the fate of non-DNA components and their potential effects on the recovery and reuse of DNA-derived oligonucleotide remain largely unresolved. Future studies should therefore focus on the isolation and detailed characterization of DNA-derived degradation products and evaluate their potential reuse as feedstocks for sustainable and circular DNA bioplastics.

### 2.2 Eco-friendly feedstock substitution

Beyond macromolecular biomass, biomass-derived feedstocks are also critical factors for developing next-generation sustainable plastics.^17–19,116,117^ These renewable and generally low-toxicity building blocks provide promising alternatives to petroleum-based monomers, thereby improving the sustainability of both polymer production and the resulting materials.^116–120^ Moreover, the use of such eco-friendly feedstocks enables effective tuning of the performance and degradation behaviors of bio-based plastics, allowing them to achieve properties comparable to those of commercial plastics with enhanced biodegradability, as summarised in Tables 1–3.

**Table 1:** Effects of structural modification on the properties and enzymatic degradability of FDCA-based polymers

Polymers	Effects on physical properties	Effects on enzymatic degradation	Ref.
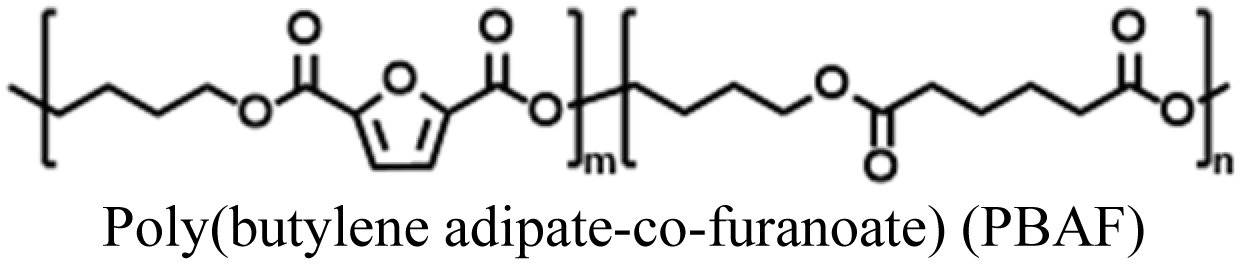	Replacing terephthalic acid (TPA) with FDCA maintained tensile strength and imparted remarkable elasticity, enhanced chain entanglement and lowered activation energy for segmental motion, but reduced Young's modulus and aromatic-domain *T*_m_	Replacing TPA with FDCA improved enzymatic degradation, raising mass loss from ∼0% (PBAT50) to >80% (PBAF50) with *Thermomyces lanuginosus* lipase in phosphate buffer (pH 6.5) at 50 °C within 3 days	71
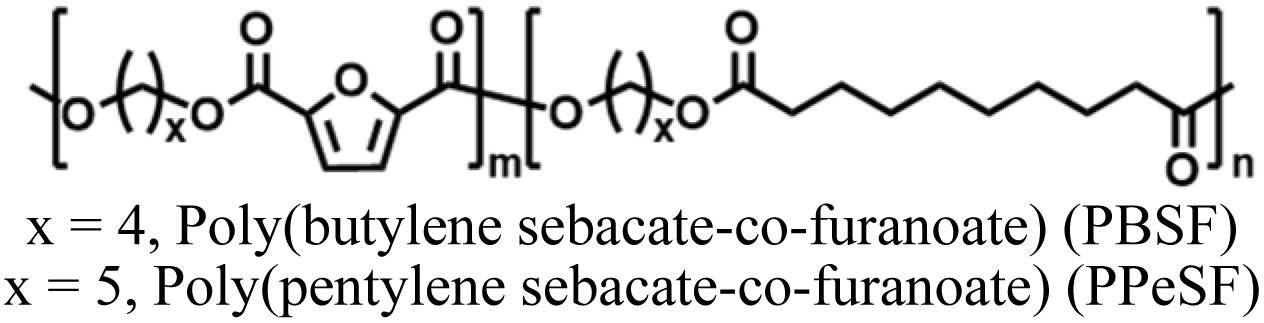	Replacing TPA with FDCA enhanced elastic recovery while reducing *T*_m_ and crystallinity. Even-carbon BDO (*x* = 4) favored chain packing and semicrystalline structures, whereas odd-carbon PDO (*x* = 5) disrupted crystallization and led to amorphous structures	PPeSF showed ∼35% mass loss with *Candida rugosa* lipase in phosphate buffer (pH 7.0) at 37 °C after 30 days	129
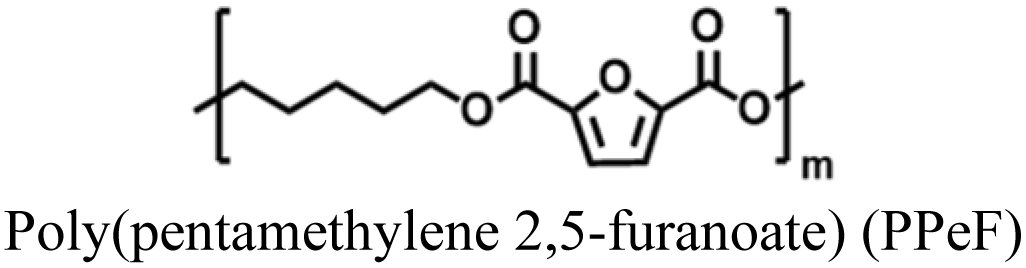	Increasing *M*_n_ enhanced *T*_g_, Young's modulus, fracture strain, gas barrier properties, and elastic recovery rate before full crystallization, but reduced crystallization rate and Δ*H*_m_	Increasing *M*_n_ suppressed enzymatic degradation, reducing mass loss from 20.8% to 14.0% with CALB in phosphate buffer (pH 7.4) at 37 °C after 30 days	130
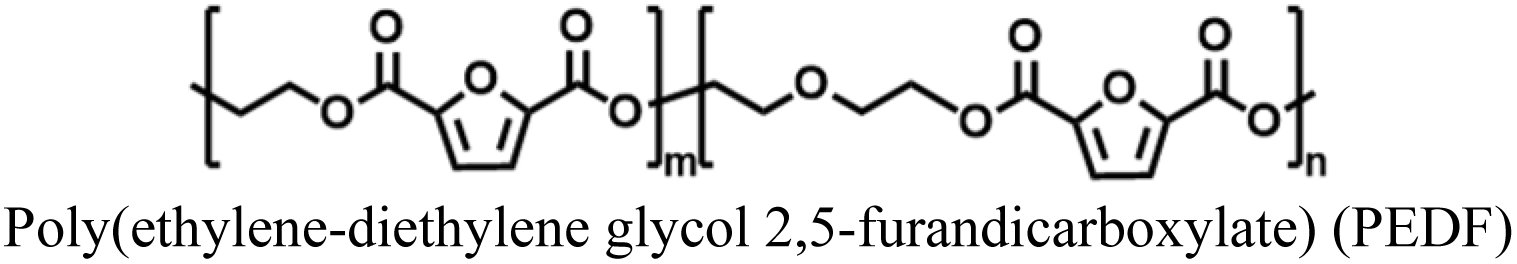	Increasing diethylene glycol (DEG) content significantly enhanced stretchability and hydrophilicity, but reduced *T*_g_, tensile strength, modulus, and gas barrier properties	Increasing DEG content improved enzymatic degradation, raising mass loss from negligible to 45.8% with CALB in phosphate buffer (pH 8.0) at 37 °C after 49 days	131
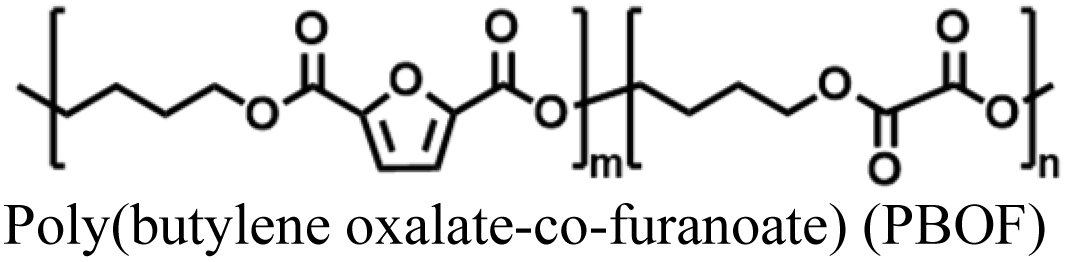	Increasing butylene furanoate (BF) content up to 41 mol% enhanced *T*_g_ and stretchability but markedly reduced stiffness, whereas further increasing BF content to ≥69 mol% increased *T*_g_, stiffness, crystallinity, and barrier properties but reduced stretchability	Increasing BF content suppressed enzymatic degradation, reducing mass loss from ∼100% to negligible with CALB in phosphate buffer (pH 7.4) at 37 °C after 30 days	56
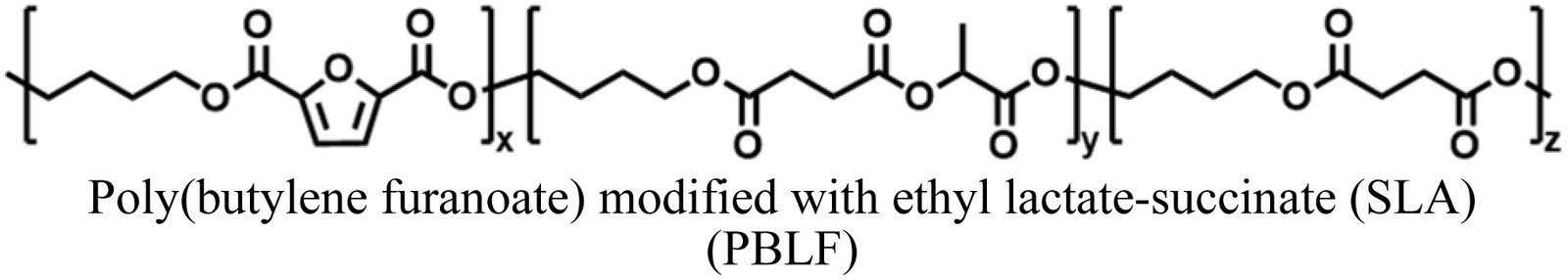	Increasing SLA content enhanced stretchability and elastic recovery at moderate SLA contents, but reduced *T*_g_, stiffness, and tensile strength. Micro-crosslinking markedly enhanced stiffness while retaining elastic recovery and thermal stability	Increasing SLA contents improved enzymatic degradation, raising mass loss from negligible to ∼100% with CALB in phosphate buffer at 37 °C after 28 days. Micro-crosslinking had little effect on enzymatic degradability	135
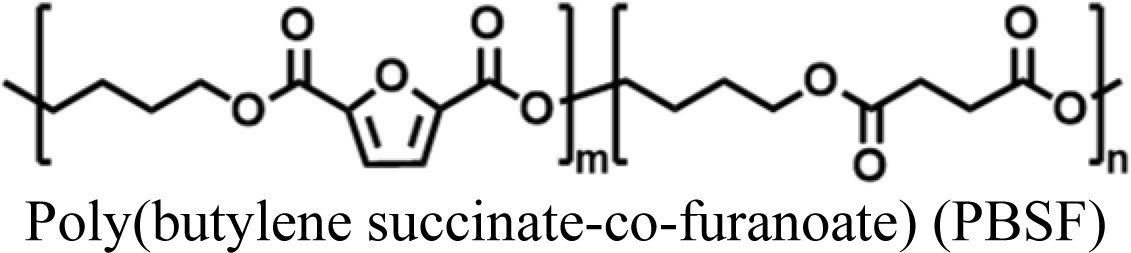	Increasing FDCA content enhanced melt flowability, hydrophilicity and fracture strain, but progressively reduced crystallinity. Strength improved at 15 mol% FDCA but declined at higher FDCA content	Increasing FDCA content improved enzymatic degradation, raising mass loss from 86.43% to ∼100% with CALB in phosphate buffer (pH 7.4) at 37 °C after 12 days	136

#### 2.2.1 2,5-Furandicarboxylic acid

Biomass-derived 2,5-furandicarboxylic acid (FDCA) is widely regarded as a promising renewable substitute for petrochemical terephthalic acid (TPA) because of their structural similarity.^118,121^ FDCA-based polymers containing furan rings have been extensively explored as homopolymers and copolymers for replacing TPA-derived plastics, providing opportunities to develop tough and enzymatically degradable materials.^122–125^ In general, the inherent rigidity of the FDCA unit reduces segmental mobility along the polymer chain, thereby increasing material stiffness while also affecting enzymatic accessibility and degradation behavior (Table 1).

**Fig. 5:**
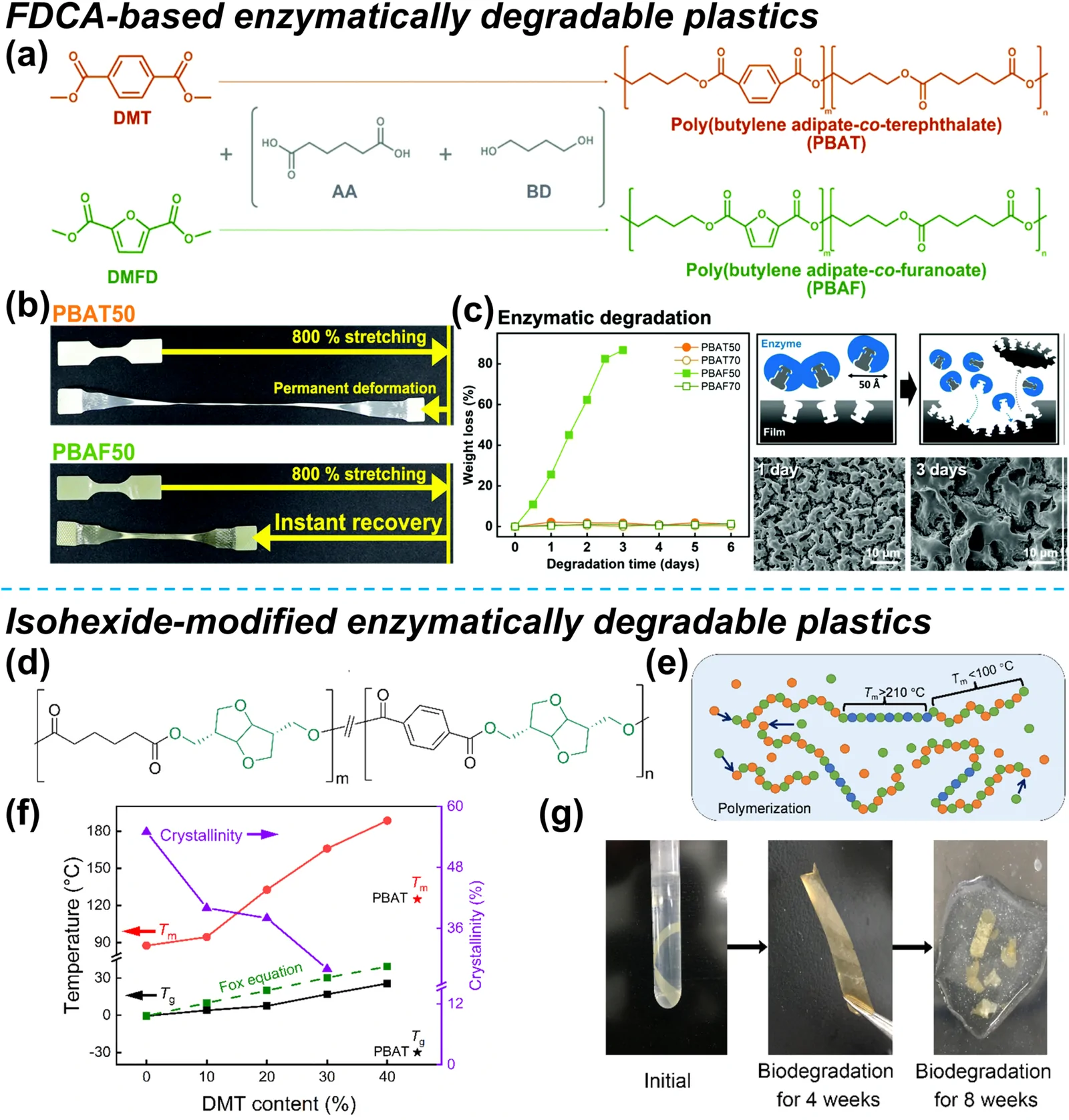
The comparison of (a) polymerization, (b) elastic recovery properties, and (c) enzymatic degradation between PBAT and FDCA-based enzymatically degradable plastics. (Reproduced from ref. 71 under a CC BY-NC 3.0 license). (d) Chemical structure, (e) blocky microstructure, (f) crystallinity and thermal properties, and (g) enzymatic degradation of isohexide-modified enzymatically degradable plastics. (Reproduced with permission from ref. 139. Copyright 2022, American Chemical Society).

To replace TPA in PBAT, Koo and co-workers prepared poly(butylene adipate-*co*-furanoate) (PBAF) through a two-step melt polymerization similar to that used for PBAT (Fig. 5a).^71^ The resulting PBAF exhibited molecular weights and tensile strength comparable to those of PBAT, but showed a lower Young's modulus and superior elasticity and elastic recovery (Fig. 5b). The asymmetric, semi-rigid FDCA moiety favored recoiling and chain entanglement over lateral packing, thereby promoting elastic recovery, whereas the symmetric, planar TPA moiety stabilized lateral packing of aligned chains and led to permanent deformation.^126^ Importantly, the asymmetric FDCA-derived units lowered the activation energy for segmental motion, allowing PBAF50, with a furanoate fraction of 50 mol%, to adopt chain conformations favorable for coordination with *Thermomyces lanuginosus* lipase (TLL), thereby facilitating the initial hydrolysis of ester bonds adjacent to the furan rings and triggering successive chain hydrolysis in phosphate buffer (pH 6.5) at 50 °C (Fig. 5c). They subsequently prepared FDCA-based heat-shrinkable films for biodegradable packaging applications, further broadening the practical utility of this polymer family.^127,128^ In 2024, Gupta and co-workers confirmed the advantages of replacing TPA with FDCA in fully bio-based aliphatic–aromatic polyesters, which increased segmental mobility and chain entanglement, enabled large tensile deformation and excellent elastic recovery, and promoted *Candida rugosa* lipase accessibility and ester-bond hydrolysis.^129^

FDCA can be directly polycondensed with diols at high temperature to yield PET-like plastics, whereas dimethyl 2,5-furandicarboxylate (DMFD) is more commonly used because of the limited thermal stability of FDCA. Wang and co-workers prepared fully bio-based poly(pentamethylene 2,5-furanoate) (PPeF) homopolymers with different number-average molecular weight (*M*_n_) *via* polycondensation of DMFD and 1,5-pentanediol.^130^ With increasing crystallization (annealing) time, these materials gradually transformed from amorphous elastomeric states to stiff semicrystalline plastics. Increasing *M*_n_ slowed crystallization but improved overall properties, and PPeF with *M*_n_ ≈ 42 kDa showed the best balance of performance, including a tensile strength of 42.1 MPa, Young's modulus of 1025 MPa, fracture strain of 23%, and gas barrier properties comparable to PET. *M*_n_ also governed the degradation with *Candida antarctica* lipase B (CALB) in phosphate buffer (pH 7.4) at 37 °C. Although overall mass loss decreased with increasing *M*_n_, GPC revealed the largest *M*_n_ reduction for the intermediate-*M*_n_ sample (42 kDa), likely reflecting fragmentation of the lower-*M*_n_ polymer during degradation, highlighting that mass loss alone may not accurately reflect chain scission. Subsequently, they prepared poly(ethylene-diethylene glycol 2,5-furandicarboxylate) (PEDF) copolyesters, all of which were amorphous and formed transparent films.^131^ Increasing the diethylene glycol (DEG) content enhanced chain flexibility and toughness, and PEDF containing 40% DEG exhibited a 10.7-fold higher fracture strain (43%) while retaining a high Young's modulus of 2440 MPa and tensile strength of 74 MPa, thereby surpassing commercial bottle-grade PET in both mechanical and gas barrier properties.^132^ Importantly, higher DEG contents also increased mass loss with CALB in phosphate buffer (pH 8.0) at 37 °C, likely because the ether linkages increased hydrophilicity and chain flexibility, making the ester bonds more accessible to enzymatic hydrolysis.

FDCA can also be incorporated as a partial component into biodegradable plastics to tune both properties and degradability. Wei and co-workers introduced FDCA in poly(butylene oxalate) (PBOx) to prepare random poly(butylene oxalate-*co*-furanoate) (PBOF).^56^ At low-to-moderate butylene furanoate (BF) contents, the crystalline structure of PBOx was disrupted, whereas high BF contents (≥69 mol%) promoted poly(butylene furanoate) (PBF)-like crystallization and significantly increased tensile strength and modulus, while affording barrier properties superior to PBAT. Enzymatic degradation with CALB in phosphate buffer (pH 7.4) at 37 °C was more pronounced than degradation in fresh water, seawater, or soil, although the mass loss progressively decreased with increasing BF content. The steric hindrance and asymmetry of the furan units, which restricted chain mobility and water penetration and increased the hydrolysis energy barrier of oxalate units. Zhu and co-workers have introduced FDCA into PCL,^133^ PNF,^134^ and further prepared biobased PBF copolymers with lactic acid units (PBLF) *via* melt polycondensation of ethyl lactate-succinate (SLA, as shown in Tables 1 and 3), DMFD, and BDO.^135^ At SLA contents ≥30%, the melting peak of PBF disappeared, converting PBLF from a rigid plastic into an amorphous elastomeric material with improved chain flexibility, which further increased mass loss with CALB in phosphate buffer at 37 °C. The degradability of PBLF correlated with the average aromatic sequence length, and obvious degradation occurred when this value fell below 3. Most recently, Wang and co-workers found that moderate FDCA incorporation (∼15 mol%) into PBS yielded PBSF copolymers with markedly mechanical improvement, with tensile strength and fracture strain increasing by 30.63% and 467.59%, respectively.^136^ The rigid furan rings enhanced chain stiffness and cohesive intermolecular interactions while partially disrupting crystalline packing and increasing the amorphous fraction, thereby improving energy dissipation. FDCA incorporation can enhance surface hydrophilicity, amorphous fraction, and ester-bond accessibility, thereby increasing mass loss with CALB in phosphate buffer (pH 7.4) at 37 °C. Optimized PBSF showed good cytocompatibility and significantly upregulated osteogenic markers *in vitro*, highlighting its promise for sustainable bone-repair scaffolds.

#### 2.2.2 Isohexides

Isohexide-derived bicyclic diols, including isosorbide (IS), isomannide (IM), and isoidide (II), have emerged as renewable feedstocks for sustainable polymer design.^116,119,137^ Their rigid, oxygen-containing, and relatively hydrophilic structures make them useful for improving stiffness, thermal properties, and enzyme/water accessibility in diverse plastic backbones (Table 2), with IS being the most extensively studied.^116,137,138^

**Table 2:** Effects of structural modification on the properties and enzymatic degradability of isohexide-based polymers

Polymers	Effects on physical properties	Effects on enzymatic degradation	Ref.
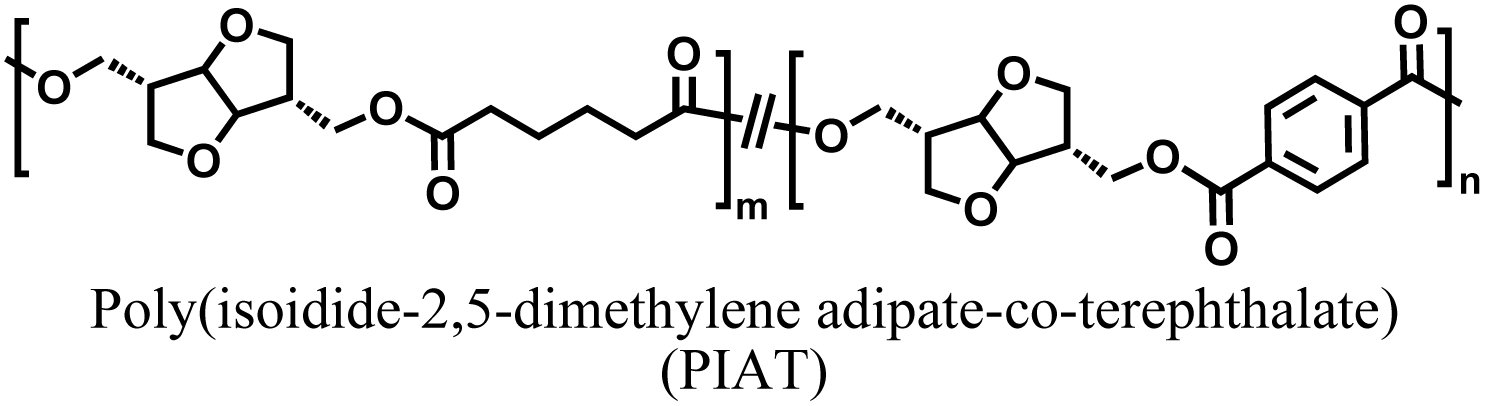	Increasing dimethyl terephthalate (DMT) content enhanced *T*_g_, *T*_m_, hydrophobicity and storage modulus at higher DMT contents, but reduced molecular weight and crystallinity	Increasing DMT content slowed enzymatic degradation with porcine pancreatic lipase in phosphate buffer (pH 7.2–7.4) at 37 °C after 8 weeks, but PIATs still degraded faster than PBAT, which showed only ∼5% mass loss	139
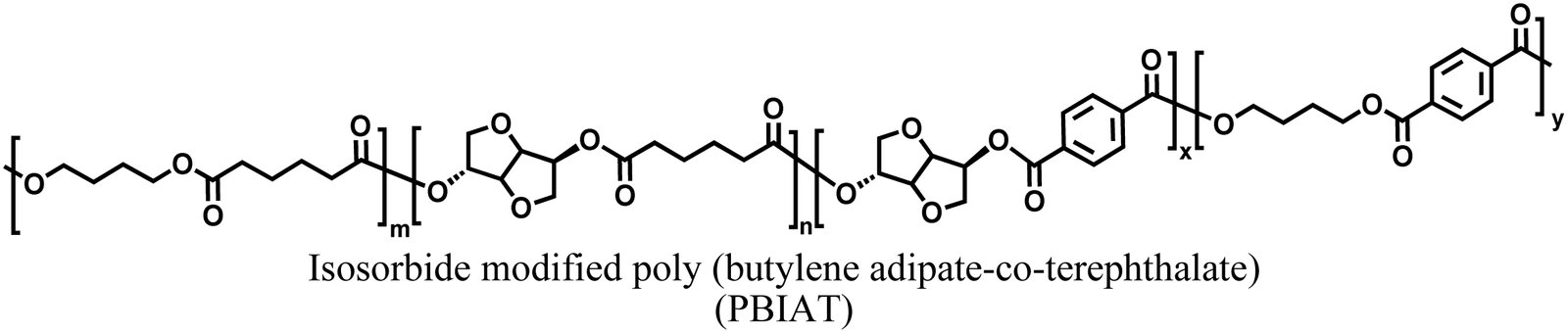	Increasing isosorbide (IS) content enhanced *T*_g_, *T*_α_, and hydrophilicity while maintaining high thermal stability, but reduced *T*_m_, crystallinity, fracture stress, and orientation degree	IS incorporation improved enzymatic degradation, reducing residual *M*_n_ to ∼55–65% with lipase in phosphate buffer (pH 7.2–7.4) at 40 °C after 14 weeks	140
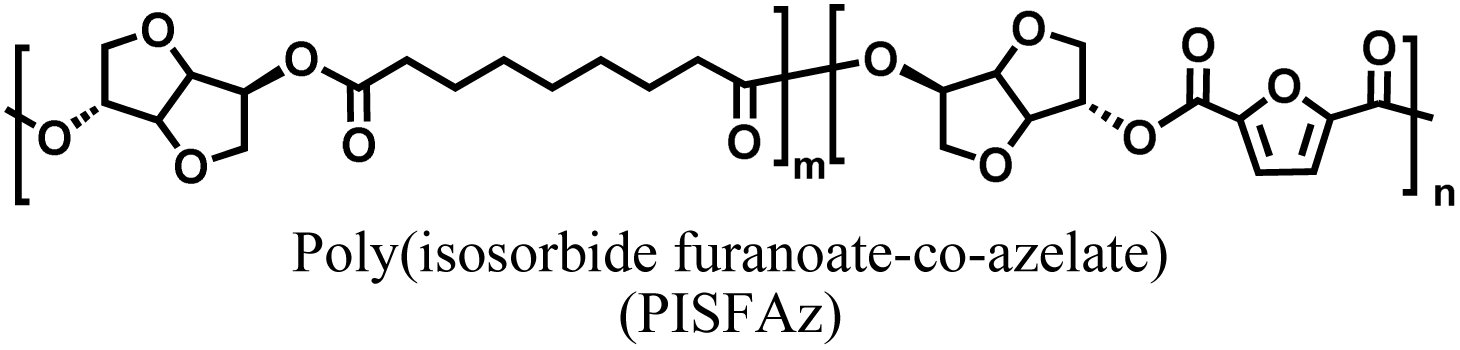	IS disrupted chain regularity and afforded amorphous structures. Increasing FDCA content raised *T*_g_ and char residue but reduced sequence randomness at high FDCA contents	Copolymers showed composition-dependent enzymatic hydrolysis, with maximum mass loss of ∼61% after 30 days using lipases from *Pseudomonas cepacia* and *Rhizopus oryzae* in phosphate buffer (pH 7.4) at 36.6 °C	141
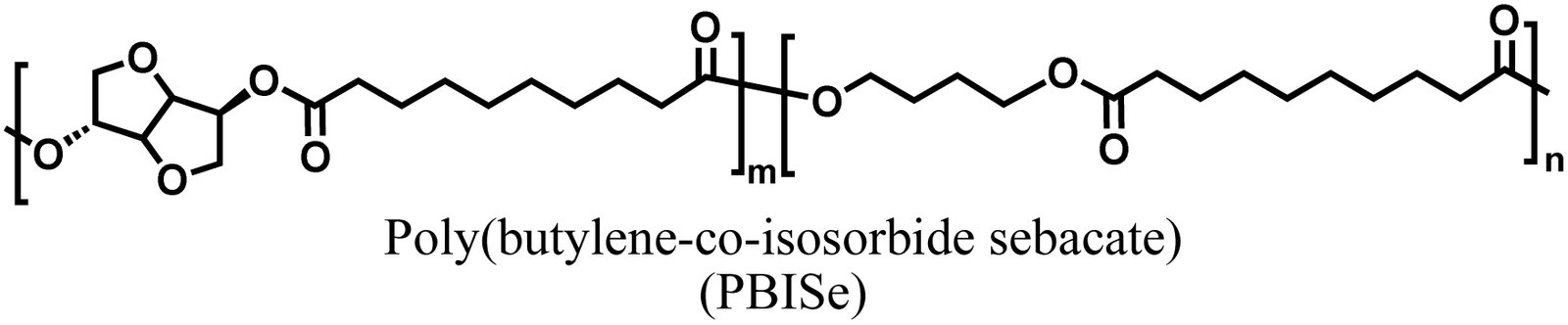	Increasing IS content enhanced *T*_g_ and hydrophilicity, but reduced *T*_m_ and crystallinity, and molecular weight at high IS contents	Increasing IS content improved enzymatic degradation, raising mass loss from 12% to 92% with *Pseudomonas cepacia* lipase in phosphate buffer (pH 7.4) at 37 °C after 8 weeks	142
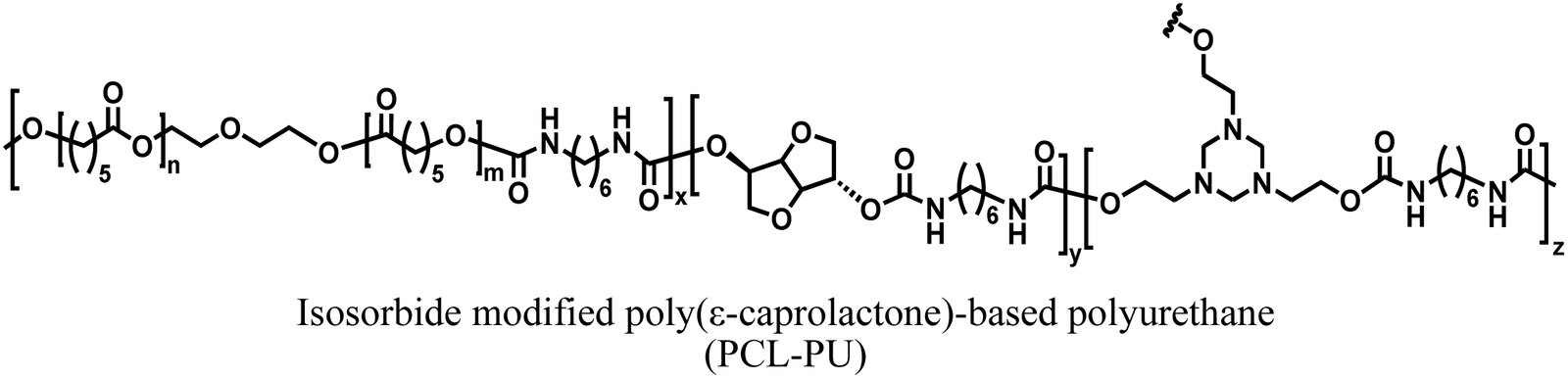	Increasing hexahydrotriazine (HT) content enhanced *T*_m_ and hydrophilicity, whereas high HT contents compromised tensile strength and fracture strain	Increasing HT content improved enzymatic degradation, raising mass loss from 2.7% to 28.7% with lipase in phosphate buffer at 37 °C after 6 weeks	143
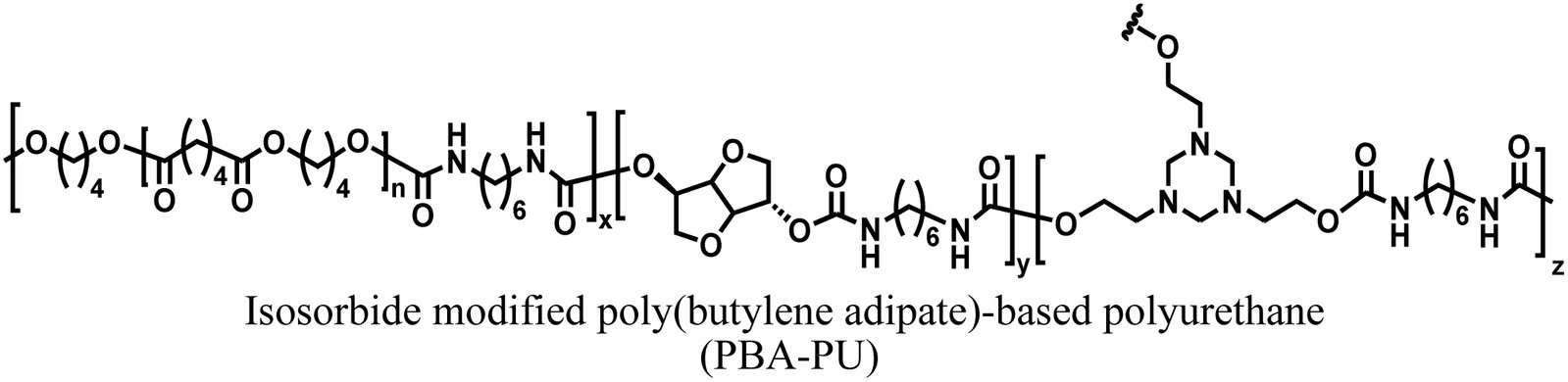	Increasing PBA segment molecular weight enhanced *T*_d5%_, soft-segment crystallinity, fracture strain and shape-memory performance, but reduced ordered hydrogen bonding	Increasing PBA segment molecular weight improved enzymatic degradation, raising mass loss from 10.8% to 50.0% with *Pseudomonas cepacia* lipase in phosphate buffer (pH 7.4) at 37 °C after 6 weeks	144

Wang and co-workers introduced II-derived isoidide-2,5-dimethanol (IIDML) into PBAT-like copolyesters (PIAT) through polycondensation with dimethyl adipate (DMA) and dimethyl terephthalate (DMT) (Fig. 5d).^139^ Because the PIT homopolyester exhibited an extremely high melting temperature and insufficient thermal stability, chain growth of IT segments was limited and occurred preferentially through IIDML-DMA-rich segments, leading to a highly blocky microstructure (Fig. 5e). This unique sequence distribution endowed PIATs with thermal properties superior to commercial PBAT (Fig. 5f), while the rigid IIDML and DMT units synergistically increased storage modulus and rigidity. Although enzymatic mass loss decreased with increasing DMT content, the hydrophilic IIDML-derived units likely facilitated water diffusion and surface hydrolysis, enabling PIATs to exhibit greater mass loss than PBAT with porcine pancreatic lipase in phosphate buffer (pH 7.2–7.4) at 37 °C (Fig. 5g). They later prepared IS-modified PBAT tetrapolyesters (PBIAT), in which the low reactivity and rigid, kinked structure of IS promoted partial blocky sequence formation, especially around IS-based repeating units.^140^ This microstructure, together with the rigid IS units, enhanced elastic response, chain entanglement, segmental rigidity, and relaxation time, thereby modifying melt rheology for monofilament-fiber fabrication. Notably, IS improved hydrophilicity and reduced crystalline regularity, leading to faster *M*_n_ reduction and more pronounced surface erosion than PBAT with lipase in phosphate buffer (pH 7.2–7.4) at 40 °C.

Bikiaris and co-workers prepared fully biobased poly(isosorbide furanoate-*co*-azelate) (PISFAz) copolymers with IS, FDCA, and azelaic acid (Az).^141^ All copolyesters were amorphous as the rigid and asymmetric IS units disrupted chain regularity. Increasing the stiff FDCA content reduced chain mobility and increased *T*_g_ from 9.2 to 91.1 °C, while lower Az contents led to a more blocky sequence tendency. These structural changes strongly influenced lipase-catalyzed degradation in phosphate buffer (pH 7.4) at 36.6 °C. Degradation mainly proceeded through hydrolysis of ester bonds adjacent to Az units, favored by hydrophilicity and random sequence distribution, whereas ester bonds neighboring FDCA units were more resistant to enzymatic attacks because of steric hindrance. Park and co-workers introduced IS into poly(butylene sebacate) copolymers (PBISe), increasing chain rigidity and *T*_g_ while decreasing crystallinity and improving hydrophilicity.^142^ These changes enhanced enzyme–substrate interactions and accelerated ester-bond cleavage. After 8 weeks in phosphate buffer (pH 7.4) at 37 °C with *Pseudomonas cepacia* lipase, neat PBSe showed only 12% mass loss, whereas the PBI30Se containing 30 mol% IS exhibited more than 92% mass loss, with similar trends observed in soil and marine environments.

Du and co-workers successfully synthesized PCL-based PU (PCL-PU) crosslinking films containing IS and acid-labile hexahydrotriazine (HT).^143^ The rigid IS units contributed to thermal stability, while an intermediate HT content (ISO/PCL-diol/HT = 2/2/2) maximized the fraction of ordered hydrogen bonding carbonyl groups to 40.15%, corresponding to urethane carbonyl groups located in more regularly packed hard-segment domains. At this composition, PCL-PU3 exhibited the highest tensile strength of 31.1 MPa while retaining fracture strain of 736%, whereas excessive HT increased the crosslinking density, restricted chain mobility, and reduced tensile performance. The materials exhibited dual acid- and enzyme-triggered degradation, in which increasing HT content enhanced mass loss during enzymatic degradation from 2.7% to 28.7% through an autocatalytic effect, while enzyme-acid synergy shortened the overall degradation time by approximately half. The same IS/HT synergistic strategy was later extended to poly(butylene adipate) (PBA)-based PUs, where variation of the PBA soft-segment molecular weight enabled simultaneous regulation of mechanical performance and enzymatic degradability.^144^ An intermediate PBA molecular weight of 2000 g mol^−1^ provided the optimal balance between chain flexibility, hard/soft segment interactions and crystallinity, affording the highest tensile strength of 25.7 MPa and a fracture strain of 860%. In contrast, enzymatic degradation by *Pseudomonas cepacia* lipase increased progressively with PBA molecular weight, reaching 50% mass loss after 6 weeks for the 3000 g mol^−1^ soft segment, owing to its higher content of hydrolyzable ester bonds. However, acid hydrolysis of HT-containing plastics is expected to release formaldehyde, which may interfere with enzyme activity in coupled degradation processes and raises concerns regarding the toxicity of the degradation products, potentially limiting the overall sustainability of this acid-triggered pathway.

#### 2.2.3 Bio-derived long-chain feedstocks

Long-chain feedstocks derived from plant oils offer a promising green route to biodegradable PE-like plastics by combining hydrophobicity, crystallinity, thermal stability, and mechanical robustness of PE with hydrolyzable ester linkages that enable biodegradability.^120,145^ These materials have therefore attracted extensive interest as sustainable alternatives to conventional PE and other plastics.^120,145–147^

**Fig. 6:**
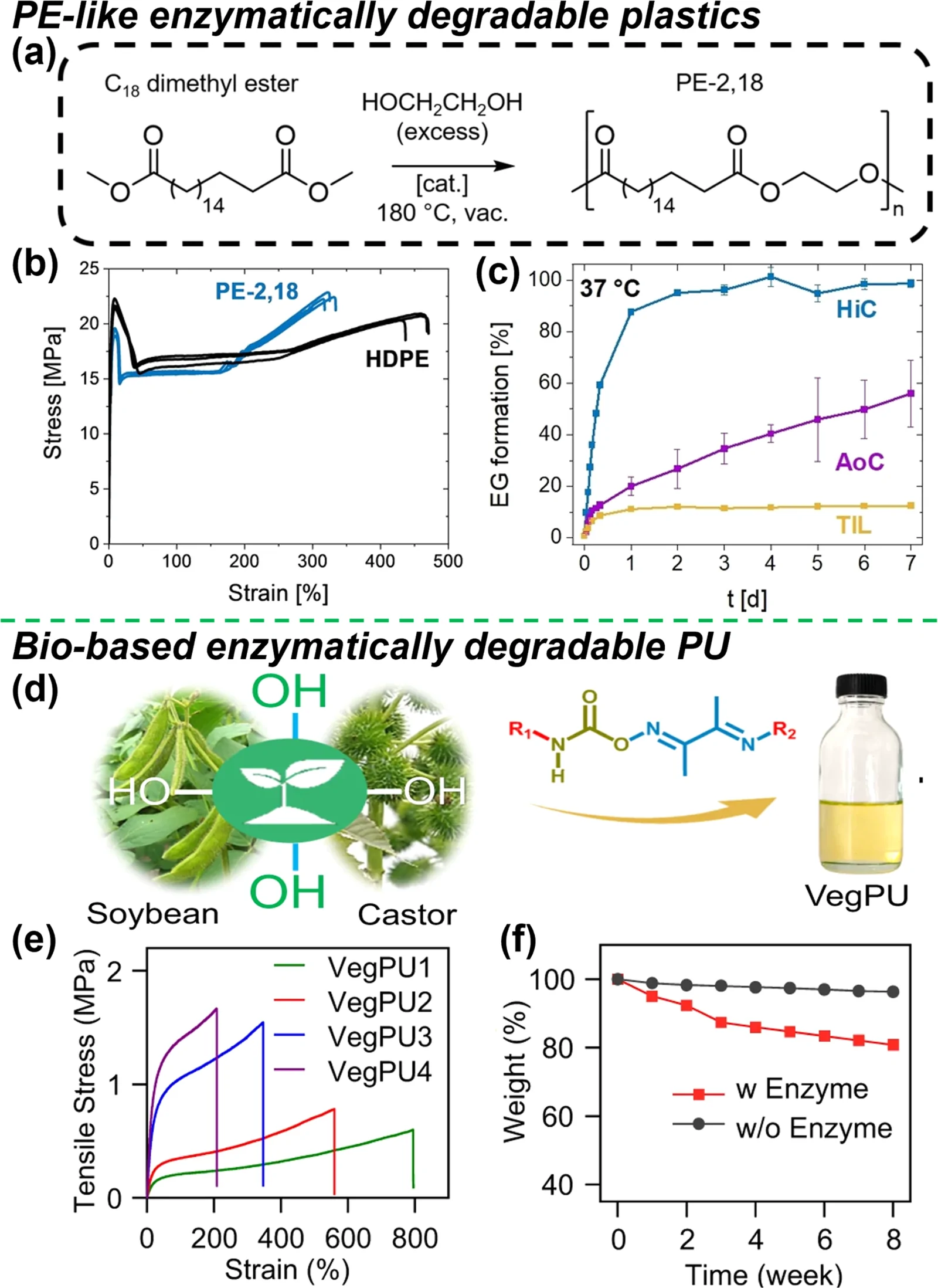
(a) Polymerization, (b) mechanical properties, and (c) enzymatic degradation of PE-like enzymatically degradable plastics. (Reproduced from ref. 150 under a CC BY-NC-ND 4.0 license). (d) Preparation, (e) mechanical properties, and (f) enzymatic degradation of bio-based enzymatically degradable PU. (Reproduced from ref. 153 under a CC BY 4.0 license).

Mecking and co-workers have conducted extensive pioneering work on PE-like polyesters, significantly advancing the development of this class of materials.^74,145,148–152^ For example (Fig. 6a), they reported a novel PE-like polyester-2,18 (PE-2,18) prepared through polycondensation of renewable 1,18-dimethyl octadecanedioate and ethylene glycol (EG).^150^ The resulting polymer combined a high *T*_m_ (96 °C), strong hydrophobicity, high crystallinity, and mechanical properties comparable to those of HDPE (Fig. 6b). Notably, PE-2,18 was hydrolyzed by several naturally occurring polyester hydrolases in phosphate buffer (pH 7.2), among which *Humicola insolens* cutinase (HiC) exhibited the highest activity, likely owing in part to its greater active-site accessibility. HiC achieved complete depolymerization to the constituent EG and C18 diacid monomers within approximately 3–4 days at 37 °C, while more than 60% monomer formation was observed after one week at 25 °C (Fig. 6c). By contrast, PE-18,18, despite similar crystallinity and melting temperature, was far less degradable, presumably because the poorly water-soluble C18 diol products released during hydrolysis remained on the polymer surface, thereby forming a protective layer that hindered further enzymatic hydrolysis.^74^ These PE-like materials could also be melt-spun at relatively low temperatures (120 °C),^151^ yielding fibers with fracture stress up to ∼270 MPa, compared favorably to PET fibers spun at a similar rate, while retaining similar enzymatic degradability trends compared to the corresponding films. Notably, PE-2,18 multifilaments were processed into knitted fabrics and cotton-blended yarns, while fibers and fabrics remained intact even after 10 washing cycles. They further expanded this platform by combining renewable 2,3-butanediol (2,3-BD) with long-chain dicarboxylic acids or diesters (C10–C48).^152^ With increasing dicarboxylate chain length, the materials evolved from liquids to white solids at room temperature, accompanied by increased *T*_m_ and crystallinity, as longer alkyl segments progressively diminished the crystallization-disrupting effect of methyl branches. PE-b4.18, prepared from 2,3-BD and dimethyl 1,18-octadecanedioate (C18 diester), exhibited an LDPE-like mechanical profile and a fourfold higher HiC-catalyzed ester-bond hydrolysis rate than its linear counterpart PE-4.18 (prepared from 1,4-butanediol and C18 diester) in phosphate buffer (pH 7.0) at 30 °C, owing to lower crystallinity induced by methyl branches.

Zhu and co-workers developed PE-like copolyesters (PBTDP, structure shown in Table 3) from bio-based *N*,*N*′-dimethylene-bis(pyrrolidone-4-carboxylic acid) (EBPCA), 1,14-tetradecanedioic acid (TDA), and 1,4-butanediol.^72^ The rigid pyrrolidone ring disrupted the ordered arrangement of methylene chains, reducing crystallinity and lamellar thickness while enhancing chain mobility, thereby moderately decreasing rigidity but significantly improving ductility and toughness. All PBTDP exhibited fracture strain above 1000% and tensile strength above 22.9 MPa, overperforming other reported PE-like polyesters, commercial LLDPE and HDPE. Importantly, pyrrolidone units acted as both hydrophilic and enzyme-binding sites, lowering the hydrolysis activation energy by 7.1 kcal mol^−1^ and increasing population of pre-reaction-state conformations in CALB from 0 to 23.2% through noncovalent interactions, thereby accelerating degradation as the EBPCA contents increased. This material can be selectively hydrolyzed by enzymes in mixed plastic waste to recover TDA in 92% yield. The recovered TDA was then repolymerized *via* melt polycondensation into a regenerated material with even better mechanical performance, exhibiting a tensile strength of 30.1 MPa and a fracture strain of 1382%, due to its higher molecular weight.

**Table 3:** Effects of structural modification on the properties and enzymatic degradability of copolymers with other feedstocks

Origins	Feedstocks	Polymers	Effects on physical properties	Effects on enzymatic degradation	Ref.
Bio-based	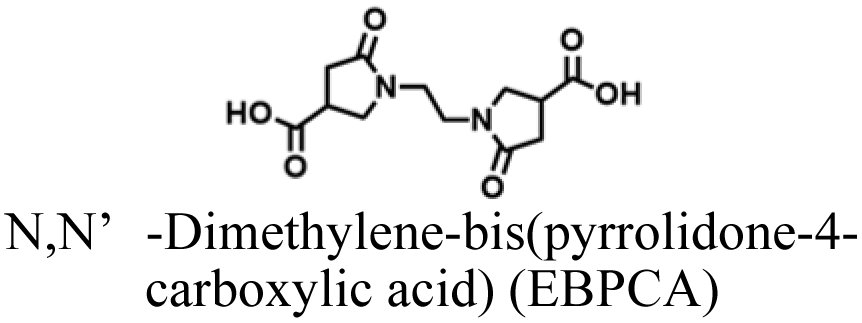	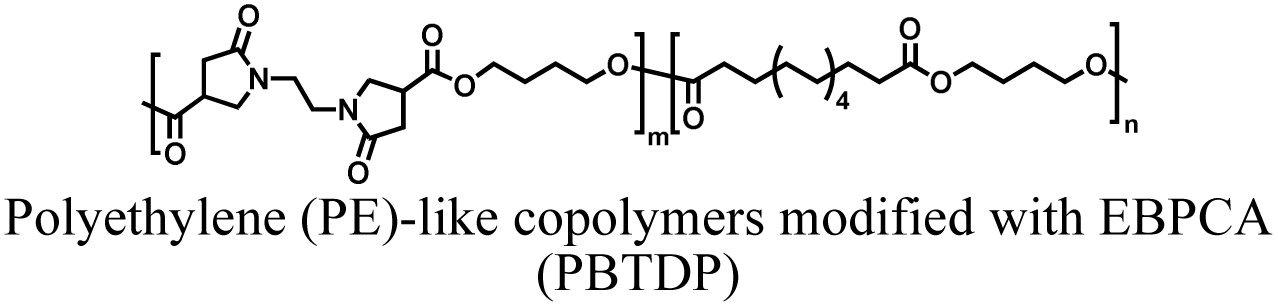	Increasing EBPCA content reduced crystallinity, *T*_m_, stiffness, and hydrophobicity, but maintained high stretchability (>1000%) and thermal stability	Increasing EBPCA content improved enzymatic degradation, raising mass loss from 10–15% to ∼100% with CALB in phosphate buffer (pH 7.4) at 37 °C after 10 days	72
	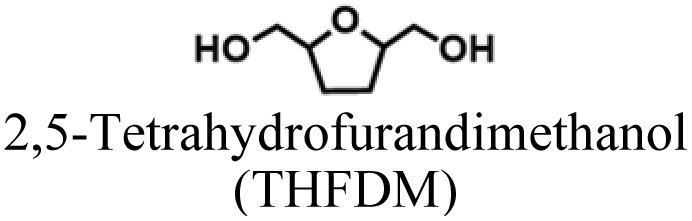	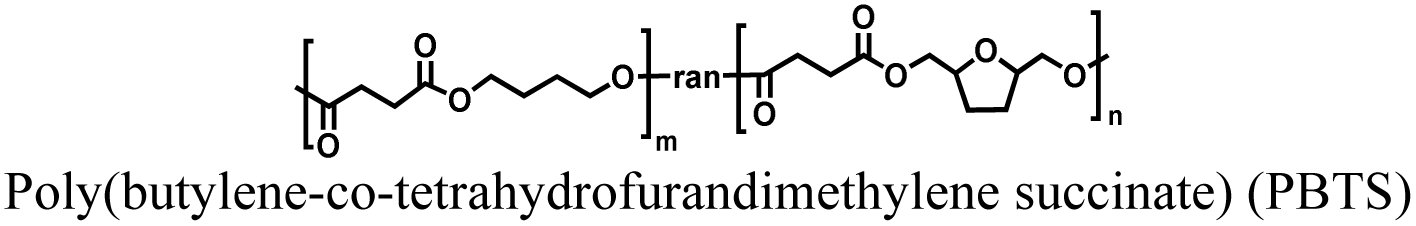	Increasing THFDM content enhanced hydrophilicity, stretchability and toughness, but reduced crystallinity, stiffness, and gas barrier properties slightly	Increasing THFDM content improved enzymatic degradation, raising mass loss from <10% to ∼60% with lipase in phosphate buffer (pH 7.2) at 30 °C after 70 days	157
	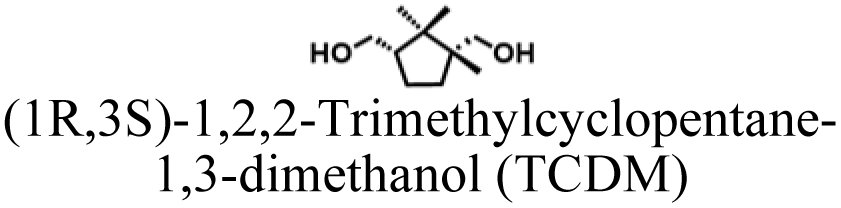	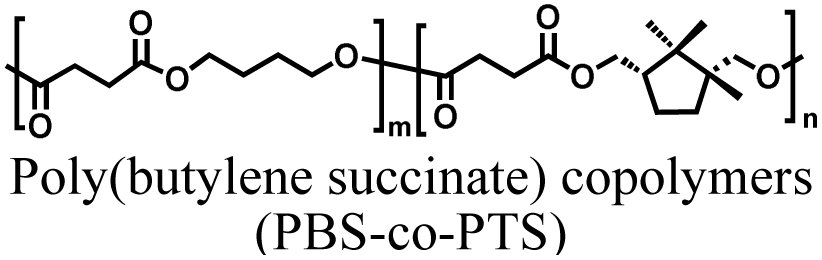	TCDM incorporation increased the *T*_g_, thermal stability, and hydrophobicity, but reduced crystallinity and mechanical strength, with more pronounced effects in random copolymerization (RC)-prepared copolyesters	TCDM incorporation improved enzymatic degradation, raising mass loss from negligible to ∼18% (RC) with porcine pancreas lipase in phosphate buffer (pH 7.4) at 37 °C after 6 weeks	161
	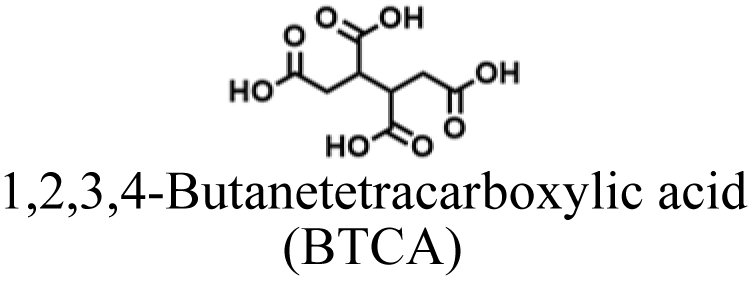	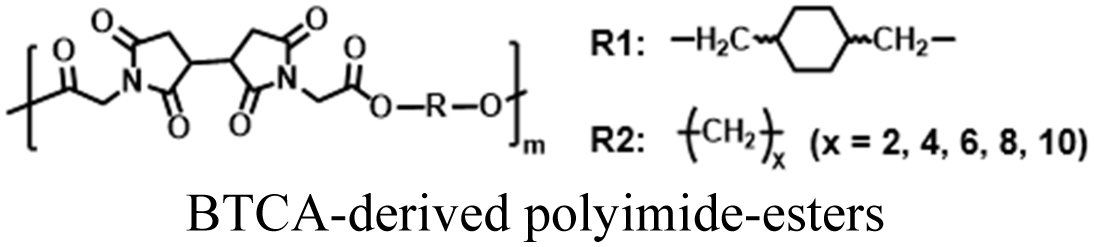	Longer α,ω-diol chains enhanced stretchability while maintaining excellent thermal stability and high tensile strength, but reduced *T*_g_ and hydrophilicity	Longer α,ω-diol chains suppressed enzymatic degradation, and PEB showed the highest mass loss of ∼30% with lipase in phosphate buffer at 25 °C after 10 weeks	164
Petroleum-based	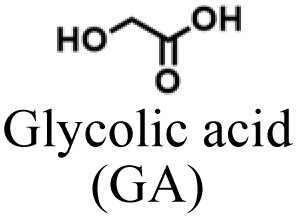	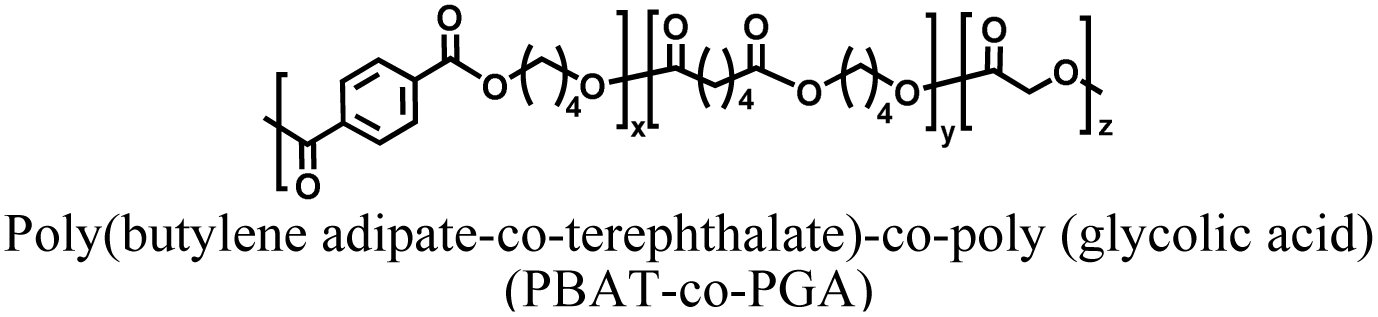	GA incorporation enhanced *T*_g_, crystallinity, modulus, and water vapor barrier properties, but slightly reduced thermal stability while maintaining high ductility and toughness	GA incorporation improved enzymatic degradation, raising mass loss from 4.7% to 8.6% with *Candida rugosa* lipase in phosphate buffer (pH 7.4) at 37 °C after 13 weeks	168
	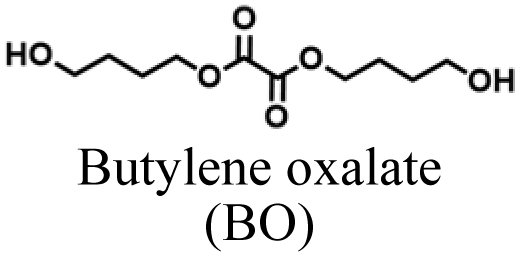	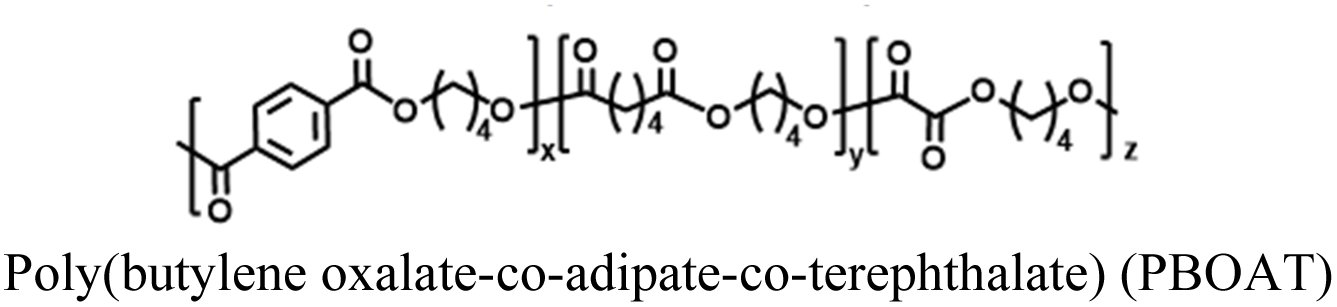	Increasing BO content enhanced *T*_g_, Young's modulus, hydrophilicity, and gas barrier properties, but reduced thermal stability and fracture strain at high BO contents	Increasing BO content improved enzymatic degradation, raising mass loss from 20% to 64% with CALB in phosphate buffer (pH 7.4) at 37 °C after 56 days	169
	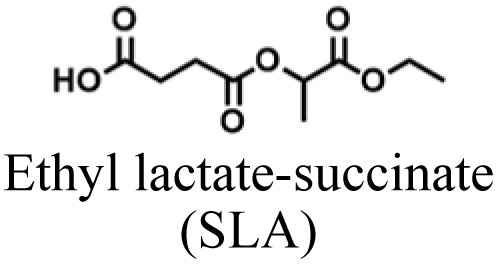	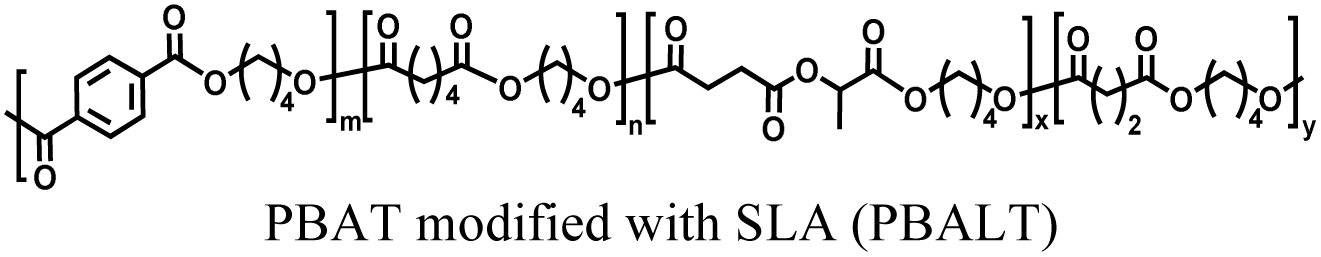	Increasing SLA content enhanced *T*_g_ and gas barrier performance, but reduced *T*_m_, crystallinity, modulus, and tensile strength while maintaining high stretchability (>1100%)	Increasing SLA content improved enzymatic degradation, raising mass loss from ∼2.0% to 16.3% and *M*_n_ loss from ∼14% to ∼64% with CALB in phosphate buffer (pH 7.4) at 37 °C after 49 days	170
		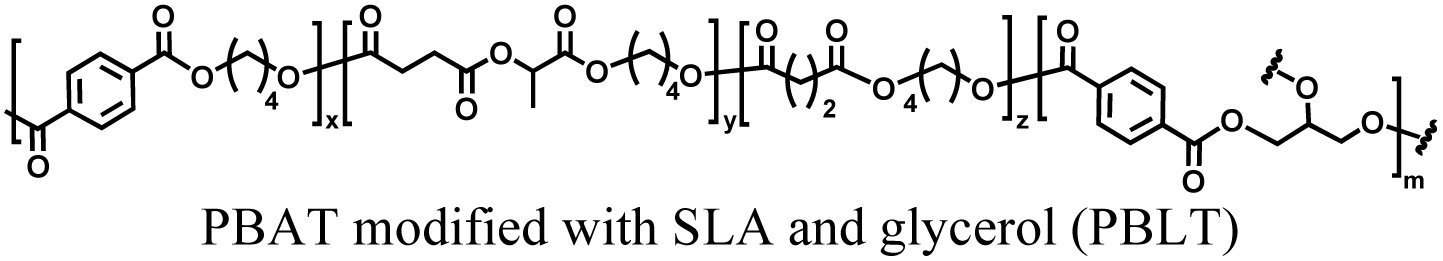	Increasing SLA content reduced *T*_g_ and enabled a transition from plastic to elastomer. Micro-crosslinks greatly enhanced stiffness and strength while retaining high elongation (>1400%), without compromising thermal stability or thermoplastic processability	Increasing SLA content improved enzymatic degradation, whereas micro-crosslinks slightly suppressed degradation, with all samples showing 35–50% mass loss with CALB in phosphate buffer (pH 7.4) at 37 °C after 5 weeks	171
	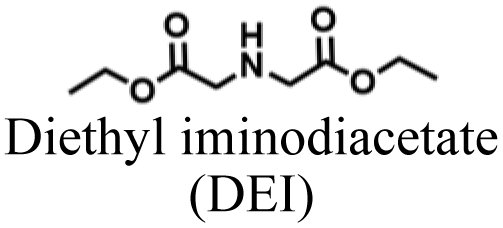	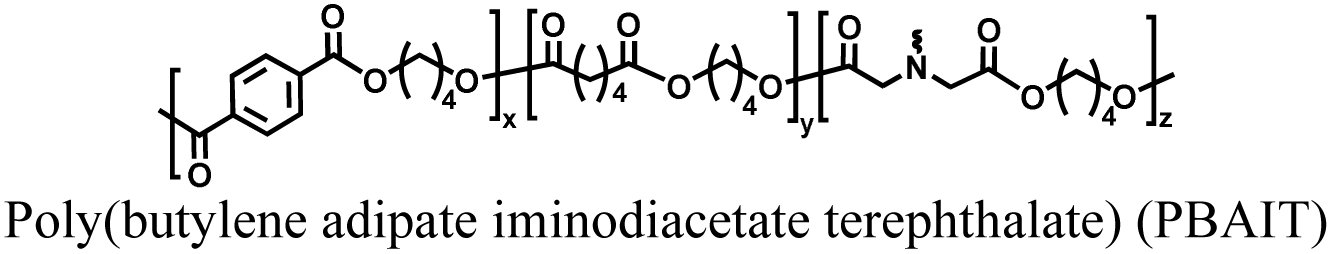	DEI incorporation enhanced *T*_c_, *T*_g_, *T*_m_, crystallinity, modulus, and hydrophilicity, but reduced fracture strain. Tensile strength was optimized at 10 mol% DEI (23.87 MPa)	Increasing DEI content improved enzymatic degradation, raising mass loss from <4% (PBAT) to 16% with CALB in phosphate buffer (pH 7.2–7.4) at 60 °C after 49 days	172
	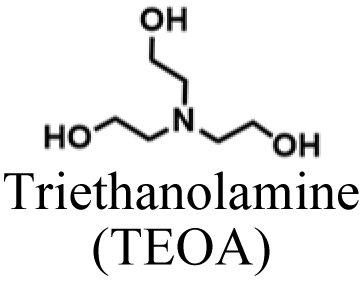	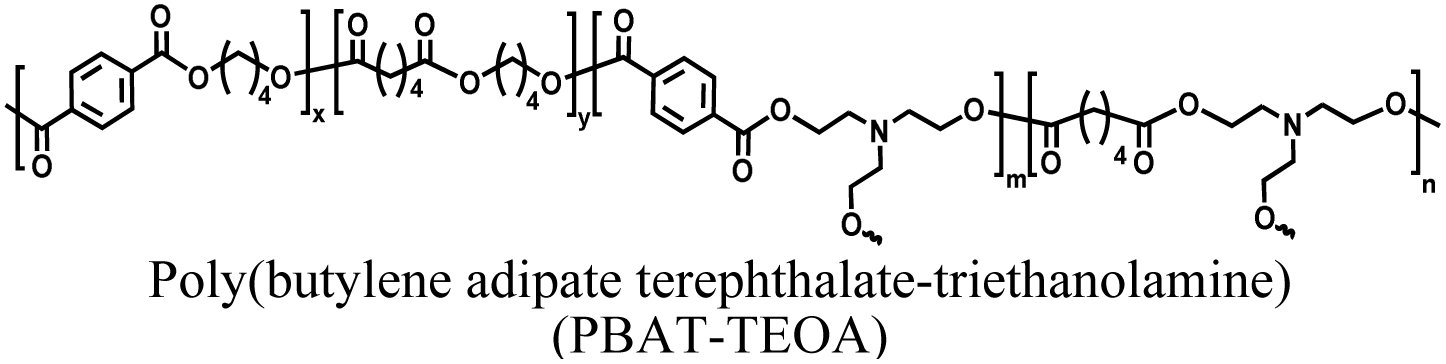	TEOA incorporation enhanced *T*_c_ and crystallinity at low contents, improved Young's modulus and hydrophilicity, but reduced *T*_g_, *T*_m_, and stretchability. Tensile strength was optimized at 0.5% (20.96 MPa)	TEOA incorporation improved enzymatic degradation, raising mass loss from ∼10% (PBAT) to ∼30% with CALB in phosphate buffer (pH 7.4) at 60 °C after 49 days	173

Beyond PE-like plastics, bio-based long-chain polyols have also been developed as soft segments for bio-based polyurethanes (PUs).^147^ Lee and co-workers prepared vegetable oil-based PUs (VegPU) from castor oil- and epoxidized soybean oil-derived polyols (Fig. 6d).^153^ The resulting VegPU combined excellent elasticity, cyclic self-recovery, high extensibility, and notch tolerance, attributed to dynamic covalent bonds and multiple hydrogen-bonding interactions (Fig. 6e). In addition to excellent reprocessability *via* solution casting or hot-pressing, VegPU can be degraded by *Candida rugosa* lipase with 19.2% mass loss after 8 weeks (Fig. 6f). Hakkarainen and co-workers reported bio-based PU thermosets derived from branched polyester polyols with tunable aliphatic chain lengths.^154^ By tuning the crosslink density and aliphatic chain length, these PUs exhibited broadly tunable thermal properties and mechanical performance comparable or superior to those of most reported bio-based PUs. Although thermosetting, these PUs showed tunable enzymatic mass loss in phosphate buffer (pH 7.4) at 37 °C containing *Candida rugosa* lipase and *Aspergillus niger* lipase. Free hydroxyl groups and lower crosslink density in the partial crosslinks increased hydrophilicity and water uptake, facilitating enzyme access to ester bonds, while shorter diacid and triol segments further accelerated degradation by increasing ester/hydroxyl group density and hydrolysable motifs.^155^ Recently, Zhang and co-workers prepared an amorphous bio-based polyester polyol from renewable feedstocks and used it as a soft segment to construct various PUs with different diisocyanates.^156^ Among them, hexamethylene diisocyanate (HDI)-based PU exhibited the highest microphase separation which enhanced both mechanical properties and enzymatic degradability. Increasing the HDI-based hard-segment content further enhanced stiffness but weakened enzymatic degradation. Accordingly, the PU with lowest HDI content (HDI-17%) lost 82.0% mass with CALB in buffer (pH 7.4) at 37 °C after 20 days and 89.1% mass under composting conditions after 90 days. The degradation products were primarily derived from enzymatic cleavage of the polyester soft segments, and the resulting degradation solution showed no apparent cytotoxicity, although their distribution, yield, and potential for recovery were not further investigated. HDI-17% also exhibited cold resistance, biocompatibility, and could be fabricated into customized orthopedic devices, showing potential for biodegradable medical implants.

#### 2.2.4 Other bio-based feedstocks

Bio-based 2,5-tetrahydrofurandimethanol (THFDM), a cyclic monomer with a synthetic route similar to that of FDCA, has recently emerged as an eco-friendly alternative to petroleum-derived 1,4-cyclohexanedimethanol for preparing various biodegradable polymers.^157–159^ Wei and co-workers introduced THFDM into PBS to obtain poly(butylene-*co*-tetrahydrofurandimethylene succinate) (PBTS).^157^ The rigid THFDM ring increased segmental rigidity and *T*_g_ but reduced crystallinity and stiffness, thereby enhancing the stretchability from 494.5% to 1112.5% and the toughness. Importantly, THFDM incorporation markedly increased mass loss of PBTS with lipase in phosphate buffer (pH 7.2) at 30 °C, which was attributed to the increased hydrophilicity provided by the ether groups and the reduced crystallinity resulting from disruption of chain packing by the cyclic THFDM units.

Derived from natural camphor, (1R,3S)-(+)-cis-1,2,2-trimethylcyclopentane-1,3-dimethanol (TCDM) has also been explored as a renewable feedstock for polymerization.^160–162^ Gao and co-workers incorporated TCDM into PBS either by random copolymerization (RC) or reactive blending (RB) to obtain random and multiblock copolymers, respectively.^161^ In both series, TCDM incorporation significantly increased *T*_g_ and reduced crystallization, whereas its mechanical effects differed markedly. RC copolyesters showed pronounced strength loss and limited ductility, whereas RB copolyesters retained satisfactory strength and exhibited improved stretchability from ∼14% for PBS to ∼250%. This was attributed to the preservation of longer PBS sequences, which favored crystallization and reinforcing crystalline domains. Although TCDM increased hydrophobicity, the copolymers degraded faster than PBS in phosphate buffer (pH 7.4) at 37 °C with porcine pancreas lipase, likely because this lipase is more active on hydrophobic domains and the reduced crystallinity.^163^ After six weeks, the RC copolyesters exhibited 13–18% mass loss, compared with ≤11% for their RB counterparts, mainly owing to their lower crystallinity, although differences in initial molecular weight may also have contributed.

Recently, Pang and co-workers prepared a bio-based biimide diester (BD) from 1,2,3,4-butanetetracarboxylic acid (BTCA) and polycondensed it with various diols to afford a series of amorphous polyimide-esters.^164^ The Z-shaped BD structure increased interchain free volume and suppressed crystallization, while polar interactions between imide rings restricted segmental mobility, imparting high *T*_g_ up to 136 °C, high thermal stability, and high rigidity, with the best copolymer reaching a Young's modulus of 1512 MPa and a tensile strength of 51.6 MPa, comparable to those of conventional PET and PBT. Their toughness was tunable by diol structure, with longer linear diols promoting yielding and ductile fracture. Moreover, shorter diol segments increased hydrophilicity, while the amorphous morphology and neighboring-group participation of BD imide nitrogen atoms facilitated ester-bond cleavage, leading to greater mass loss in lipase-containing buffer than in artificial seawater or deionized water.

Compared with the direct utilization of biopolymers, bio-based feedstocks offer substantially greater molecular design flexibility, enabling the construction of plastics with diverse chemical structures and tailored functionalities. Through rational molecular design, these materials can achieve mechanical and thermal performances comparable to, or even exceeding, those of many conventional petroleum-derived plastics while maintaining or even enhancing enzymatic degradability. Nevertheless, current studies have largely focused on demonstrating enzymatic degradation, whereas the identification, recovery, and reutilization of the resulting degradation products remain largely unexplored. Addressing these aspects will further unlock the potential of bio-based feedstocks as sustainable resources for high-performance plastics and accelerate the transition toward a truly circular plastics economy.

### 2.3 Synthetic copolymerization

Copolymerization with appropriate monomers or chain segments is an effective strategy for combining the advantages of different components, enabling copolymers to exhibit properties unattainable in the corresponding homopolymers.^23–25,165,166^ Beyond the bio-based feedstocks discussed in Section 2.2, such as FDCA and IS, additional monomers can also be incorporated to improve both the performance and degradability of plastics (Table 3). In addition, sequence architecture, including random and sequence-controlled arrangements, can exert distinct effects on the properties of the plastics,^161^ as discussed below.

#### 2.3.1 Random copolymerization

PBAT possesses good ductility, stiffness, heat resistance, barrier performance, and processability, making it a desirable biodegradable plastic for packaging and agricultural mulching applications.^35,36^ However, its biodegradability remains limited, largely because the rigid aromatic domains are more difficult to hydrolyze and thus compromise overall degradability.^23,167^ To address this, many studies have sought to improve degradability without sacrificing performance by increasing the aliphatic content or introducing additional degradable segments through copolymerization.

Wang and co-workers introduced glycolic acid (GA) units into PBAT to achieve random PBAT-*co*-PGA copolyesters.^168^ GA incorporation shortened the aromatic sequence length and promoted more regular lamellar growth, thereby improving crystallinity. As a result, the copolyesters retained high stretchability and toughness while showing higher modulus and tensile strength than PBAT. Importantly, GA incorporation increased the susceptibility of PBAT-*co*-PGA copolyesters to hydrolysis by *Candida rugosa* lipase in phosphate buffer (pH 7.4) at 37 °C, as evidenced by greater mass loss and a substantial decrease in molecular weight, which were attributed to the shortened aromatic BT sequences and the preferential hydrolysis of GA-containing units. Zhu and co-workers also introduced readily hydrolysable butylene oxalate (BO) units into PBAT to obtain random PBOAT copolyesters.^169^ The short BO segments reduced chain flexibility and increased the *T*_g_ while preserving crystalline structure and rapid crystallization, which further enhanced gas barrier properties and Young's modulus while maintaining high stretchability. Importantly, BO incorporation markedly increased mass loss of PBOAT with CALB in phosphate buffer (pH 7.4) at 37 °C, which was attributed to the higher susceptibility of oxalate-containing units to nucleophilic attack and their lower hydrolysis energy barrier, allowing BO units to serve as hydrolysis-prone breaking points that facilitated ester-bond cleavage.

Zhu and co-workers further introduced lactyl units into PBAT by copolymerizing SLA to obtain high-molecular-weight random PBALT copolyesters.^170^ All copolymers retained high fracture strains exceeding 1100%, whereas stiffness gradually decreased with increasing SLA content, mainly because the SLA reduced crystallinity and *T*_m_. The increased amorphous content also facilitated degradation with CALB in phosphate buffer (pH 7.4) at 37 °C, as both the residual mass and *M*_n_ of PBALT decreased substantially with increasing SLA content. Meanwhile, SLA incorporation improved gas and water-vapor barrier performance by reducing chain mobility and free volume, thereby slowing gas diffusion. To compensate for the loss of stiffness caused by SLA incorporation, Wang and co-workers introduced a trace amount of glycerol (0.5–0.9 mol%) as a micro-crosslinking agent to prepare PBLT with low-density crosslinking.^171^ The resulting elastomers retained good elastic recovery, while micro-crosslinks increased the Young's modulus from ∼9.8 to 21.8 MPa and the tensile strength from 4.5 to 16.1 MPa, and even improved the fracture strain to 2010%. SLA incorporation also increased mass loss of copolymers with CALB in phosphate buffer (pH 7.4) at 37 °C, whereas the micro-crosslinking network only slightly retarded the mass-loss rate.

Recently, Wang and co-workers introduced branched structures into PBAT by incorporating diethyl iminodiacetate (DEI), which contains secondary amine groups, to afford random PBAIT copolyesters.^172^ During polymerization, 75–79% of DEI units underwent amidation with ester bonds to form branched sites, while the remaining secondary amines contributed to hydrogen bonding. Moderate DEI incorporation (≤10 mol%) promoted crystallization by acting as nucleating motifs and, together with hydrogen bonding and branched entanglement, improved thermal behaviors and stiffness while maintaining high toughness. In contrast, excessive DEI (≥15 mol%) restricted chain mobility and stretch-induced orientation, reducing elongation and toughness. DEI incorporation increased the hydrophilicity of PBAIT and resulted in greater mass loss with CALB in phosphate buffer (pH 7.2–7.4) at 60 °C. Non-amidated BI units degraded preferentially, followed by amide-bond cleavage, causing rapid molecular-weight reduction and further accelerating overall degradation. However, secondary amines with active hydrogen can cause side reactions at high temperature, causing undesirable coloration. They further employed tertiary triethanolamine (TEOA) to prepare PBAT-TEOA copolyesters, preserving the reinforcing effect of the branched architecture while avoiding the drawbacks of secondary amines.^173^ TEOA introduction improved hydrophilicity and enzyme accessibility, while its weak basicity promoted hydrolysis of ester bonds adjacent to tertiary amine sites, thereby increasing mass loss with CALB even at only 0.25 mol% TEOA. Moreover, the increased *T*_c_ enabled successful melt spinning of PBAT into fibers with a strength of 1.98 cN/dtex, providing a promising strategy for low-melting-point biodegradable fibers.

#### 2.3.2 Sequence-controlled copolymerization

Compared to random copolymers, Uyama and co-workers found that introducing sequence-controlled segments into commercial biodegradable plastics such as PLA can better tune the mechanical properties.^174–176^ For example, sequence-controlled PEG-PLA alternating multiblock copolymers showed higher structural regularity and stronger crystallization than random analogues, forming well-defined crystalline domains that enhanced ductility, toughness and wet-state durability compared with random analogues and neat PLA.^175^ Notably, copolymers exhibited substantially greater mass loss than neat PLA with proteinase K in Tris–HCl buffer (pH 8.5) at 37 °C, owing to the increased hydrophilicity and reduced crystallinity introduced by PEG. Among them, the alternating copolymers degraded slightly slower than random counterparts due to their higher crystallinity and lower hydrophilicity. On the other hand, they prepared 3HB-PLA alternating multiblock copolymers by initiating l-lactide polymerization with 1,4-butanediol bis(3-hydroxybutanoate) as linear 3HB diol or 1,4-cyclohexanedimethanol bis(3-hydroxybutanoate) as cyclic 3HB diol, followed by HDI-mediated chain extension.^176^ Introducing 3HB lowered *T*_g_, disrupted crystallization and afforded more transparent films. At the same PLA segment length, copolymers with linear 3HB showed higher stretchability than cyclic analogues, likely due to its greater chain mobility and conformational flexibility, with the highest toughness of 49.5 MJ m^−3^, far exceeding that of neat PLA (1.54 MJ m^−3^). Importantly, 3HB incorporation reduced crystallinity and enhanced surface wettability, while the lower steric hindrance of linear 3HB further facilitated proteinase K attack in Tris–HCl buffer (pH 8.5) at 20 °C. As a result, the copolymer containing linear 3HB reached complete mass loss within 10 days, whereas its cyclic counterpart required 20 days, while neat PLA showed less than 10% mass loss after 30 days.

To retain the biodegradability and gas barrier performance of PGA while alleviating its brittleness, Zhang and co-workers introduced a soft polybutylene carbonate (PBC) middle block to obtain PGA-PBC-PGA triblock copolymers.^177^ As the PGA block length increased, the PGA-related *T*_g_ and thermal stability gradually rose and approached those of PGA, while the continuous phase evolved from a flexible PBC-dominated morphology to a more brittle PGA-dominated one. At suitable PGA contents, the copolymers combined tensile strengths of 27.5–37.4 MPa with fracture strain of 149–409%, while more PGA-rich compositions showed excellent O_2_ and CO_2_ barrier properties, outperforming PBS, PBAT, and PLA. The copolymers exhibited 13.4–63.4% mass loss with *Pseudomonas* lipase in phosphate buffer (pH 7.4) at 37 °C after 28 days, with greater loss at higher PGA contents attributed to preferential PGA hydrolysis.

In addition, sequence-controlled copolymers can enable regioselective enzymatic cleavage to generate sequence-defined degradation products. Foster and co-workers showed that sequence-controlled copolyamides underwent regioselective hydrolysis by nylon hydrolases in phosphate buffer (pH 7.4) at 65 °C.^178^ Although the yield of soluble products was below 5 mol%, sequence-defined tetrads accounted for over 90% of the detected products, whereas the statistical copolymer yielded substantially fewer tetrads. Different hydrolases favored distinct tetrad products, demonstrating enzyme-dependent control over cleavage pathways and product selectivity. Although the low yield precluded direct repolymerization of the enzymatic hydrolysates, a chemically synthesized representative tetrad was repolymerized into a copolyamide with largely preserved sequence and material properties, highlighting the potential of sequence-controlled polymers for programmable enzymatic deconstruction and sequence-preserving recycling.

## 3. Physical blending

Physical blending refers to incorporating additional components without altering the chemical structure of the matrix, and is generally simpler and more versatile than chemical modification.^24,68,69^ By introducing fillers such as polymers or live components, the morphology, properties, and functions of plastics can be finely tuned.^24,69,179,180^ In this section, we summarize and discuss how physical blending enables the simultaneous regulation of performance, functionality, and enzymatic degradability (Table 4 and 5).

**Table 4:** Effects of physical blending on the properties and enzymatic degradability of natural polymers as matrix phases

Matrix phase	Additive phase	Effects on physical properties	Effects on enzymatic degradation	Ref.
Starch	Cellulose nanocrystals (CNCs)	CNCs at 1.5–2.5 wt% preserved starch crystallinity, short-range organization, and surface homogeneity while reducing swelling, whereas CNCs at 5–10 wt% promoted CNC aggregation, disrupted starch ordering, and increased surface defects and swelling	All blends were hydrolyzed at 85–90% (±5%) with porcine pancreatic α-amylase in phosphate buffer (pH 7.0) at 37 °C after 48 h. CNCs at 1.5–2.5 wt% slowed early-stage enzymatic hydrolysis, whereas CNCs at 5–10 wt% accelerated early-stage hydrolysis	183
	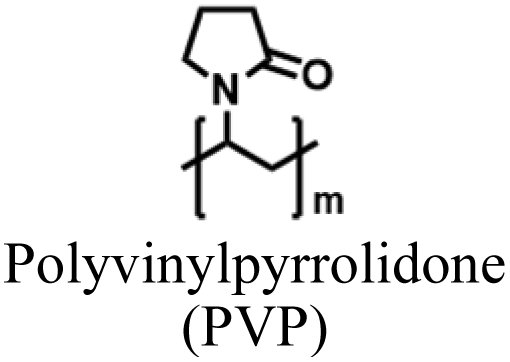	PVP incorporation enhanced *T*_g_, thermal stability, and tensile strength, but reduced water absorption and stretchability	PVP incorporation slightly reduced the mass-loss rate, but the blends still underwent complete mass loss with crude amylase within 24 h or with crude cellulase within 36 h from *Penicillium oxalicum* in phosphate buffer at 50 °C	184
	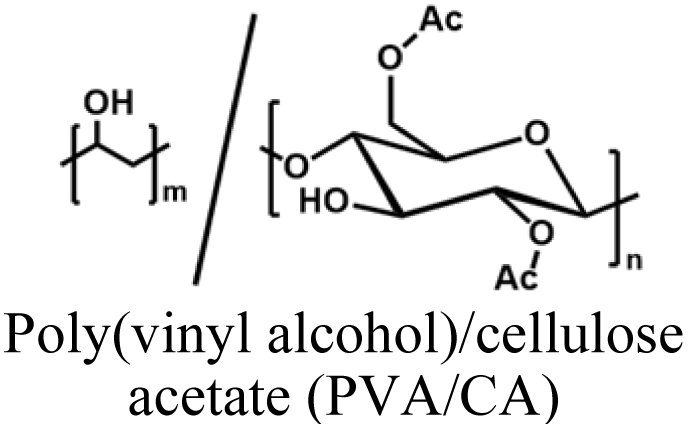	Increasing starch content enhanced water uptake and hydrophilicity but reduced fracture strain, while tensile strength and Young's modulus were optimized at the 1 : 1 blend	The 1 : 1 blend released the highest glucose concentration (131.5 mg dL^−1^), whereas the 1 : 2 blend showed the highest mass loss (50.24%) with amylase/cellulase in acetate buffer (pH 5.5) at 30 °C after 72 h	185
Cellulose	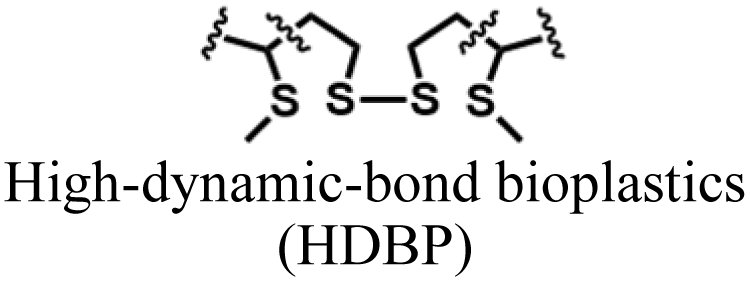	Dual-network formation enhanced thermal stability (*T*_g_ and initial decomposition temperature, HDBP > LDBP), crystallinity (LDBP > HDBP), and tensile strength (HDBP > LDBP), while LDBP exhibited higher stretchability	HDBP and LDBP showed substantial mass loss with cellulase from *Trichoderma* sp. in sodium acetate buffer (pH 5.0) at 50 °C after 72 h, with HDBP showing faster mass-loss rate	186
	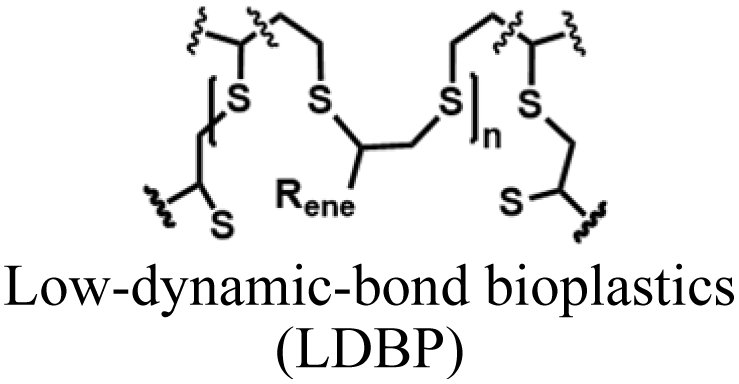			
	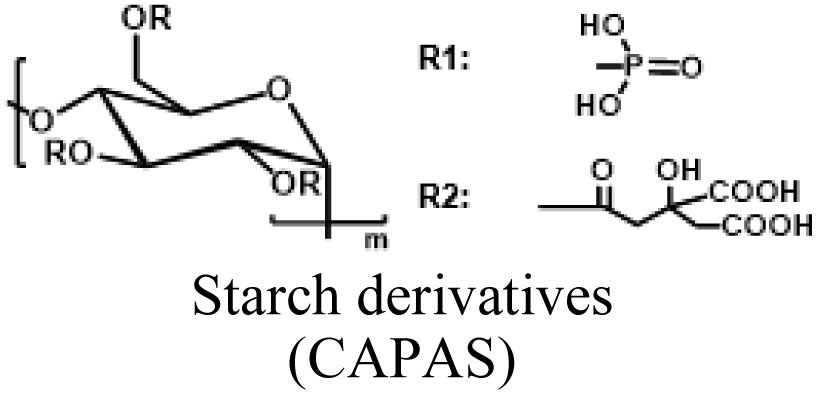	CAPAS incorporation improved surface smoothness, compactness, hydrophobicity, thermal stability, and mechanical performance, while the CAPAS-CAc coating enhanced the compressive strength of urea particles and enabled slower N release	CAPAS-CAc film showed a degradation profile similar to that of the CAc film, with 54.47% mass loss with cellulase/α-amylase in phosphate buffer (pH 7.0) after 28 days	187
Soy protein isolate (SPI)	SF	Prolonged ultrasonic treatment enabled the formation of uniform, bead-free nanofibers and enhanced β-sheet content, thermal stability, hydrophilicity, water retention, modulus, strength, and elongation	Increasing ultrasonic time improved enzymatic degradation, raising mass loss nearly threefold to 58.35% with lysozyme in phosphate buffer (pH 7.4) at 37 °C after 10 days	188
Silk fibroin (SF)	Tussah silk fibroin/Bombyx mori silk fibroin (3 : 1)	Moderate ultrasonic treatment (450 W) enabled more uniform fibers with reduced diameter and increased surface area, mitigated phase separation, and enhanced β-sheet content, hydrophilicity, thermal stability, mechanical properties, and cell viability	Increasing ultrasonic power improved enzymatic degradation, raising mass loss to 60.32% in protease solution at 37 °C after 5 days	189

### 3.1 Polymer blending

Polymer blending is an efficient strategy for integrating the complementary properties of different polymers without complex molecular redesign or polymer synthesis. By blending polymers with distinct mechanical, thermal, and degradability profiles, one can simultaneously improve toughness, stiffness, processability, and sustainability.^69,179^ Depending on the thermodynamic compatibility, blends may be fully miscible, partially miscible, or immiscible, which strongly affects their morphology, properties, and degradability (Tables 4 and 5).

#### 3.1.1 Natural polymer matrices

Starch is an abundant plant-derived carbohydrate polymer that has attracted broad interest as a biodegradable plastic because of its low cost, thermoplasticity, oxygen-barrier performance, and water solubility.^30^ However, the poor water resistance, weak mechanical properties, and limited thermal stability of native starch have motivated extensive efforts to improve its performance through polymer blending.^181,182^ For example, Lourdin and co-workers extruded glycerol-plasticized thermoplastic starch with cellulose nanocrystals (CNCs).^183^ At low loadings (1.5–2.5 wt%), the apparently well-dispersed CNCs formed hydrogen-bonding interactions with starch, reinforcing the matrix while reducing swelling and slowing hydrolysis by porcine pancreatic α-amylase in phosphate buffer (pH 7.0) at 37 °C. Higher CNC loadings (5–10 wt%) promoted nanocrystal aggregation and generated voids and structural discontinuities, thereby increasing swelling and accelerating enzymatic hydrolysis. Sharma and co-workers prepared glycerol-plasticized starch/poly(vinylpyrrolidone) (PVP) blend films by solution casting.^184^ Partial hydrogen bonding between starch and PVP increased tensile strength and thermal stability relative to pure starch but reduced stretchability. In phosphate buffer at 50 °C, starch/PVP and pure starch films reached complete mass loss within 24 and 18 h, respectively, with crude amylase from *Penicillium oxalicum*, while crude cellulase required 36 h for complete mass loss of the blend. Similarly, Abu-Saied and co-workers blended starch extracted from waste potato peel with PVA/CA by solution casting, where hydrogen-bonding interactions enhanced tensile strength and Young's modulus, with the 1 : 1 blend showing optimal mechanical performance.^185^ Under treatment with amylase and cellulase in acetate buffer (pH 5.5) at 30 °C, the films released glucose and lost mass, with the 1 : 1 blend giving the highest glucose yield and the 1 : 2 blend showing the greatest mass loss (Table 4).

Beyond chemical modification discussed in Section 2.1.1, physical blending also provides a convenient route to tune cellulose-based materials. Hong and co-workers introduced high-dynamic-bond bioplastics (HDBP) containing dynamic disulfide bonds, and low-dynamic-bond bioplastics (LDBP) with permanent C–S linkages, by incorporating a secondary covalent network into cellulose to form dual-network bioplastics with improved thermal stability.^186^ The dynamic HDBP network promoted mechanical energy dissipation, giving an ultimate tensile strength of 193 MPa, whereas the more permanent LDBP network afforded a rigid structure with a strength of 66 MPa; both exceeded cellulose bioplastic (8.8 MPa). Although these biocompatible materials remained degradable by cellulase in sodium acetate buffer (pH 5.0) at 50 °C, introduction of the second network slowed the mass-loss process. Pure cellulose exhibited nearly complete mass loss within 18 h, whereas the double-network materials required a longer degradation time, with HDBP degrading faster because of its lower crystallinity and greater cellulose accessibility. Zhang and co-workers developed a fully bio-based slow-release urea fertilizer by coating urea with CAPAS-CAc, composed of citric acid/phytic acid co-esterified starch (CAPAS) and cellulose acetate (CAc).^187^ Under cellulase/α-amylase treatment in phosphate buffer (pH 7.0), the CAPAS-CAc film showed a mass-loss profile trend similar to CAc over 28 days. Its initially rapid and subsequently slower mass loss was attributed to preferential removal of surface-accessible CAPAS, followed by restricted enzyme accessibility within the dense crosslinked network. Hydrogen-bonding interactions between CAPAS and CAc produced a denser coating with enhanced mechanical strength, hydrophobicity, and granule compressive strength. Consequently, CAPAS-CAc@urea released nitrogen approximately five times more slowly than uncoated urea in water and soil, enabling sustained nitrogen supply, improved root development, and enhanced fertilizer utilization.

**Fig. 7:**
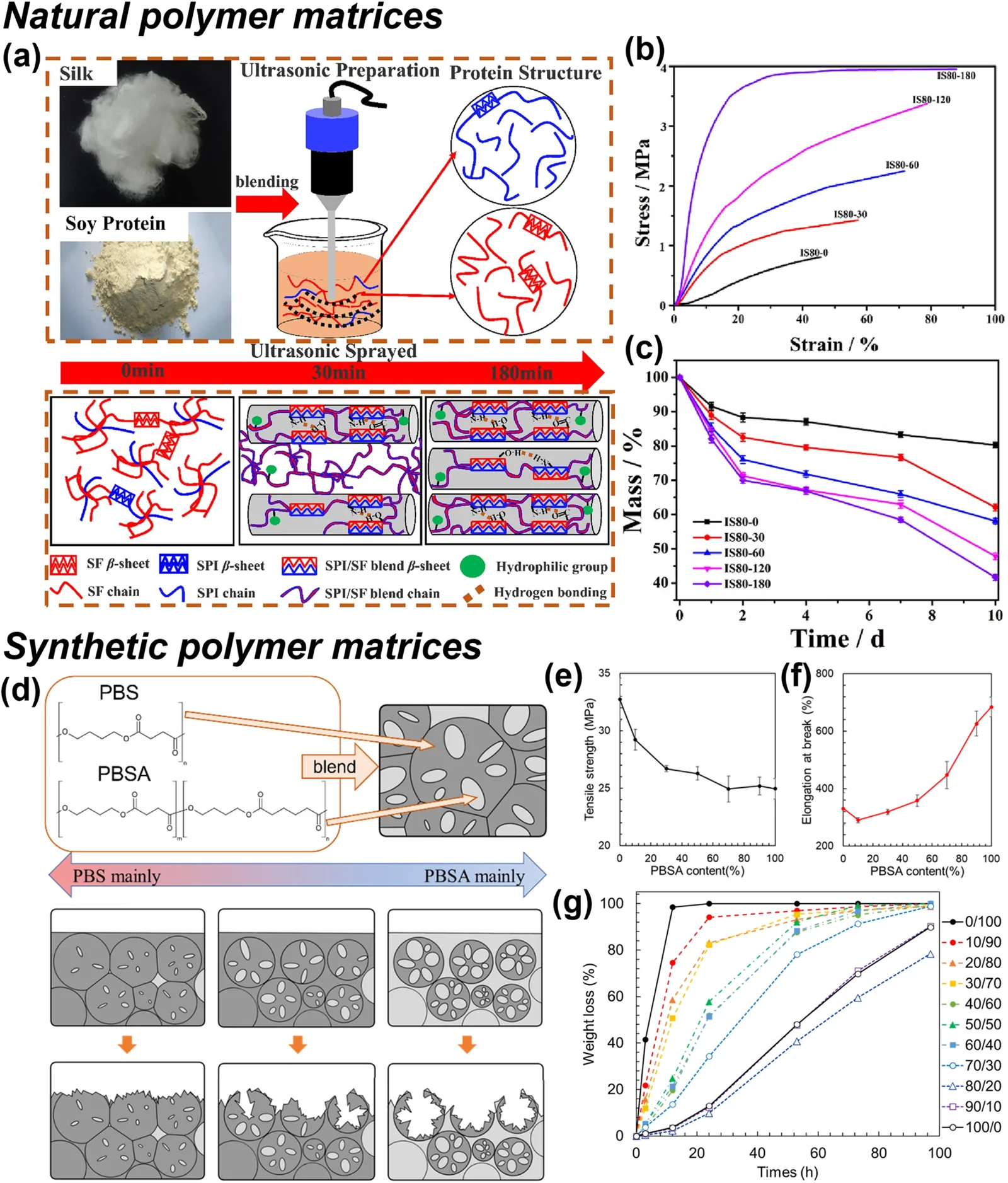
(a) Polymer blending with natural polymer matrices, and their (b) mechanical properties and (c) enzymatic degradation. (Reproduced from ref. 188 under a CC BY-NC-ND 4.0 license). (d) Polymer blending with synthetic polymer matrices, and their (e and f) mechanical properties and (g) enzymatic degradation. (Reproduced from ref. 194 under a CC BY 4.0 license).

To address the poor spinnability and mechanical weakness of soy protein isolate (SPI), Hu and co-workers blended SPI with fiber-forming SF and used ultrasound-assisted air-jet spinning to fabricate composite nanofiber membranes (Fig. 7a).^188^ Ultrasound increased solution viscosity, suppressed bead formation, and promoted a random coil/helix to β-sheet transition, while prolonged treatment enhanced intermolecular hydrogen bonding, facilitated the fabrication of uniform nanofiber membranes, and improved mechanical properties (Fig. 7b), increasing the elastic modulus from 2.04 to 21.65 MPa, ultimate strength from 0.801 to 3.981 MPa, and elongation from 46.46% to 89.16%. After 10 days in lysozyme-containing buffer (pH 7.4) at 37 °C, the 180 min sonicated membrane exhibited approximately threefold greater mass loss than the untreated sample (Fig. 7c), mainly owing to its larger surface area, higher hydrophilicity, and greater enzymatic accessibility. They further applied ultrasound-assisted solution spinning to fabricate Tussah silk fibroin/Bombyx mori silk fibroin (TSF/BSF) composite nanofibers.^189^ Moderate ultrasound treatment (450 W) significantly enhanced intermolecular hydrogen bonding and electrostatic interactions, promoted more uniform fiber diameters and denser interwoven structures, and reduced microphase separation, thereby optimizing mechanical performance. Increasing ultrasound power also increased mass loss in protease solution at 37 °C, mainly because ultrasound increased surface area, exposed more polar groups, improved hydrophilicity, and enhanced enzymatic accessibility.

#### 3.1.2 Synthetic polymer matrices

Although PLA is widely used as a biodegradable plastic, its practical applications remain limited by brittleness, motivating polymer blending as an effective strategy to improve the toughness and ductility.^33,34,190^ Williams and co-workers synthesized poly(ester-*alt*-ether)-*b*-PLLA block copolymers from commercial diglycolic anhydride (or maleic anhydride), 1,2-epoxy-9-decene and l-lactide, and used them as toughening agents for commercial PLLA (Table 5).^191^ At 15 wt%, the toughening effect was optimal, while the blend retained high stiffness, crystallinity, and thermal properties comparable to those of PLLA, with 7- and 8-fold increases in elongation and toughness, respectively. Uniformly dispersed block copolymer particles cavitated and debonded during tensile deformation, promoting shear yielding and energy dissipation. The blends were efficiently chemically recycled to l-lactide and recoverable block copolymer. With HiC in phosphate buffer (pH 7.2) at 37 °C, the block copolymer additive remained enzymatically degradable, although the PLLA-rich blend degraded more slowly because of the high-molecular-weight and semicrystalline PLLA matrix. A similar crystallinity effect was observed by Polak-Kraśna and co-workers, who showed that crystallization slowed enzymatic degradation in PLLAcoCL/PDLA blends.^192^ Langmuir film experiments with Proteinase K in phosphate buffer (pH 7.4) at 21 °C showed that PDLA, PLLA, and the 95 : 5 PLLAcoCL/PDLA blend crystallized almost immediately after enzyme addition, whereas PLLAcoCL and other blends crystallized more gradually, likely limiting further enzymatic hydrolysis. Overall, these studies highlight crystallinity as a key parameter governing PLA enzymatic biodegradability.

**Table 5:** Effects of physical blending on the properties and enzymatic degradability of synthetic polymers as matrix phases

Matrix phase	Additive phase	Effects on physical properties	Effects on enzymatic degradation	Ref.
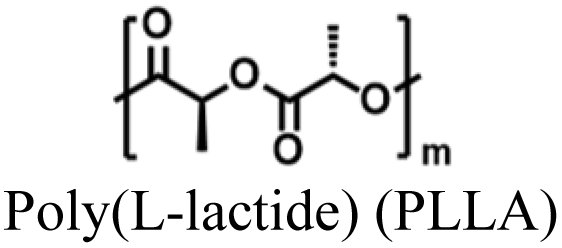	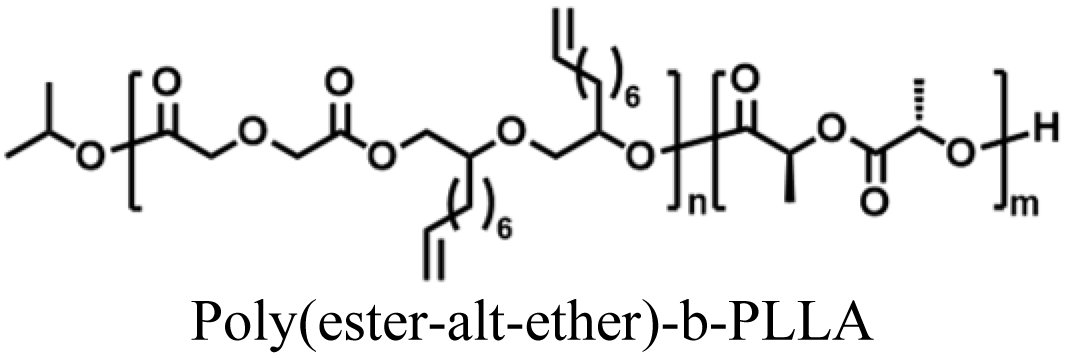	Block copolymer addition at 15 wt% increased PLLA crystallinity and markedly improved ductility and toughness, while maintaining high tensile strength and Young's modulus	PLLA degraded slowly, while the poly(ester-*alt*-ether) block in the blend showed ∼23% degradation with HiC in phosphate buffer (pH 7.2) at 37 °C after 34 days	191
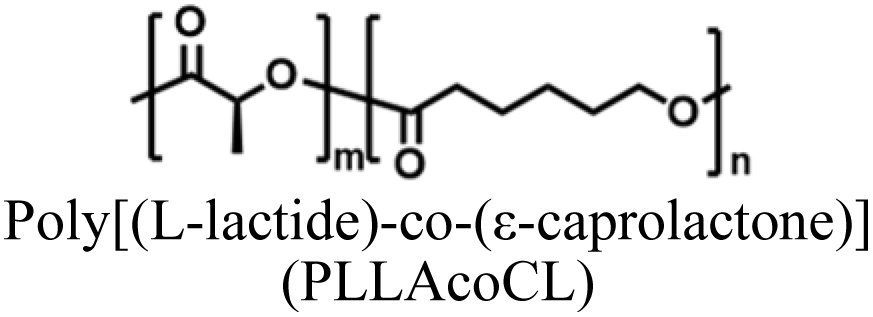	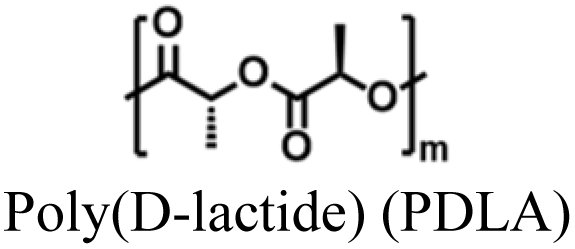	The 95 : 5 blend (PLLAcoCL : PDLA) exhibited higher initial crystallinity and superior extensibility, with fracture strain of 724% for film and 548% for meshes	All blends showed limited mass loss in Langmuir film experiments with Proteinase K in phosphate buffer (pH 7.4) at 21 °C	192
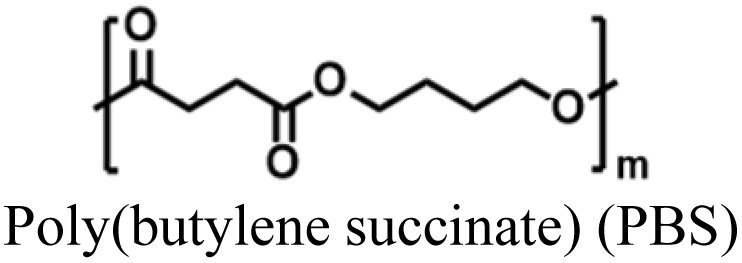	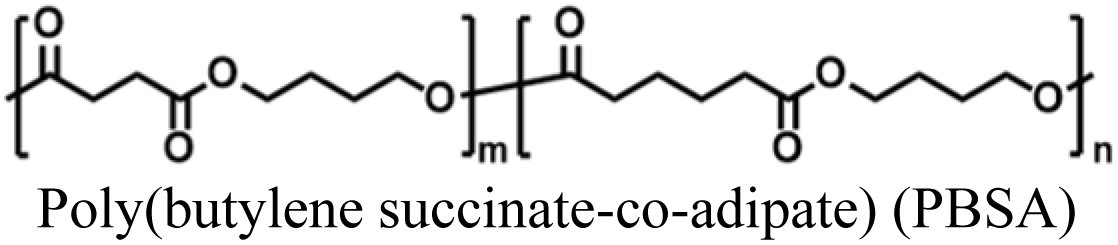	Blends were miscible in the amorphous state but phase-separated during crystallization, forming a proposed “third phase”. Increasing PBSA content reduced *T*_g_, Δ*H*_m_, strength, and stiffness, but increased ductility	Low PBSA content suppressed mass loss relative to the two-phase independent model, whereas PBSA contents above 30% increased later-stage mass loss with lipozyme CALB solution (pH 7.5) at 37 °C	194
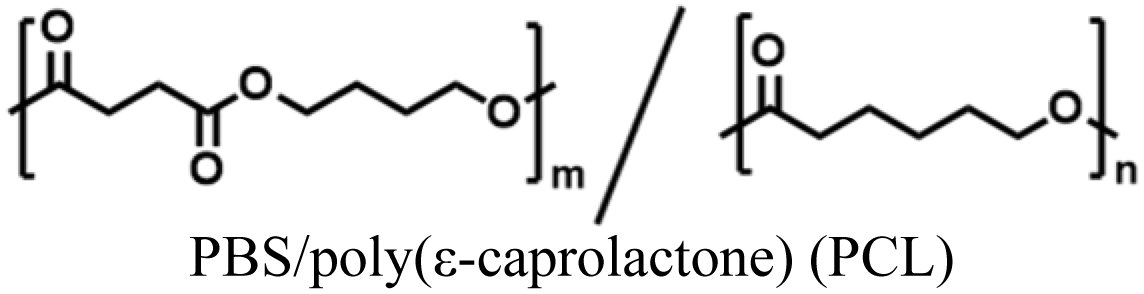	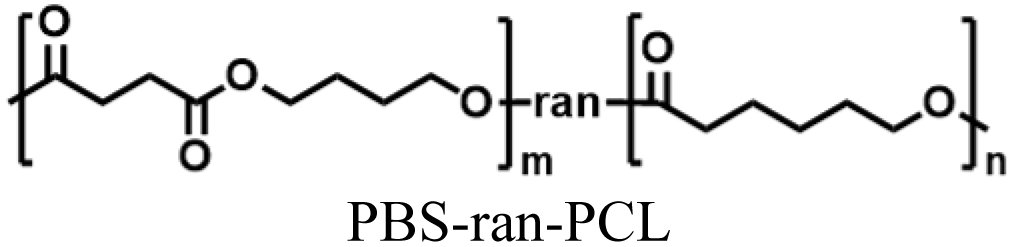	Copolymers promoted partial miscibility and induced quadrant-specific spherulites, while BS-rich copolymers enhanced PBS crystallization, favored β-form PBS crystals, increased the lamellar long period, and restricted PCL crystallization	Copolymers incorporation improved enzymatic degradation, with the CL-rich copolymer approaching the degradation of neat PCL (∼61% mass loss) with *Pseudomonas cepacia* lipase in phosphate buffer (pH 7.0) at 37 °C after 35 days	195
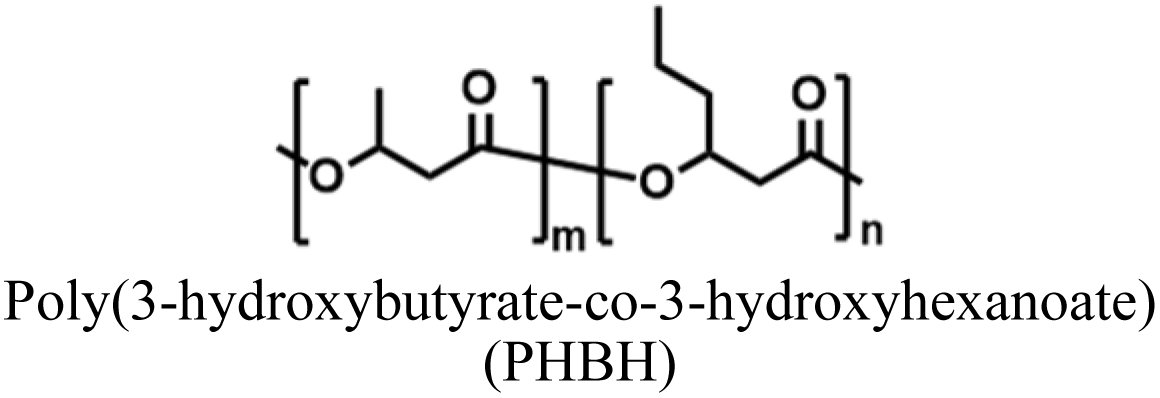	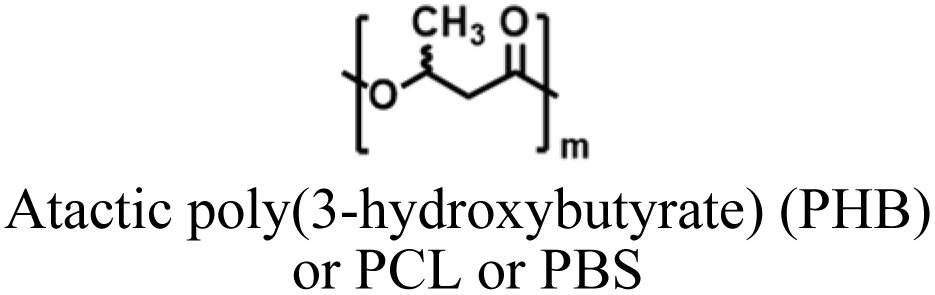	Atactic PHB was miscible with PHBH, increasing *T*_g_ and reducing PHBH crystallinity. PCL was immiscible with PHBH, forming trapped domains in PHBH-5% spherulites or dispersed granular domains in PHBH-17%. PBS was partially miscible with PHBH-5% and suppressed its cold crystallization, but was immiscible with PHBH-17%, where spherulitic morphologies varied between banded and non-banded textures	With PHB depolymerase in phosphate buffer (pH 7.4) at 37 °C, atactic PHB accelerated degradation, with the highest mass-loss rate for the 60/40 PHBH-5%/atactic PHB blend. PCL suppressed the degradation of PHBH-5% but accelerated that of PHBH-17%. With lipase in phosphate buffer (pH 7.0) at 50 °C, the degradation of PHBH/PCL blends increased with increasing PCL content, whereas PHBH-5%/PBS blends showed negligible mass loss	196
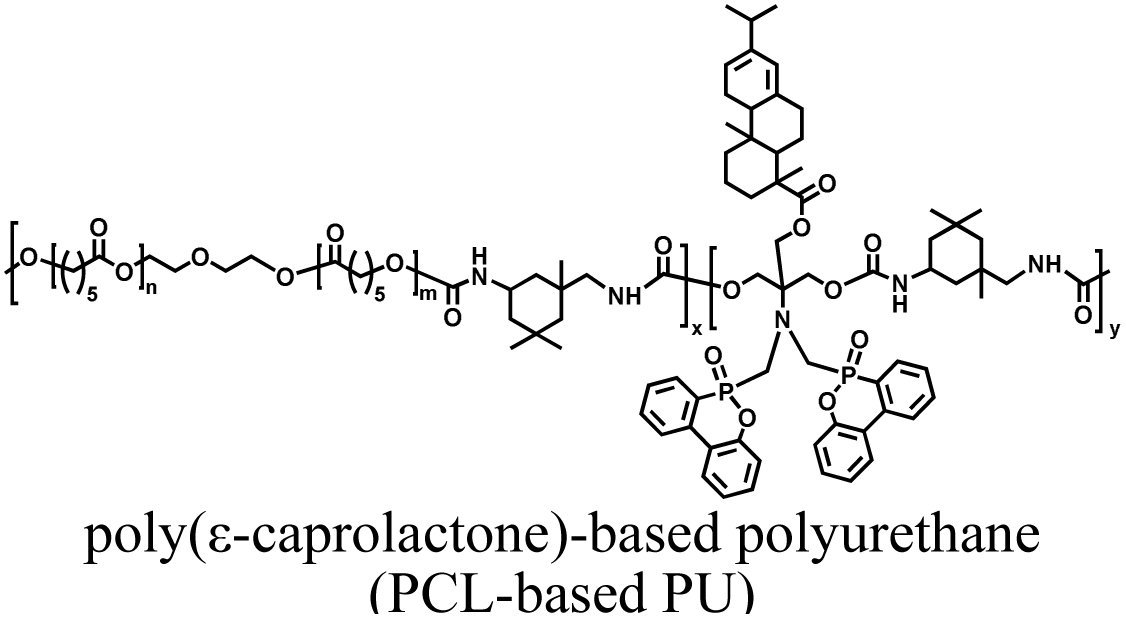	Cellulose nanocrystals (CNC)	CNC incorporation enhanced hydrogen bonding, transparency, *T*_g_, creep resistance, storage modulus, stiffness, and tensile strength, and reduced elongation, hydrophobicity, hard domain size and melt dripping	Increasing CNC content improved enzymatic degradation, raising mass loss from ∼60% to ∼100% in lipase solution at 37 °C after 60 days	197
PCL	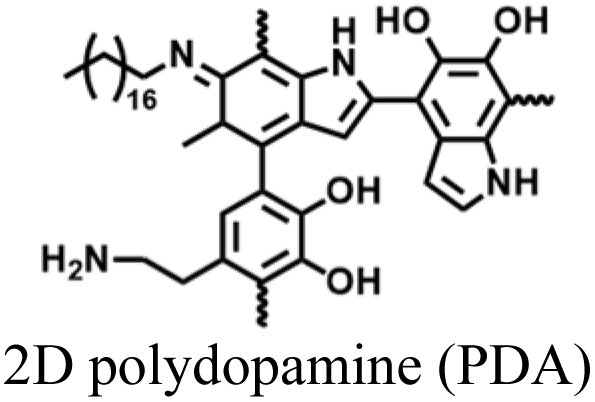	PDA incorporation, combined with in-plane stretch-confined assembly, enhanced the orientation degree, tensile strength, Young's modulus, toughness, and gas barrier properties	PDA incorporation enabled self-catalytic enzymatic degradation of the composite, which exhibited complete mass loss with lipase in phosphate buffer (pH 7.2) at 37 °C within 5 weeks	198

Blending is also widely used to tune the mechanical properties, crystallization behavior, and biodegradability of PBS.^38,193^ Iwata and co-workers incorporated poly(butylene succinate-*co*-adipate) (PBSA) into PBS (Fig. 7d), which reduced crystallinity and shifted the mechanical properties toward those of PBSA, increasing stretchability while lowering tensile strength and modulus (Fig. 7e and f).^194^ Although PBSA lost mass much faster than PBS in Lipozyme CALB solution (pH 7.5) at 37 °C, the blend behavior was not simply additive (Fig. 7g). Low PBSA contents suppressed mass loss, whereas 30–60 wt% PBSA initially showed lower but subsequently higher mass loss than predicted by the independent two-phase model. This two-stage behavior was attributed to amorphous miscibility and crystallization-induced phase separation, forming PBS spherulites that encapsulated PBSA-rich domains and created a “third-phase” structure, in which enzymes first attacked the slowly degrading PBS-rich shell before exposing the more degradable inner PBSA phase. Müller and co-workers addressed the immiscibility of PBS/PCL blends by introducing 10 wt% isodimorphic PBS-*ran*-PCL copolyesters as compatibilizers.^195^ These copolymers promoted partial miscibility, shifted the *T*_g_ values of the two phases toward each other, and induced quadrant-specific spherulites. BS-rich copolymers cocrystallized with PBS, promoting lamellar twisting, β-form-rich banded quadrants, increased long periods, and enhanced PBS-phase crystallization while restricting PCL crystallization. The ternary blends exhibited substantially greater mass loss than the binary PBS/PCL blend with *Pseudomonas cepacia* lipase in buffer (pH 7.0) at 37 °C, with CL-rich copolymers approaching neat PCL. This enhancement was attributed to reduced PCL crystallinity, looser β-form PBS packing, and the high lipase susceptibility of CL-rich segments.

Abe and co-workers systematically examined poly(3-hydroxybutyrate-*co*-3-hydroxyhexanoate) (PHBH) containing 5 or 17 mol% 3-hydroxyhexanoate (3HH) blended with atactic PHB, PCL, or PBS, and evaluated degradation by PHB depolymerase in phosphate buffer (pH 7.4) at 37 °C and, for PCL- and selected PBS-containing blends, by lipase in phosphate buffer (pH 7.0) at 50 °C.^196^ PHBH and atactic PHB were miscible in the homogeneous amorphous phase; increasing atactic PHB reduced crystallinity and accelerated PHB depolymerase degradation, although higher enzymatically inactive S-3HB contents depressed degradation, giving a maximum at 40 wt% atactic PHB. PHBH/PCL blends were immiscible: PCL domains were retained within crystalline PHBH-5%, slowing degradation, whereas detachable PCL domains in PHBH-17%/PCL exposed fresh PHBH surfaces and accelerated mass loss. Lipase degradation increased with PCL content and generated large pores. PHBH-5%/PBS degraded slowly because intertwined crystalline phases limited PHBH exposure and PBS was poorly hydrolyzed. Quantitative analysis of the water-soluble degradation products further supported the proposed degradation mechanisms in the blended systems and revealed that mass loss alone could overestimate the actual extent of enzymatic degradation.

Blending can also improve the mechanical and degradative performance of PCL-based systems. Song and co-workers incorporated CNCs into a PCL-based PU to form hydrogen-bonding crosslinking networks, which increased stiffness and creep resistance while retaining good stretchability.^197^ The PU containing 5 wt% CNC underwent nearly complete mass loss in lipase solution at 37 °C within 60 days, whereas the CNC-free PU showed only ∼60 wt% mass loss. This enhancement was attributed primarily to the wicking effect of CNCs, which facilitated water uptake and enzyme penetration into the PCL-based PU matrix. The material also showed flame retardancy, anti-dripping behavior, recyclability, and use as a fire-safe encapsulant for pressure sensors. Wang and co-workers incorporated 2D polydopamine (PDA) nanosheets into PCL *via* evaporation-induced assembly followed by in-plane stretch-confined alignment, creating a dense, low-porosity composite with PDA-PCL hydrogen bonding.^198^ The aligned nanosheets improved gas barrier properties and enabled efficient stress transfer, increasing strength and toughness while retaining high stretchability. Although the densely aligned structure initially impeded water and lipase diffusion in phosphate buffer (pH 7.2) at 37 °C, the films subsequently exhibited accelerated mass loss and nearly disappeared within five weeks. This proposed self-catalytic behavior was attributed to a locally acidic environment generated by carboxyl-containing PCL hydrolysis products, which induced pH-responsive dissociation of PDA and facilitated further hydrolysis. However, neither the local pH change nor PDA-derived degradation products were directly characterized.

Polymer blending has become a well-established and widely adopted platform for tailoring polymer morphology, mechanical performance, and enzymatic degradability. However, most studies have primarily focused on overall mass loss or degradation rate, while the fate of individual blend components and the recovery of degradation products remain poorly understood. Components resistant to the selected enzyme, or whose degradation has not been demonstrated, may persist or detach as insoluble fragments. These residual species may complicate the separation, purification, and reuse of soluble products, yet their chemical integrity and recoverability are rarely evaluated. Future studies should therefore establish component-specific mass balances, identify both soluble and insoluble products, and evaluate whether residual additives can be recovered and reused, to assess the true circularity and sustainability of blended polymer systems.

### 3.2 Enzyme-embedded plastics

Embedding the corresponding enzymes into biodegradable plastics represents a promising strategy for enabling rapid bulk degradation under realistic environmental conditions, thereby alleviating the environmental burden caused by their intrinsically slow degradation and consequent microplastic accumulation.^64,70,180^ The main challenges lie in preserving enzyme activity during processing, achieving uniform enzyme dispersion in the polymer matrices, and maintaining material performance during service.

Iwata and co-workers have made significant contributions in this field by employing multiple effective mixing methods to embed corresponding enzymes into various plastics and investigate their effects on material properties and self-degradation behaviors.^180^ In 2020, they embedded small amounts of Proteinase K into PLLA films by solvent casting or by melt extrusion at 200 °C, and the enzyme retained activity within PLLA after processing to achieve PLLA self-degradation in buffer.^199^ Several lipases (Lipase AK Amano, Lipase G Amano 50, Lipase PS Amano SD and Lipozyme CALB L) were further individually incorporated into different aliphatic biodegradable polyesters (PBS, PBSA, and PCL), and the resulting enzyme-embedded films preserved acceptable mechanical properties while exhibiting self-degradation in buffer because the lipases retained activity after thermal extrusion.^200^ Moreover, they developed enzyme-embedded films of PDLA, PBS, PBSA, PCL, PBAT and PEF using low loadings of HiC, which demonstrated efficient self-degradation in seawater.^201^ Most recently, to improve thermal stability and dispersion, they chemically conjugated or physically coated the HiC with polyethylene glycol chains before being embedded into the films.^202,203^ Compared with blending pure HiC, these strategies improved the mechanical properties of PDLA, PBS, and poly(ethylene succinate) (PES) and optimized their enzymatic efficiency for self-biodegradation.

**Fig. 8:**
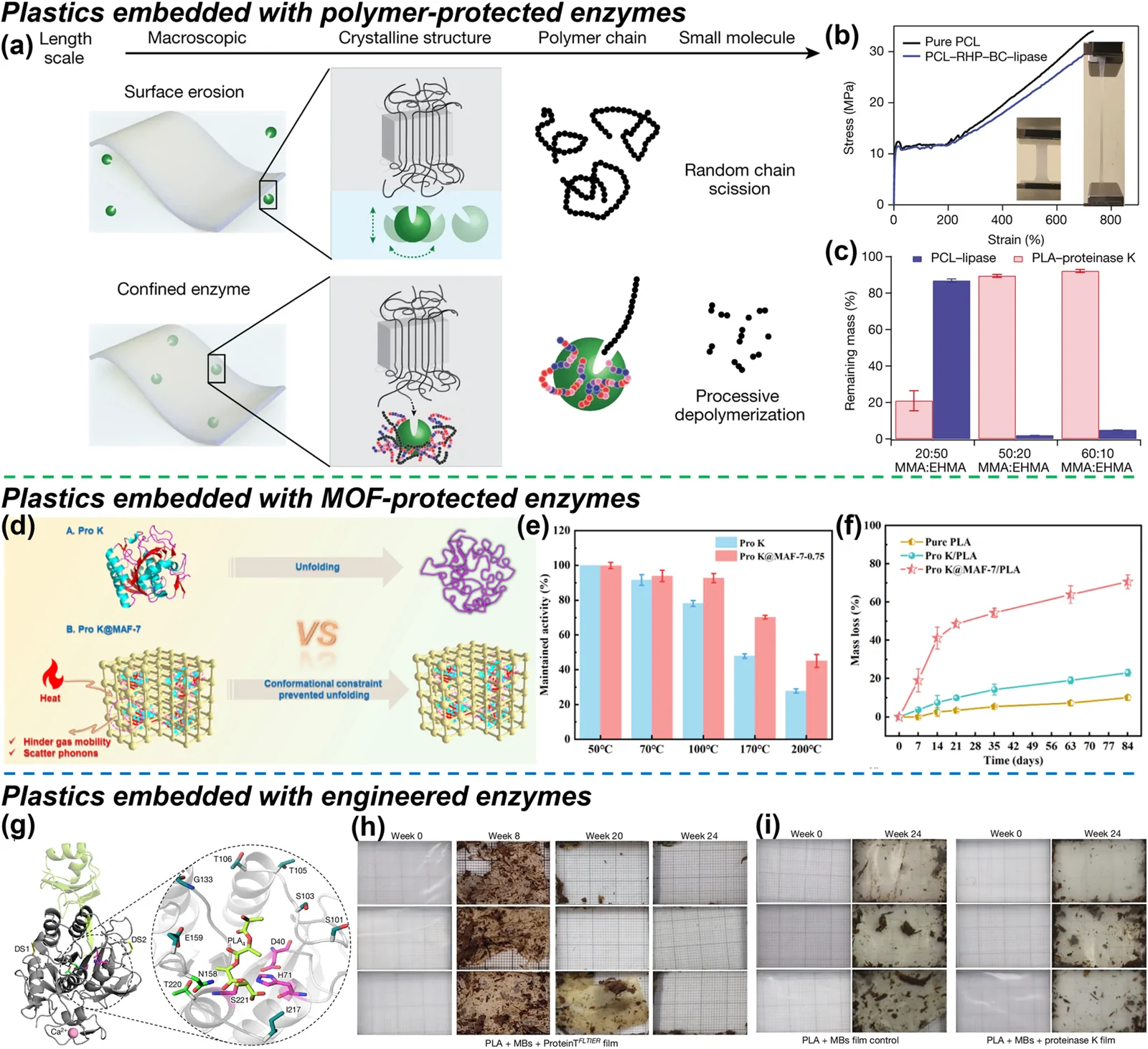
(a) Schematic illustrating degradation pathways of plastics with or without embedded polymer-protected enzymes. (b) Mechanical properties and (c) self-degradation efficiency of plastics embedded with polymer-protected enzymes. (Reproduced with permission from ref. 70. Copyright 2021, Springer Nature). (d) Schematic illustration of the dual thermal protection effect, and (e) the maintained activity (after thermal treatment) of the MOF-protected enzyme (pro K@MAF-7). (f) Comparison of self-degradation efficiency between plastics embedded with MOF-protected enzymes and control groups. (Reproduced with permission from ref. 207. Copyright 2025, American Chemical Society). (g) Structural insight into the engineered enzyme (PAM PLAase). Comparison of self-degradation efficiency between (h) plastics embedded with engineered enzymes and (i) control groups. (Reproduced with permission from ref. 64. Copyright 2024, Springer Nature).

Xu and co-workers pioneered the embedding of random heteropolymer (RHP)-protected enzymes into polyesters through solution casting or melt extrusion (Fig. 8a).^70^ The nanoscale enzyme dispersion preserved PCL crystallization and mechanical properties, thereby maintaining material usability while enabling programmed degradation (Fig. 8b). Enzyme active-site architecture and RHP composition were both critical for the enzymatic degradation control.^204^ BC-lipase, featuring a deep and narrow active-site cleft, promoted chain-end-mediated processive depolymerization, enabling PCL containing only ∼0.02 wt% enzyme to reach up to 98% conversion within 24 h and yielding mainly oligomers below 500 Da, which were recovered and repolymerized as a proof of concept. RHPs served not only as protectants and dispersants that retained enzyme activity after processing, but also as modulators of enzyme-polymer interactions, with suitable RHP compositions enabling the formation of proteinase K-RHP-PLA complexes that promoted processive PLA depolymerization (Fig. 8c). Degradation could be programmed by crystallization temperature, operation temperature, and RHP hydrophobicity, providing catalytic latency during storage/use and triggered depolymerization under selected conditions. However, this strategy was ineffective for inert polyolefins such as PE, where weak enzyme-polymer association prevented the generated radicals from modifying hydrocarbon chains.

Beyond synthetic plastics, enzyme embedding has also been extended to biopolymers. Hakkarainen and co-workers physically entrapped immobilized *Candida rugosa* lipase in CA particles and incorporated the immobilized lipase (IL) into CA films, thereby accelerating deacetylation and subsequent cellulase-mediated degradation.^205^ After 40 days in phosphate buffer (pH 7.4) containing lipase and cellulase at 37 °C, CA containing 5 wt% IL (CA/IL 5%) showed an 88% reduction in *M*_n_, sharp DS decrease, and complete surface fragmentation. Under simulated composting at 58 °C, CA/IL 5% films underwent nearly complete macroscopic disintegration after 180 days, whereas neat CA remained largely intact. However, IL incorporation reduced thermal stability, and mechanical properties were not evaluated. Kaplan and co-workers incorporated 1 wt% protease XIV or α-chymotrypsin into RSF *via* thermoplastic molding to fabricate enzyme-embedded silk reservoirs.^206^ The amphiphilic silk matrix protected the enzymes during thermal processing, allowing residual activity, while largely retaining dry-state mechanical robustness. The resulting reservoirs showed accelerated degradation in DPBS at 37 °C, and the mass loss could be tuned by processing temperature through its effect on silk crystallinity. This system provided a modular platform for long-acting drug delivery with controlled release rate, in which SF reservoir preserved the bioactivity and cytotoxicity of drugs (DOX, TMZ, L-Asp) after thermal processing.

Recently, Yang and co-workers innovatively encapsulated proteinase K (pro K) in hydrophilic metal azolate framework (MAF-7) and incorporated 1 wt% pro K@MAF-7 into PLA by melt extrusion and hot pressing, corresponding to only ∼0.17 wt% pro K in the film.^207^ The mesoporous framework provided thermal protection: nanoconfinement restricted enzyme unfolding, while thermal insulation kept the upper-surface temperature of pro K@MAF-7 at only ∼120 °C after heating at 170 °C for 10 min (Fig. 8d). Consequently, pro K@MAF-7 retained ∼70% activity after processing, compared with ∼48% for free pro K (Fig. 8e). In Tris–HCl buffer (pH 8.0) at 50 °C, pro K@MAF-7/PLA films showed 70% mass loss after 84 days, approximately three times that of pro K/PLA and seven times that of neat PLA (Fig. 8f), together with an 85% decrease in *M*_w_, while cyclic lactide pentamers and linear lactide decamers were the main soluble products after 120 days. Notably, pro K@MAF-7 was uniformly dispersed without deteriorating mechanical properties, and the strategy was also applicable to lipase PS. Chen and co-workers similarly encapsulated lipase FM-10 in acid-responsive MOF (MIL-88A) and incorporated 4 wt% FM-10@MIL-88A into PCL using industry-compatible processing techniques.^208^ The MOF carrier protected and isolated the enzyme during daily use, preserving water/storage stability and causing negligible changes in the thermal and mechanical properties of PCL. Under weakly acidic rainwater conditions (pH 5.6–6.8), MIL-88A decomposed and released FM-10 and Fe^3+^, triggering rapid PCL hydrolysis. In phosphate buffer (pH 6.8) at 40 °C, FM-10@MIL-88A&PCL underwent over 98% hydrolysis within 24 h, with 6-hydroxyhexanoic acid and its oligomers (<1000 Da) identified as the hydrolysis products, whereas neat PCL showed minimal degradation. In outdoor soil, the composite degraded nearly completely within 10 days, and the resulting degradation mixture promoted maize growth. This low-cost strategy was further extended to PLA, PET, PBAT, PBS, and PEF using matched enzymes.

Beyond immobilization strategies, protein engineering provides another route to improve enzyme-embedded plastics by identifying thermostable variants that can survive high-temperature processing. Marty and co-workers screened and rationally engineered a PLA-degrading ProteinT variant with high activity at near-neutral pH and high thermostability with *T*_m_ of 79.4 °C (Fig. 8g), and incorporated it into PLA through a masterbatch-based melt extrusion at 160 °C, giving films containing only 0.02 wt% enzyme.^64^ The resulting composite films retained mechanical properties and long-term storage stability, but completely disintegrated under home-composting conditions at 28 °C within 20–24 weeks (Fig. 8h), whereas enzyme-free films and films containing commercial proteinase K showed negligible disintegration after 24 weeks (Fig. 8i). In Tris–HCl buffer (pH 9.0) at 45 °C, composite films achieved 70% conversion after 3 days and 85% after 27 days, with lactic acid and its dimer released as the soluble degradation products. Furthermore, Reisky and co-workers screened thermostable cutinases and embedded the best-performing HiC into thermoplastic PU (TPU) *via* melt extrusion.^209^ In KPi buffer (pH 8.0) at 37 °C, TPU 5 containing 0.3 wt% HiC incorporated at 150 °C showed a 32-fold increase in adipic acid release over enzyme-free TPU and nearly twofold faster adipic acid-release kinetics than externally supplied HiC, while enzyme-embedded TPU 5 largely retained its mechanical properties. Whereas external HiC mainly caused surface erosion, embedded HiC induced bulk degradation with a 54% decrease in crystalline polyester soft segments, indicating that enzymatic degradation also reached crystalline domains.

These emerging enzyme-embedding strategies with low enzyme loadings have substantially improved plastic degradability while largely preserving mechanical performance during service. Through enzyme protection and engineering, enzyme activity can be retained during plastics processing, enabling on-demand degradation under appropriate triggers. Although several studies have identified major soluble degradation products, systematic product quantification, preparative recovery, and reutilization remain limited, with only isolated proof-of-concept repolymerization demonstrated. Moreover, the fate of embedded enzymes, including their release, residual activity, and potential recovery, has rarely been examined. Future studies should therefore establish comprehensive product balances, clarify which degradation products can be purified and reused, and determine the fate of embedded enzymes to improve the circularity and sustainability of enzyme-embedded plastics.

### 3.3 Engineering living materials

Beyond enzymes, incorporating active components such as engineered cells and bacteria into polymer matrices can endow plastics with living functions, giving rise to engineered living materials (ELMs) as a new class of smart and sustainable living plastics.^210–212^ As noted in Section 2.1.2, Kaplan and co-workers developed a mild processing method of RSF that enabled stable encapsulation of living *probiotics* and *soil rhizobacteria*.^95^ The encapsulated probiotics showed markedly higher survival after simulated gastrointestinal treatment than free bacteria, inhibited *Shigella flexneri*, and improved gastrointestinal retention after oral administration in mice. Notably, encapsulated *rhizobacteria* maintained protease-secreting activity and promoted silk degradation in soil over 90 days, leaving only 9.2% residual mass compared with 82.3% for bacteria-free silk plastic, thereby highlighting the potential of living bioplastics as self-degrading materials. Nelson and co-workers utilized poly(ethylene glycol) diacrylate-modified bovine serum albumin (BSA-PEGDA) as a structural matrix to fabricate ELMs with bio-augmented mechanical properties and enhanced resistance to degradation.^213^ Building on this, they encapsulated genetically engineered *Saccharomyces cerevisiae* carrying two inducible genetic circuits within BSA-PEGDA to afford ELMs that could be fabricated into arbitrary shapes by 3D printing.^214^ One circuit, activated by Cu^2+^, induced betaxanthin production, which interacted with the protein matrix for bio-augmentation.^213^ After drying, the resulting bioplastics exhibited significantly enhanced stiffness, with a compressive modulus of 343 MPa and a Young's modulus of 262 MPa. After 60 days of use, the materials could be rehydrated and the second circuit was induced by galactose to express proteinase A,^215^ enabling on-demand degradation with 72% mass loss within 19 days.

Bacterial spores offer another attractive route to living plastics because they can withstand harsh processing conditions yet rapidly reawaken under favorable conditions to secrete degrading enzymes. Dai and co-workers blended engineered *Bacillus subtilis* spores with PCL pellets by solvent casting or melt-based 3D printing at 120 °C, producing materials that remained stable under dormant conditions, including 60 days in soda.^216^ The spores survived heat and solvent exposure, whereas free BC-lipase lost activity after thermal treatment. After surface erosion by CA-lipase, grinding, or treatment with boiling water, the released spores germinated upon xylose induction and secreted BC-lipase, which depolymerized PCL into small products below 500 Da,^70^ causing near-complete degradation of films within 6 days and 3D-printed objects within 6–7 days. In industrial compost, the living PCL films disintegrated within 30 days, faster than regular PCL. The platform was further extended to PBAT, PBS, PLA, PHA, and PET, demonstrating broad processing adaptability. Pokorski and co-workers incorporated heat-shock tolerized *Bacillus subtilis* spores, obtained by screening and adaptive laboratory evolution, into commercial TPU with 0.8 wt% spores *via* melt extrusion at 135 °C.^217^ The evolved spores remained structurally intact and nearly fully viable, germinated in autoclaved compost, and facilitated TPU disintegration, giving 92.7% mass loss after 5 months at 37 °C, compared with 63.6% for wild-type spore composites and 43.9% for neat TPU. Notably, spore incorporation improved toughness by 37%, tensile stress by 30%, and fracture strain by 12%, likely through strong spore-TPU interfacial adhesion, thereby simultaneously enhancing mechanical performance and biodegradability.

Overall, engineering living materials represents a promising strategy to create plastics with programmable biological functions by incorporating small amounts of living components into polymer matrices. A common feature of these systems is the temporal separation between material service and biological activity, enabling plastics to remain mechanically stable during use while activating specific microbial functions, such as enzyme secretion and on-demand degradation, only at the desired end-of-life stage. However, challenges associated with long-term microbial viability, biosafety, genetic stability, scalable manufacturing, and the limited characterization and utilization of degradation products remain to be addressed before widespread practical application.

## 4. Supramolecular interactions

Supramolecular interactions, including hydrogen bonding, host-guest interaction, and metal–ligand coordination, can function as sacrificial bonds to impart outstanding mechanical performance to polymers, while their dynamic nature also enables molecular recognition, self-healing, and stimuli responsiveness, thereby broadening the scope of sustainable material design.^218–226^ Importantly, such interactions can be exploited to modulate the degradability of polymeric materials, further advancing the sustainability and circularity of plastics.^60,66,77,91,220,224,226^ In this section, we summarize and discuss how the introduction of supramolecular interactions can tune the performance, functionality, and enzymatic degradation of plastics.

### 4.1 Hydrogen-bonding networks

Hydrogen bonding is a ubiquitous supramolecular interaction and plays essential roles in nature and living systems.^227,228^ As discussed in Section 2.1, many natural materials, including polysaccharides, proteins, and DNA, rely strongly on hydrogen bonding to govern their properties and degradation behaviors. Inspired by nature, introducing hydrogen-bonding networks into plastics enables the fabrication of tough and functional materials. PU is a classic example: strong hydrogen-bonding interactions often generate hard domains that enhance stiffness and durability, yet they can also hinder enzymatic access to the amorphous soft segments that serve as primary biodegradation sites.^77,229–233^ As noted in Section 2.2.3, bio-derived long-chain polyols can be used as soft segments to prepare bio-based biodegradable PUs, but examples remain relatively limited.^147^ In contrast, PCL is the most widely used soft segment in biodegradable PUs because of its tunable and flexible chain length, excellent biocompatibility, and controllable degradability.^234^ Suitable diisocyanates and chain extenders can then be selected to build the desired hydrogen-bonding architecture.

**Fig. 9:**
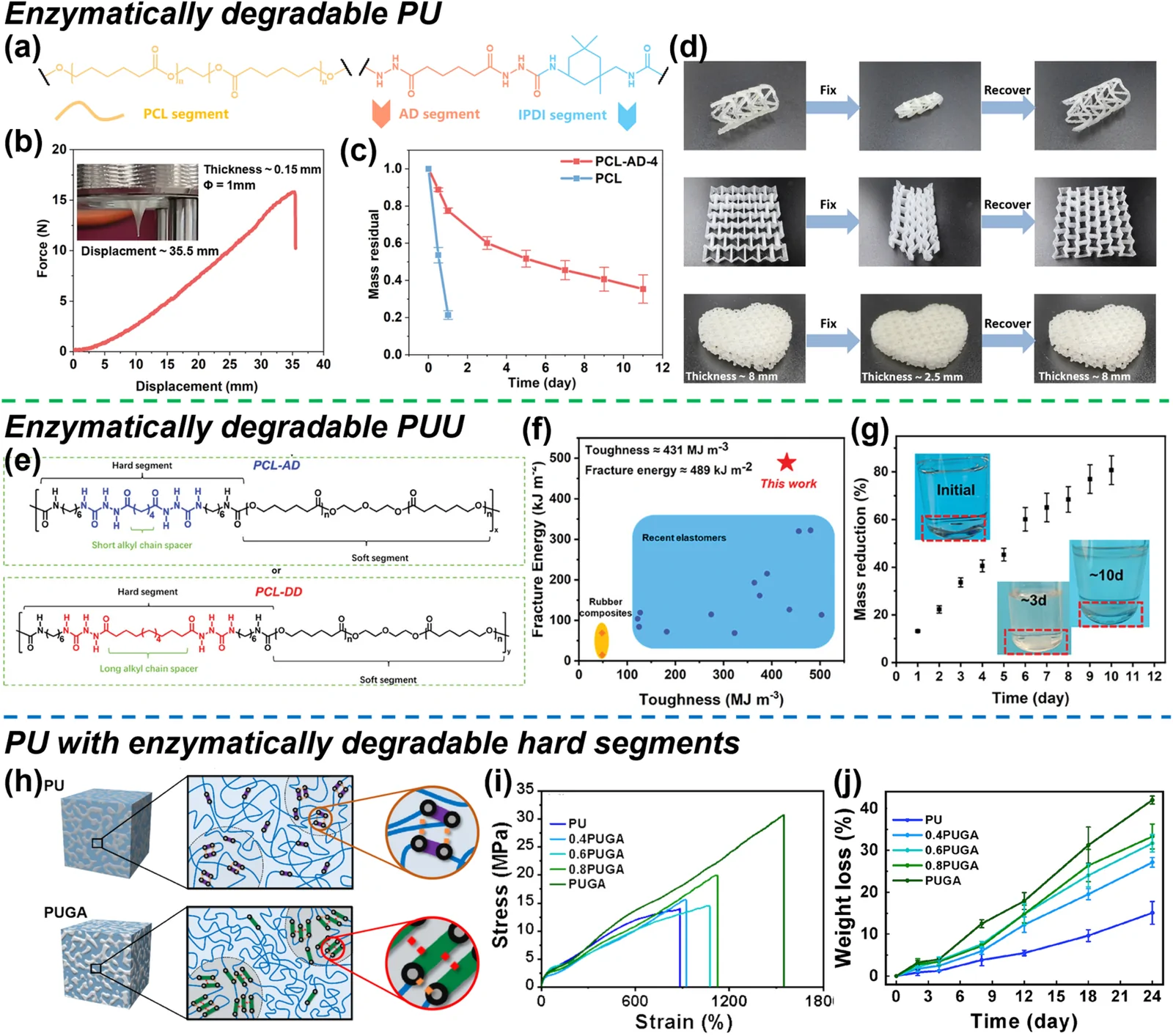
(a) Chemical structure, (b) mechanical property, (c) enzymatic degradation, and (d) shape-memory behaviors of enzymatically degradable PU. (Reproduced from ref. 235 under a CC BY-NC 4.0 license). (e) Chemical structures, (f) mechanical properties, and (g) enzymatic degradation of enzymatically degradable PUU. (Reproduced from ref. 237 under a CC BY 4.0 license). (h) Schematic polymer networks, (i) mechanical properties, and (j) enzymatic degradation of PU with enzymatically degradable hard segments. (Reproduced with permission from ref. 73. Copyright 2025, Elsevier).

Recently, Xu and co-workers prepared PCL-based PU elastomers using IPDI, and chain extender adipic dihydrazide (Fig. 9a).^235^ The optimized elastomer combined extended soft and hard segments to promote strong microphase separation, multilevel hydrogen bonding, and stretch-induced crystallization (SIC), affording exceptional mechanical properties with a true stress of 1.42 GPa, a toughness of 470 MJ m^−3^, and a fracture energy of 384.7 kJ m^−2^ (Fig. 9b). Notably, after 11 days of degradation with keratinase in phosphate buffer at 37 °C, the elastomer retained a tensile strength above 10 MPa despite ∼60% mass loss (Fig. 9c), suggesting potential as a long-term load-bearing degradable implant material. The elastomer also possessed body-temperature shape-memory behavior, 4D printability, and good biosafety, supporting potential applications in tissue repair and biomedical devices (Fig. 9d). On the other hand, Zhang and co-workers constructed transparent PCL-based PU elastomers using HDI, and imidazolidinyl urea (IU) as a multiple hydrogen-bonding chain extender.^236^ Increasing IU content increased hydrogen-bond density and stiffness but reduced toughness at high contents. The optimized PU containing 25% IU (PHI25) showed the highest toughness (168.2 MJ m^−3^) and puncture energy (72.2 mJ). PHI25 showed 50% mass loss after 49 days by lipase in phosphate buffer at 37 °C, whereas higher-IU samples degraded more slowly because of lower PCL-segment contents. They further employed IPDI, and chain extender *N*,*N*-bis(2-hydroxyethyl)oxamide to construct PCL-based PU with a more robust hydrogen-bonding network and uniformly distributed hard-segment microdomains.^76^ The optimized elastomer exhibited a tensile strength of 92.2 MPa, a toughness of 480.2 MJ m^−3^, and a fracture strain of 1932%, outperforming spider silk and most reported elastomers. However, the dense hydrogen-bonding network markedly retarded enzymatic degradation, giving only 8.3% mass loss after 98 days by lipase in phosphate buffer (pH 7.4) at 37 °C.

Building on PU structures, extension to poly(urethane-urea) (PUU) can increase hydrogen-bond density. Zhu and co-workers prepared PCL-based PUU elastomers using HDI, and either short-chain adipic dihydrazide (AD) or long-chain dodecanedioic dihydrazide (DD) as chain extenders, yielding PCL-AD and PCL-DD, respectively (Fig. 9e).^237^ Although the two elastomers contained comparable hydrogen-bond contents, PCL-DD exhibited superior performance, with a tensile strength of 63.3 MPa, a fracture strain of 1511%, a toughness of 430.9 MJ m^−3^, and a true stress of 1.01 GPa, outperforming both PCL-AD and most reported elastomers (Fig. 9f). The long alkyl spacer in DD bent the hard segments and produced smaller, more uniformly distributed hard-segment clusters, which delayed SIC to avoid stress concentration and premature fracture. Both polymers exhibited excellent recyclability and biodegradability, with PCL-DD showing ∼80% mass loss within 10 days catalyzed by lipase in phosphate buffer (pH 7.2) at 37 °C (Fig. 9g). By further increasing the HDI/DD hard-segment content, they enhanced the total number of hydrogen bonds without sacrificing bonding efficiency, thereby generating more cohesive and stable hard-segment clusters. The optimized PUU exhibited a tensile strength of 77.7 MPa, a fracture strain of 1777%, a toughness of 629.8 MJ m^−3^, and a fracture energy of 642.3 kJ m^−2^.^238^ The dense hydrogen-bonding network also endowed the material with excellent thermal stability, humidity resistance, and organic-solvent resistance, while still allowing substantial lipase-catalyzed degradation, with ∼68% mass loss within 6 days in phosphate buffer (pH 7.2) at 60 °C. The elevated degradation temperature may help weaken hydrogen-bonding interactions and promote enzymatic hydrolysis.

Introducing readily degradable moieties into the hard segment is an effective approach for improving PU degradability.^239,240^ Gan and co-workers incorporated degradable oligo(glycolic acid) (oligoGA) together with BDO as chain extenders to prepare PCL-based PUs (Fig. 9h).^73^ OligoGA modulated the hard-domain hydrogen-bonding network and increased microphase separation, making the soft and hard domains more effective in elasticity and energy dissipation. With increasing oligoGA content, tensile strength increased from 13.4 to 30.0 MPa, fracture strain increased by 51%, and toughness improved by 205% (Fig. 9i). Importantly, enzymatically cleavable oligoGA segments in the hard domain significantly accelerated degradation with *Pseudomonas* lipase in phosphate buffer at 37 °C, with mass loss increasing with oligoGA content and reaching ∼42% after 24 days and 95% after 54 days, accompanied by a decrease in the *M*_n_ of the residual polymer to ∼2600 g mol^−1^ (Fig. 9j). The material also showed body-temperature shape-memory behavior and excellent biocompatibility, highlighting their promise for absorbable implants.

In 2024, Zhang and co-workers valorized industrial waste gas COS as a valuable monomer for step polyaddition with diverse diamines and diacrylates, affording 46 poly(ester thiourethane)s.^241^ Based on segmental rigidity and thiourethane-mediated hydrogen bonding strength, these polymers span three classes. Crystalline plastics with linear hard segments and extensive hydrogen bonding exhibited high *T*_m_ of 202–274 °C, comparable to engineering plastics, and stiff mechanical behavior. Thermoplastic elastomers, obtained using softer diamines, used partial hydrogen bonding as physical crosslinks and showed good melt processability with low *T*_g_ of −43 to −10 °C, high stretchability up to 3126%, and elastic recovery. Amorphous plastics prepared from sterically hindered or secondary diamines lacked hydrogen bonding, displaying high transparency and broad thermal/mechanical tunability. Owing to their in-chain ester bonds, thermoplastic elastomers were completely dissolved by TLL in phosphate buffer (pH 7.4) at 37 °C within 10 days with the major dicarboxylic acid degradation product recovered from the solution, whereas high-*T*_m_ crystalline plastics required a higher TLL loading and 22 days.

The rational regulation of hydrogen-bonding interactions provides an effective strategy to simultaneously tailor microphase separation, mechanical performance, functionality, and enzymatic degradability in plastics. By tuning the strength, density, and distribution of hydrogen bonds, the balance between structural integrity and enzyme accessibility can be optimized. However, systematic quantification of the extent of depolymerization, comprehensive identification of degradation products, and their recovery and reutilization remain limited. Moreover, current degradable PUs still rely predominantly on polyester segments, constraining their structural diversity. Future efforts could combine hydrogen-bond engineering with the development of enzymes capable of selectively cleaving urethane linkages, thereby reducing the conventional trade-off between mechanical performance and degradability and expanding enzymatically degradable PUs beyond polyester-based designs.

### 4.2 Macrocycle-based movable crosslinks

Macrocycle-based dynamic crosslinks constructed from crown ethers, cyclodextrins (CDs), pillararenes, and related macrocycles have emerged as promising platforms for enhancing the sustainability of plastics.^221–223,242–249^ These movable crosslinking networks improve toughness and durability through energy dissipation mediated by sliding-ring effects,^250^ while their dynamic nature enables functional regulation and stimuli responsiveness, thereby expanding the practical utility of sustainable polymeric materials.

**Fig. 10:**
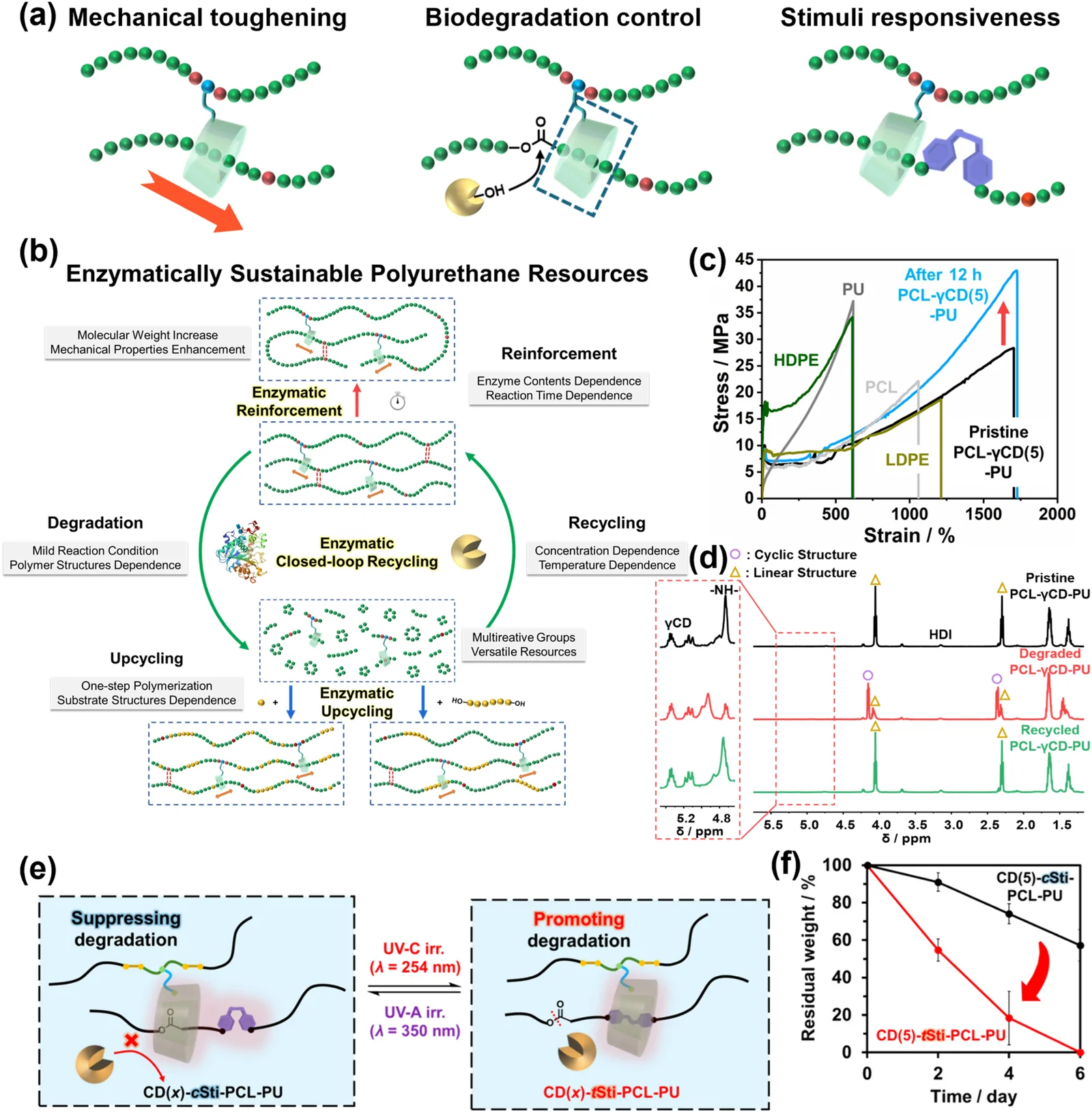
(a) Scheme illustrating the CD-based movable crosslinks in mechanical toughening, biodegradation control, and stimuli responsiveness. (b) Scheme illustrating the enzymatic reaction, (c) mechanical properties, and (d) enzymatic recycling of PCL-γCD-PU with CD-based movable crosslinks. (Reproduced from ref. 60 under a CC BY 4.0 license). (e) Scheme illustrating the photo-controlled enzymatic degradation mechanism, and (f) photo-controlled enzymatic degradation of CD-based movable crosslinks. (Reproduced with permission from ref. 251. Copyright 2026, American Chemical Society).

Our group has applied CD-based movable crosslinks into a wide range of polymeric materials,^60,66,222,245–247^ and recently extended this concept to tough materials with controllable enzymatic degradability by leveraging the bulky and dynamic features of CDs (Fig. 10a). We constructed PCL-PUs with triacetylated γ-cyclodextrin (TAcγCD)-based movable crosslinks (PCL-γCD-PU) and explored their enzymatic reactions (Fig. 10b).^60^ Compared with linear and covalently crosslinked PCL-PUs, PCL-γCD-PU exhibited substantially improved mechanical performance because the sliding-ring effect enabled efficient energy dissipation. Moreover, the degradation of PCL-γCD-PU catalyzed by Novozym@435 (immobilized CALB) in toluene was significantly enhanced with increasing TAcγCD content, as the bulky TAcγCD units introduced additional free volume into the polymer network and facilitated enzyme access to the ester bonds of the PCL segments. Through a subsequent enzymatic reinforcement strategy with precisely controlled enzymatic transesterification time, PCL-γCD-PUs with higher molecular weight were obtained, and their mechanical properties, including tensile stress and fracture strain, surpassed those of commercial HDPE, LDPE, PCL, and PU (Fig. 10c). Importantly, the degradation products, which mainly consisted of cyclic ester oligomers containing 3–5 repeating units and having molecular weights below 1000 Da, can serve as versatile feedstocks for enzymatic closed-loop recycling by varying the reaction concentration (Fig. 10d), or for enzymatic upcycling into value-added random or block copolymers by introducing selected substrates.

To further broaden this platform, we designed a series of poly(ester)-poly(urethane)s containing TAcγCD-based movable crosslinks (PEs-γCD-PUs) through structure–property tuning.^66^ By tailoring the polyol segments using a solely polyester-based poly(caprolactone) diol, a poly(ether-ester)-based poly(tetramethylene ether-caprolactone) diol, an organic-inorganic polyester-based poly(dimethylsiloxane-caprolactone) diol, or a poly(ester–ester)-based poly(caprolactone-(l-lactide)) diol, key physical properties, including thermal behavior, crystallinity, surface hydrophobicity, mechanical performance, and enzymatic degradability, could be systematically regulated. As a result, PEs-γCD-PUs spanning soft and highly stretchable elastomers, with fracture strains up to 2943% and toughness of 381 MJ m^−3^, to rigid and tough plastics, with fracture stresses of 45.3 MPa and toughness of 450 MJ m^−3^, were achieved. These tailored properties enabled diverse sustainable applications, including biodegradable hydrophobic materials, shape-memory materials, adhesives, and strain sensors. Most recently, we further designed a photoresponsive polyester comprising a PCL backbone and host-guest inclusion complexes between photoisomerizable *trans*-stilbene (tSti) units and TAcγCD units.^251^ Upon UV-A (*λ* = 350 nm) or UV-C (*λ* = 254 nm) irradiation, the stilbene units underwent reversible *trans*–*cis* isomerization, which repositioned the CD rings along the PCL backbone and modulated the molecular coverage of enzyme-active ester groups (Fig. 10e). In the trans state, the polyester segments were more exposed, accelerating lipase-catalyzed degradation and resulting in nearly complete mass loss in phosphate buffer (pH 7.0) at 37 °C, whereas in the cis state, the ester groups were shielded, thereby suppressing degradation (Fig. 10f). This switching behavior was reversible under alternating UV-A/UV-C irradiation and absent in linear control without movable rings, confirming that controllable CD positioning is essential for regulating enzymatic degradation. This photoresponsive degradation control further enabled patterned encoding with different shapes.

In addition, the incorporation of macrocycles into polymer chains to form polyrotaxanes provides another convenient strategy for tuning polymer properties and degradability.^252–254^ In this context, we upcycled cyclic polyphenylene sulfide (PPS), a waste byproduct from the production of linear PPS engineering plastics, into valuable supramolecular additives for toughening and stabilizing PS-based,^255^ and PCL-based plastics.^253^ Cyclic PPS (7U) formed rotaxane structures with PCL during bulk polymerization, generating movable crosslinks through π–π stacking interactions and nearly doubling the toughness of PU-PCL without sacrificing Young's modulus. In addition, such 7U-based movable crosslinks enabled controlled enzymatic degradation under selective conditions. In phosphate buffer (pH 7.0) at 37 °C with lipase PS, 7U improved the stability of PU-PCL by suppressing enzymatic attacks on amorphous PCL regions. By contrast, after dissolution of PU-PCL films in organic solvent, dispersion of 7U and disruption of the movable crosslinks weakened this protective effect, allowing efficient degradation by Novozym@ 435. However, the cyclic PPS employed in this study is a mixture of macrocycles, making it unclear how individual ring sizes contribute to the mechanical properties and enzymatic degradation of PCL.

Incorporating appropriate macrocycles into polymer networks provides an effective strategy for regulating mechanical properties and enzymatic degradability while imparting additional functionalities. Notably, only small amounts of macrocycles are generally required, allowing them to exert substantial structural and functional effects without markedly increasing the chemical complexity of the degradation products or hindering their potential recovery and reuse. Nevertheless, research on such macrocycle-based plastics remains at an early stage. A deeper understanding of how these macrocycles regulate enzymatic degradation, together with the development of efficient and scalable manufacturing processes, will be essential for advancing these materials as a new generation of smart and sustainable plastics.

### 4.3 Metal–ligand coordination

Dynamic metal–ligand coordination is a powerful and highly specific supramolecular interaction that enables precise regulation of material structure and properties through judicious selection of metal ions and ligands.^225,256–258^ As summarized in Section 2.1, incorporating appropriate metal–ligand coordination into protein- and DNA-based bioplastics can enhance mechanical performance while preserving excellent biodegradability, highlighting its promise for sustainable plastics.^97,112^

For synthetic plastics, Wang and co-workers synthesized poly(propanediol-HPA-sebacate) (PAS) by polycondensation of sebacic acid, 1,3-propanediol, and the Schiff-base diol (HPA), leaving the phenolic oxygen and Schiff-base group as pendant groups.^259^ Two or three such ligands coordinated tetra- or hexacoordinate biologically relevant metal ions, including Mg^2+^, Ca^2+^, Fe^3+^, Co^2+^, Cu^2+^, and Zn^2+^, to form metal–ligand crosslinks. By tuning the metal identity, metal/ligand ratio, and ligand density, the network structure, mechanical properties, and hydrolytic stability could be broadly regulated. Accelerated hydrolysis further revealed metal-dependent degradation, reflecting differences in metal–ligand chelation strength. The Fe^3+^-chelated elastomer showed cytocompatibility comparable to PCL, supporting HUVEC adhesion, spreading, and metabolic activity *in vitro*. In a mouse subcutaneous implantation model, Fe^3+^-crosslinking foams degraded faster than PCL while eliciting comparable or milder inflammatory and fibrotic responses, demonstrating their promise for soft-tissue reconstruction and biomedical applications. Zhu and co-workers developed PCL-based elastomers using protocatechualdehyde (PA) as a catechol ligand to yield metal–ligand coordination with Fe^3+^.^260^ The phase-separated structure, strong Fe^3+^-catechol coordination, and SIC acted synergistically to dissipate energy, affording a high toughness of ∼372 MJ m^−3^ and a record fracture energy of ∼646 kJ m^−2^. The dynamic coordination bonds enabled solvent-assisted recycling for at least three cycles without mechanical loss, while retaining biodegradability. The elastomer was degradable by lipase in phosphate buffer (pH 7.2) at room temperature, showing >70% mass loss within 10 days and ∼97% within 2 months. Moreover, the elastomer exhibited good biocompatibility and promoted wound healing in mice when used as sutures.

## 5. Conclusion and perspectives

In conclusion, rational plastics design through chemical design, physical blending, and supramolecular interactions offers powerful opportunities to overcome the persistent trade-off between performance and degradability in biodegradable plastics. Chemical design can advance the development of sustainable plastics through the modification of biopolymers, the selection of eco-friendly feedstocks, and precise control over polymer sequence and architecture. Physical blending provides a versatile platform for integrating different polymers and enabling the compatible incorporation of enzymes or living biological systems into polymer matrices through appropriate blending strategies. Supramolecular interactions, including hydrogen bonding, macrocycle-based crosslinks, and metal coordination, provide dynamic and smart regulation of polymer structures and properties. Together, these strategies enable the optimization of biodegradable plastics across multiple structural levels and from complementary perspectives. They not only improve the mechanical properties, functionality, and enzymatic degradability of existing biodegradable plastics but also facilitate the development of advanced smart and sustainable plastics for emerging applications in packaging, electronics, and biomedicine. However, despite this progress, high-performance and enzymatically degradable plastics still face several critical challenges before practical implementation:

(1) Practical and scalable production of plastics. Most reported systems are still developed at the laboratory scale, and their industrial manufacturability, production cost, processing robustness, and long-term reliability remain insufficiently established. Moreover, several systems rely on multistep syntheses or specialized monomers, which may limit large-scale production. Future studies should therefore focus on developing simpler and more scalable synthetic routes, while ensuring compatibility with existing polymer-processing technologies, to determine whether these materials can be manufactured economically and practically at an industrial scale.

(2) Quantification and characterization of plastic degradation. In most current studies, enzymatic degradation is primarily evaluated by mass loss, molecular weight reduction, and surface morphology changes. Although convenient and widely used, these methods cannot independently and quantitatively distinguish chain scission, fragment solubilization, and complete depolymerization, nor do they provide sufficient information on the chemical identity, yield, and distribution of the resulting monomers, oligomers, and other soluble products. Consequently, the degradation pathways and fate of these products remain poorly understood. Future studies should combine conventional measurements with product-focused analyses, including NMR spectroscopy, chromatography, mass spectrometry, and toxicity assessments, to enable more reliable quantification of enzymatic degradation and establish standardized and comparable degradation metrics.

(3) Separation, recovery, and reutilization of degradation products. Owing to the potential substrate and bond selectivity, controlled enzymatic degradation may facilitate the generation of more defined products. Nevertheless, only a limited number of studies have demonstrated the isolation of degradation products or reused them for recycling or upcycling. Whether enzymatic degradation yields reusable monomers and oligomers, or complex mixtures requiring intensive purification therefore remains unclear for many plastics systems. Future studies should integrate plastics degradation with downstream separation, purification, and product valorization, while evaluating recovery yield, product purity, and the properties of regenerated materials. The recovery and repeated use of enzymes should also be considered to improve the economic and environmental sustainability of the overall process.

## Author contributions

J. Liu conceived the review, collected and analyzed the literature, wrote and revised the manuscript. X. Zhou contributed to manuscript preparation, literature organization, and manuscript revision. H. Uyama provided valuable suggestions on manuscript preparation and revision. Y. Takashima supervised the project, guided the overall structure and scientific direction of the review, and contributed to manuscript revision.

## Conflicts of interest

The authors declare no competing interests.

## Data Availability

No primary research results, software or code have been included and no new data were generated or analysed as part of this review.
